# Identification of drivers of breast cancer invasion by secretome analysis: insight into CTGF signaling

**DOI:** 10.1038/s41598-020-74838-8

**Published:** 2020-10-21

**Authors:** Johanna W. Hellinger, Franziska Schömel, Judith V. Buse, Christof Lenz, Gerd Bauerschmitz, Günter Emons, Carsten Gründker

**Affiliations:** 1 Department of Gynecology and Obstetrics, University Medicine Göttingen, Robert-Koch-Str. 40, 37075 Göttingen, Germany; 2 Institute of Clinical Chemistry, University Medical Center Göttingen, Göttingen, Germany

**Keywords:** Oncology, Cancer

## Abstract

An altered consistency of tumor microenvironment facilitates the progression of the tumor towards metastasis. Here we combine data from secretome and proteome analysis using mass spectrometry with microarray data from mesenchymal transformed breast cancer cells (MCF-7-EMT) to elucidate the drivers of epithelial-mesenchymal transition (EMT) and cell invasion. Suppression of connective tissue growth factor (CTGF) reduced invasion in 2D and 3D invasion assays and expression of transforming growth factor-beta-induced protein ig-h3 (TGFBI), Zinc finger E-box-binding homeobox 1 (ZEB1) and lysyl oxidase (LOX), while the adhesion of cell-extracellular matrix (ECM) in mesenchymal transformed breast cancer cells is increased. In contrast, an enhanced expression of CTGF leads to an increased 3D invasion, expression of fibronectin 1 (FN1), secreted protein acidic and cysteine rich (SPARC) and CD44 and a reduced cell ECM adhesion. Gonadotropin-releasing hormone (GnRH) agonist Triptorelin reduces CTGF expression in a Ras homolog family member A (RhoA)-dependent manner. Our results suggest that CTGF drives breast cancer cell invasion in vitro and therefore could be an attractive therapeutic target for drug development to prevent the spread of breast cancer.

## Introduction

Metastasis is second leading cause of cancer-related death in the US. Barely 27% of breast cancer patients diagnosed with distant metastasis survive a period of 5 years^1^. Breast cancer mortality will increase by 46.5% until 2040 to almost 1 million deaths worldwide^2^. Single most frequent site for breast cancer metastasis is bone, which accounts for 70% of all metastatic breast cancer^3^. Elucidation of drivers of cancer metastasis is therefore pivotal. The metastatic cascade is initiated by dissemination of cancer cells into surrounding tissue^4^. Micro environments of primary tumor and metastatic niche have shared communication networks. Tumor stroma stiffness facilitates deposition and remodeling of extracellular matrix (ECM) in breast cancer^5–7^. Cancer cells, cancer associated fibroblasts, and immune cells modulate ECM by deposition of structural components like collagens or fibronectin (FN1), secretion of growth factors (e.g. Transforming growth factor-beta-induced protein ig-h3, connective tissue growth factor) and ECM-transforming enzymes (e.g. Lysyl oxidase)^6^. Gene expression studies identified a bone metastatic signature which includes expression of connective tissue growth factor (CTGF) and is associated with poor patient outcome and metastasis^8,9^. Cancer cells use developmental processes like epithelial-mesenchymal transition (EMT) to gain invasive properties and stemness. These properties help them to disseminate, intravasate, circulate, extravasate and retain during dormancy. After that, the cancer cells are in need of mesenchymal-epithelial transition (MET) to reactivate upon cues from metastatic niche and outgrow^10^. This theory is consistent with observations that clinical samples of human metastasis resemble epithelial phenotype of primary tumors^11^. There is an urgent need to identify potential drivers of cell invasion, the initial step within metastatic cascade, at the primary site and colonization at distant sites. A better understanding of transient dynamic processes of high cellular plasticity could help to intercept the metastatic cascade, which could in turn lead to identification of targets for new treatment options to prevent cancer cell dissemination and metastatic outgrowth. We aim to identify secreted proteins priming micro environment resulting in increased cancer cell dissemination and driving epithelial-mesenchymal transition.

We combined co-culture model for bone-directed breast cancer cell invasion with mass spectrometry based secretome analysis and identified secreted CTGF as potential driver for breast cancer cell invasion. In this system, CTGF was found to regulate cell-ECM adhesion, proteolytic activity and expression of EMT inducing genes. Moreover, CTGF expression is dependent on RhoA activity. In addition, treatment of invasive breast cancer cells with gonadotropin releasing hormone (GnRH) agonist Triptorelin could increase RhoA activity. These data indicate, that CTGF is a promising target to inhibit invasion in highly plastic breast cancer cells and aggressive triple negative breast cancer (TNBC) cells.

## Results

### Identifying potential drivers of breast cancer cell invasion

**Figure 1:**
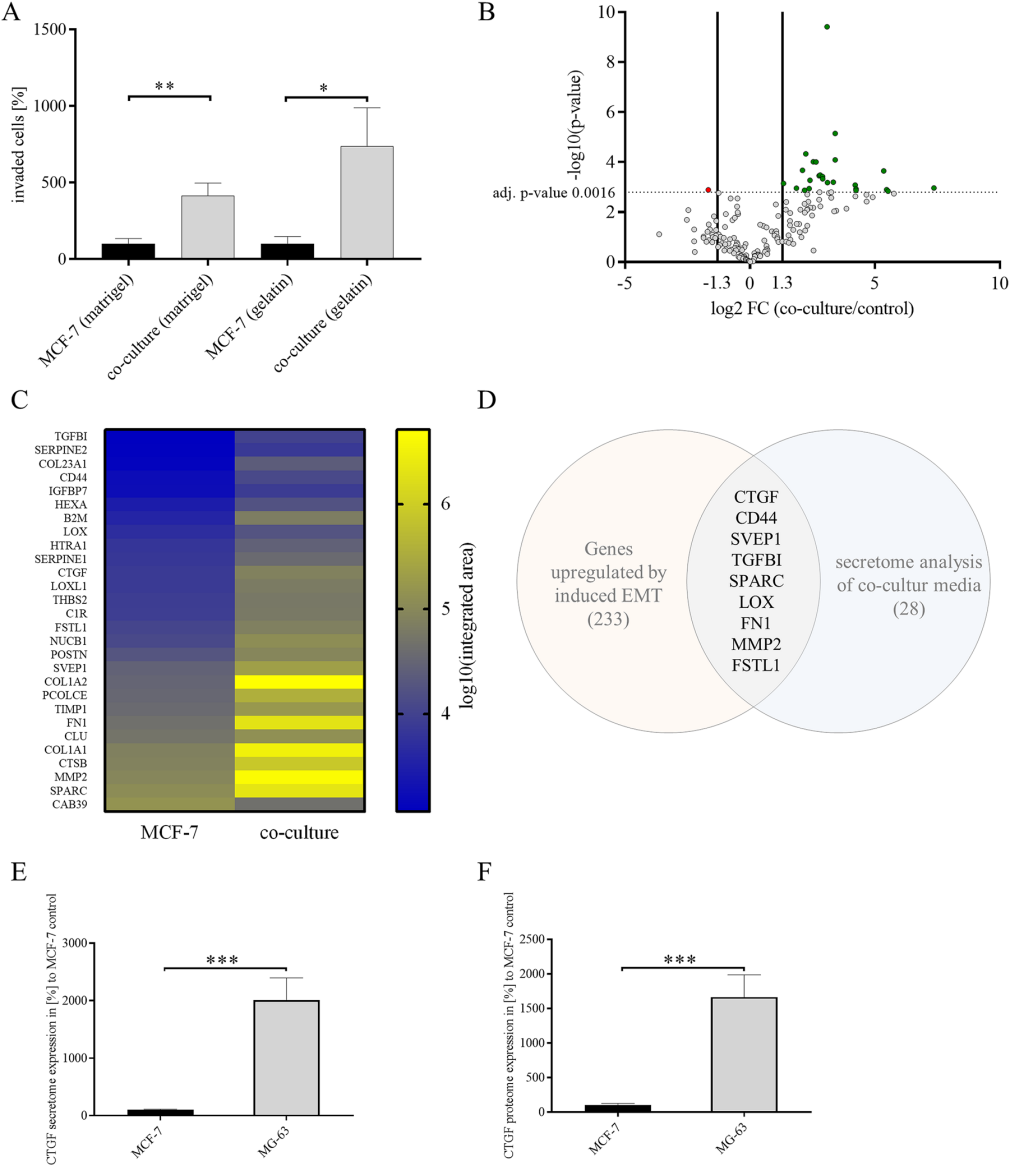
Identifying drivers of breast cancer cell invasion. (**A**) Transwell-invasion co-culture assay of MCF-7 breast cancer cells and MG-63 osteosarcoma cells without FBS addition and Matrigel or Collagen I coated insert. Invaded cells under the filter were stained and counted in four randomly selected regions after 96 h in co-culture. Data represent mean ± SEM. MCF-7 (Matrigel) n = 12, MCF-7 (gelatin) n = 6, unpaired, two-sided t-test to respective control. *P < 0.05; **P < *0.01* (**B**) Volcano plot demonstrating potential bone-directed breast cancer invasiveness related targets using secretome analysis. Detected target proteins were stated as discovery when adjusted p-value (adj. p-value) was below 0.0016 (dotted line) with a false-discovery rate (FDR) of 1% and a log twofold change (FC) higher 1.3 or lower -1.3. Every dot indicates one target, green dots indicate upregulated discoveries and red dot indicates downregulated discovery. n = 6, discovery determined using the two-stage linear step-up procedure of Benjamini, Krieger and Yekutieli, with Q = 1%. Each row was analyzed individually, without assuming a consistent SD. (**C**) Heat map visualizing all discoveries with a color gradient of log10 integrated area of mean values of three biological and two technical replicates corresponding to B. (**D**) Scheme of overlapping targets from microarray analysis of MCF-7 cells under dynamic EMT program and secretome analysis of co-cultured MCF-7 cells with a fold change of higher 1.3 or lower -1.3 and FDR 5% (microarray) and FDR 1% (secretome analysis). (**E**) Comparison of CTGF expression in the secretome of MCF-7 and MG63 cells. Data represent mean ± SEM. n = 6 using unpaired, two-tailed t-test analysis to MCF-7 (= 100%). ***P < *0.001* (**F**) Comparison of CTGF expression in the proteome of MCF-7 and MG63 cells. Data represent mean ± SEM. n = 6 using unpaired, two-tailed t-test analysis to MCF-7 (= 100%). ***P < *0.001b.*

Up to 13.6% of breast cancer patients (diagnosed in stage I-III) will develop bone metastasis within 15 years of follow-up^12^. Previous studies have demonstrated that co-culture of breast cancer cells with osteosarcoma cells (MG-63) or osteoblast-like cells increased invasiveness^13^. However, mechanisms by which breast cancer cells metastasize to bone remain elusive. To shed light on drivers for bone-directed breast cancer invasion, we decided to investigate if identified potential drivers by analyzing secretome of co-culture media using mass spectrometry. Excluding serum from media and analyzing only secreted proteins, we first tested if non-invasive MCF-7 breast cancer cells gain invasive properties when co-cultured with osteosarcoma cells without adding serum to media (Fig. 1A). Indeed, invasiveness of MCF-7 breast cancer cells increased more than fourfold, when using Matrigel in a transwell co-culture invasion assay (Fig. 1A; co-culture Matrigel: 413.7 ± 83.07% vs. MCF-7 Matrigel; *P* = 0.0021, n = 12) and a more than sevenfold increase of invasiveness, when using gelatin (Fig. 1A; co-culture gelatin: 737.5 ± 250.9% vs. MCF-7 gelatin; *P* = 0.0316, n = 6). We next co-cultured MCF-7 cells with MG-63 cells and analyzed co-culture media using mass spectrometry secretome analysis to identify proteins that drive bone-directed metastasis. We could identify 28 secreted potential drivers for bone-directed breast cancer cell invasion (Fig. 1B, C, and supplementary table 1). Gene ontology (GO) enrichment analysis elucidated that observed secreted proteins play most prominently roles in extracellular matrix organization (supplementary table 2, FDR 3.26 × 10^–15^; 50% of detected proteins), extracellular structure organization (supplementary table 2, FDR 1.52 × 10^–14^; 50% of detected proteins) and wound healing (supplementary table 2, FDR 8.92 × 10^–9^; 39% of detected proteins). Further classification of observed proteins using Shiny GO indicated that 39% of detected proteins are categorized within locomotion and cell motility and 36% within cell adhesion (supplementary table 3, supplementary Fig. 1A). Additionally, we could detect that co-culture media in comparison to MG-63 media a decreases MMP2 protein expression and an increased SPARC expression was detected (supplementary Fig. 1B). To further examine underlying molecular mechanism of breast cancer cell invasion we analyzed lysates of co-cultured MCF-7 cells compared to untreated MCF-7 cells (supplementary Fig. 1, supplementary table 4). GO enrichment analysis elucidated that observed regulated proteins play most prominently roles in protein folding (supplementary table 5, FDR 7.55 × 10^-6^; 33% of detected proteins), programmed cell death (supplementary table 5, FDR 1.34 × 10^-5^; 61% of detected proteins) and cellular response to cytokine stimulus (supplementary table 5, FDR 1.34 × 10^-5^; 50% of detected proteins). Interestingly, cell death associated proteins seem to be regulated prominently, including HSPA9 (heat shock protein family A (Hsp70) member 9), HSP90B1 (heat shock protein 90 β family member 1), HSP90AB1 (heat shock protein 90 α family class B member 1), HSPD1 (heat shock protein family D (Hsp60) member), and HSPB1 (heat shock protein family B member 1) (supplementary Fig. 1D, supplementary tables 4 and 5). While detected findings from proteome analysis are different from detected secretome findings, GO grouping of proteome findings elucidated similar results compared to secretome findings. Proteome analysis findings where categorized (amongst others) in 33% locomotion, 33% cell motility and 27% cell adhesion (supplementary table 6).

Cells undergoing dynamic EMT programs reveal an increased invasive behavior^14^. Microarray analysis of MCF-7 breast cancer cells within a dynamic TGFβ-dependent EMT program exhibited an increased expression of CTGF, CD44 molecule (CD44), Sushi, von Willebrand factor type A, EGF and pentraxin domain containing 1 (SVEP1), Transforming growth factor-beta-induced protein ig-h3 (TGFBI), Secreted Protein Acidic And Cysteine Rich (SPARC), Lysyl oxidase (LOX), FN1 and Matrix Metallopeptidase 2 (MMP2) and Follistatin-like 1(FSTL1)^14^. Interestingly, we found these proteins are highly secreted in co-culture medium of MCF-7 and MG-63 as elucidated by secretome analysis (Fig. 1D, supplementary table 1). An additional secretome analysis showed that MG-63 cells secrete a 20-fold higher amount of CTGF into the medium as compared with MCF-7 cells (Fig. 1E; 2010.0 ± 385.5% vs. MCF-7, *P* = 0.0006, n = 6). Proteome analysis also showed a significantly stronger CTGF expression of the MG-63 cells which was 16 times higher than with MCF-7 cells (Fig. 1F; 1664.1 ± 322.8% vs. MCF-7, *P* = 0.0007, n = 6).

### CTGF expression correlates with invasiveness of mesenchymal transformed and TNBC cells

**Figure 2:**
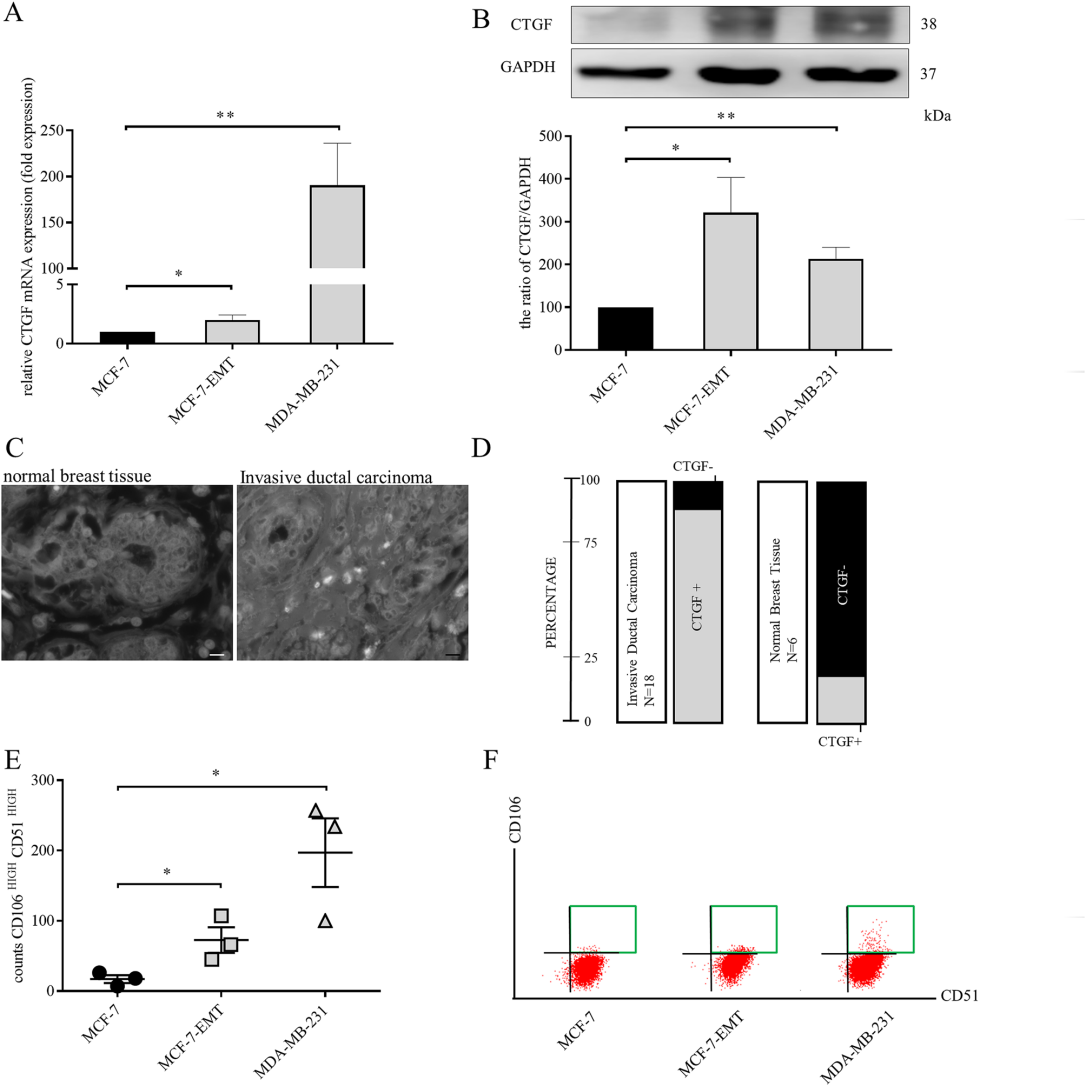
CTGF expression correlates with invasiveness of mesenchymal transformed and TNBC cells. (**A**) Assessment of CTGF mRNA expression in different breast cancer cell lines using quantitative real-time PCR. Data represent mean ± SEM. MCF-7-EMT n = 6, MDA-MB-231 n = 4 using unpaired, two-tailed t-test analysis to respective control (MCF-7). *P < 0.05; **P < *0.01* (**B**) Quantification and representative experiments of CTGF protein expression in different breast cancer cell lines compared to non-invasive MCF-7 breast cancer cell line. CTGF band intensity was quantified by densitometry and normalized to GAPDH. Lower panel shows loading control GAPDH that was detected in the same sample and were run in the same gel lane and detected in the same Western blot membrane. Data represent mean ± SEM. n = 6 using unpaired, two-tailed t-test analysis to respective control (MCF-7). *P < 0.05; **P < *0.01* (**C**) Patient tissue sections (n = 24) were analyzed for CTGF expression. Representative images of normal breast tissue (right panel) and IDC (invasive ductal carcinoma, left panel) are illustrated. (**D**) Graph illustrating distribution of CTGF expression within two different analyzed patient sample categories. (**E**) Results of three independent flow cytometry experiments of CD51 and CD106 co-expression in MCF-7 (circle), MCF-7-EMT (square) and MDA-MB-231 (triangle) breast cancer cell lines. Data represent mean ± SEM. MCF-7-EMT, MDA-MB-231 n = 3 using unpaired, two-tailed t-test analysis to respective control (MCF-7). *P < *0.05* (**F**) Proportion of CD51 to CD106 was asses using flow cytometry after staining with fluorescence-labeled antibodies. A representative experiment to E is illustrated.

One of the potential drivers of invasion is CTGF, which is upregulated during wound healing and has an impact on osteolytic breast cancer metastasis^15,16^. Using patient data from large public cancer genomic datasets CTGF expression was assessed in bone, lung, liver, and brain where breast cancer spreads most prominently^17^. Expression of CTGF in bone and lung appeared to be close to expression in breast tissue, while expression in brain and liver is reduced compared to breast (Fig. 2 and supplementary Fig. 2). CTGF mRNA expression is upregulated in mesenchymal transformed (Fig. 2A; MCF-7-EMT: 1.995 ± 0.4356 vs. MCF-7; *P* = 0.0454; n = 6) and TNBC cells (Fig. 2A; MDA-MB-231: 190.5 ± 45.81 fold change vs. MCF-7; *P* = 0.0061; n = 4). Protein expression analysis gave similar results (Fig. 2B and S 9; MCF-7-EMT: 321 ± 82.6% vs. M; *P* = 0.0233; n = 6 and MDA-MB-231: 213 ± 27.17%; *P* = 0.002; n = 6).

To verify the potential use of CTGF as a therapeutic target for invasive breast cancer we first analyzed 24 breast tissue sections. Of these, 18 (75%) were invasive ductal carcinomas and 16 (88.9%) exhibit a positive signal (Fig. 2C, D and supplementary table 7a) for CTGF while 5 (83.3%) of the 6 analyzed normal breast tissues were negative for CTGF (Fig. 2C, D and supplementary table 7a).

In a second analysis, we tested 94 tissue sections of 47 patients (2 samples per patient) including non-cancerous tissues to analyze whether CTGF expression correlates with expression of androgen (AR), estrogen (ER), progesterone (PR) receptors or epidermal growth factor receptor 2 (HER2) (supplementary table 7b). Of these tissues, 3 were normal breast, 1 periductual mastitis, 3 hyperplasias, 2 fibrocystic changes, 3 fibroadenomas, 29 invasive ductal carcinomas, 1 phyllodes sarcoma, 2 intraductal carcinomas, 1 ductal carcinoma in situ, 1 invasive mucinous adenocarcinoma and 1 invasive lobular carcinoma. Two of the normal breast tissues showed no and 1 normal breast tissue a weak expression of CTGF (33.3%). It is notable that it is the normal breast tissue with increased expression of HER2 which is also slightly positive for CTGF. A positive correlation between HER2 and CTGF expression was recently found in gallbladder cancer and matched adjacent normal tissue^18^. There is also evidence in breast cancer that CTGF has an association with resistance to Lapatinib, a HER2-targeting therapeutic^19^. Twenty-eight (96.5%) of the 29 invasive ductal carcinomas showed CTGF expression. All other above mentioned tissues showed CTGF expression. Apart from the indication for a possible positive correlation between HER2 and CTGF in one normal breast tissue, no recognizable correlations between expression of CTGF and of AR, ER, PR or HER2 were found.

In a third series with 104 breast cancer tissue samples and their matched lymph node metastases tissues, we analyzed whether expression of CTGF in metastatic breast cancer tissues correlates with expression in matched lymph node metastases (supplementary table 7c). Of these cancer/metastatic tissue pairs, 86 were invasive ductal carcinomas, 8 invasive lobular carcinomas, 1 medullary carcinoma, 1 invasive micropapillary carcinoma and 8 mixed carcinomas (invasive ductal and invasive lobular carcinoma) including the matched metastatic tissue. Eighty-one (94.2%) of the 86 invasive ductal carcinomas including their matched metastatic tissue showed CTGF expression. In 3 of these cases (3.7%), CTGF expression in the metastatic tissue was clearly higher than in the primary tumor tissue. In 5 cases (5.8%), CTGF was not detectable either in the invasive ductal carcinoma or in the matched metastatic tissue. All of the 8 invasive lobular carcinomas, the medullary carcinoma, the invasive micropapillary carcinoma and the 8 mixed carcinomas including their matched metastatic tissues showed CTGF expression. In 1 case (12.5%) of the 8 invasive lobular carcinomas and in 2 cases (25%) of the 8 mixed carcinomas, CTGF expression in the matched metastatic tissue was clearly higher than in the primary tumor tissue. Noticeable correlations between CTGF expression and expression of ER, PR and HER2 were not found.

Detection of mesenchymal transformed and aggressive breast cancer cells is a major requirement to select specific treatment options. Previously, it was demonstrated that cells in transient transitional stages express specific cell receptor markers^20^. We found, that highly plastic breast cancer cells and TNBC do not only express more CTGF but co-express CD106 (Vascular cell adhesion molecule 1) and CD51 (Integrin subunit alpha V) in a higher probability than non-invasive MCF-7 cells (Fig. 2E, F and supplementary Fig. 4; MCF-7-EMT 72.67 ± 18.21 counts CD106^high^ CD51^high^ vs. MCF-7; *P* = 0.043; n = 3; MDA-MB-231 197 ± 49 counts CD106^high^ CD51^high^ vs. MCF-7; *P* = 0.0217, n = 3).

**Figure 3:**
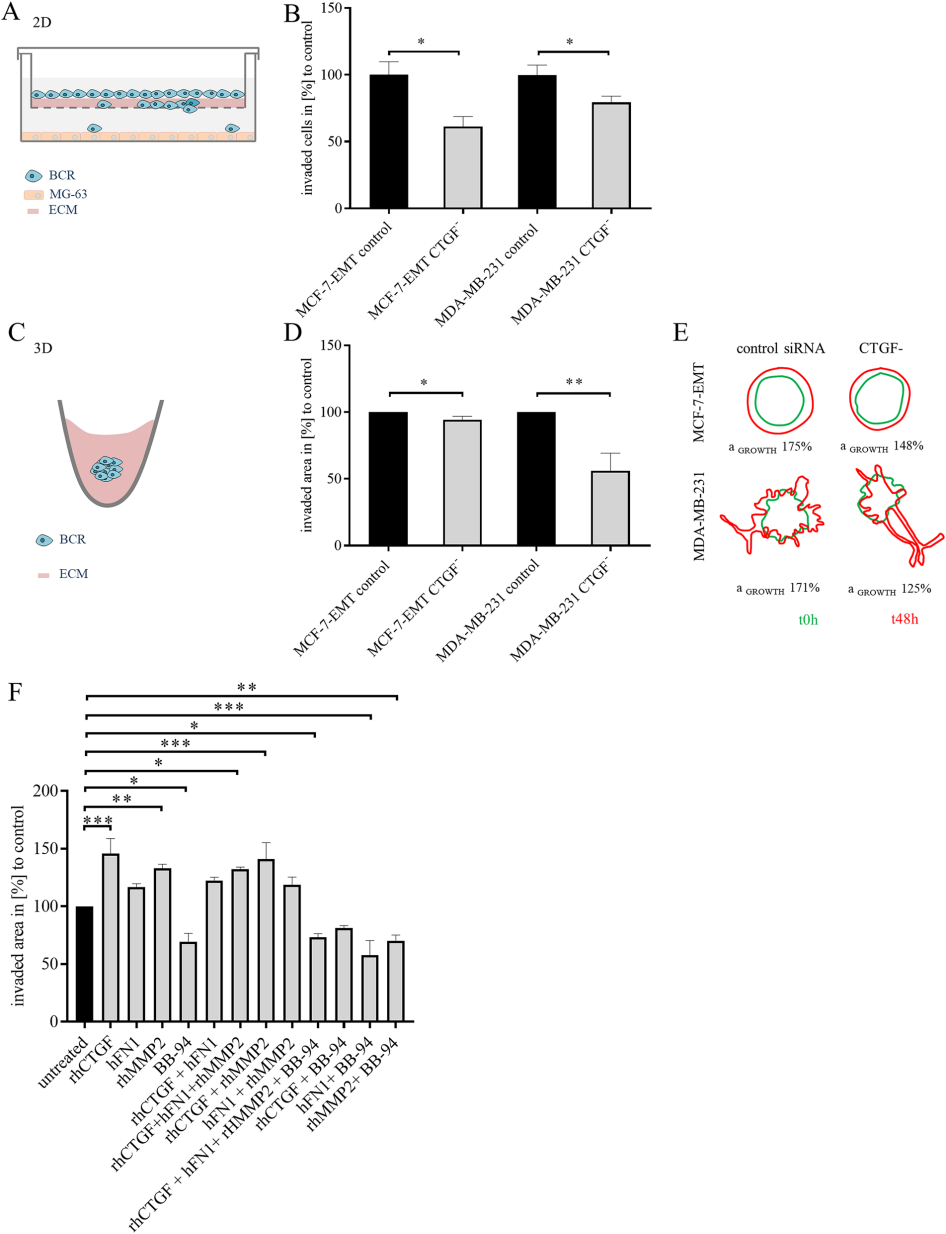
CTGF regulates invasiveness in breast cancer cells. (**A**) Scheme illustrating 2D invasion experiment using a co-culture transwell invasion assay. BCR = breast cancer cell, ECM = extracellular matrix. (**B**) Following CTGF siRNA transfection invaded cells under filter were counted in four randomly selected regions, using a co-culture Matrigel invasion assay for 96 h. Data represent mean ± SEM. MCF-7-EMT n = 15, MDA-MB-231 n = 9 using unpaired, two-tailed t-test analysis to respective control. *P < *0.05.* (**C**) Scheme illustrating 3D spheroid invasion assay. Cells were seeded in ultra-low attachment wells and after initial spheroid formation (48 h), spheroids were surrounded by Matrigel matrix and further cultivated. (**D**) 3 D spheroid assay was performed after transient CTGF siRNA transfection. Invaded area was assessed using ImageJ software and relative area growth was calculated corresponding to respective control. Data represent mean ± SEM.MCF-7-EMT n = 15, MDA-MB-231 n = 9 using unpaired, two-tailed t-test analysis to respective control. *P < 0.05; **P < *0.01. *(**E**) Representative experiment illustrating area measurement of 3D spheroids. Green shape corresponding to initial spheroid size right after adding Matrigel and red shape corresponding to time point 48 h after Matrigel adding. (**F**) 3 D spheroid assay of MCF-7 cells treated with different combinations of 1 µg/ml rhCTGF, 1 µg/ml hFN1, 1 µg/ml rhMMP2, and/or 4 nM BB-94 (Batimastat) for 48 h every 24 h. Area growth of spheroids was assessed using ImageJ software and relative area growth was calculated corresponding to untreated control. Data represent mean ± SEM. n = 4–6 using one-way ANOVA and a Dunnett ‘s multiple comparison test with no matching or pairing between groups was calculated to assess significant differences compared to untreated control. *P < 0.05; **P < 0.01; ***P < *0.005*. The graphics A, C, E were created using Microsoft PowerPoint 2016; www.microsoft.com.

Findings from secretome and proteome analyses prominently grouped into locomotion and cell motility categories. Therefore, we assessed impact of CTGF expression on invasiveness of mesenchymal transformed and TNBC cells. Using RNA interference CTGF expression was transiently suppressed (supplementary Fig. 5A, B). Suppression of CTGF leads to reduced invasion of mesenchymal transformed (Fig. 3A and B; MCF-7-EMT 61.41 ± 7.427% vs control; *P* = 0.0034; n = 18) and TNBC (Fig. 3B; MDA-MB-231 79.44 ± 4.64% vs control; *P* = 0.0258; n = 17) cells in 2D transwell invasion co-culture assay. Recent reports suggested that YAP-activation appears differently dependent on dimension model used^21^. CTGF is transcriptional expressed upon YAP translocation to nucleus. We therefore tested, if effects were reproducible in 3D invasion assay setup (Fig. 3C). Reducing CTGF expression transiently reduced invaded area in 3D breast cancer spheroids of mesenchymal transformed breast cancer cells (Fig. 3D and E; MCF-7-EMT 94.25 ± 2.535% vs control; *P* = 0.032; n = 15) and TNBC cells (Fig. 3D and E; MDA-MB-231 55.93 ± 13.3% vs control; *P* = 0.0044; n = 9).

Upon co-culturing breast cancer cells with osteosarcoma cells, cells gain invasive potential and exhibit a specific expression profile. It was suggested earlier, that an increased CTGF expression alters expression of matrix metalloproteinases and MMP-2 promotes migration by cleaving fibronectin and CTGF^22–24^. It remained unclear though, whether extracellular MMP2, CTGF and FN1 facilitate invasion in breast cancer interdependently. Consequently, we analyzed if spheroid invaded area and proliferation were altered when treated with human MMP2, CTGF and FN1. Furthermore, we analyzed if treatment with an MMP2 inhibitor (BB-94, Batimastat) reduces breast cancer invasiveness. We found that 3D spheroid area growth was increased when treated with recombinant human CTGF (rhCTGF; Fig. 3F; rhCTGF: 135.5 ± 35.5% mean difference vs. untreated; *P* = 0.0006; F = 21.61; n = 6), recombinant human MMP2 (rhMMP2, Fig. 3F; rhMMP2: 137.8 ± 37.8% mean difference vs. untreated; *P* = 0.0003; F = 21.61; n = 6), rhCTGF and human FN1 (hFN1) and recombinant human MMP2 (Fig. 3F; rhCTGF + hFN1 + rhMMP2: 137 ± 37% mean difference vs. untreated; *P* = 0.0003; F = 21.61; n = 6), or rhCTGF and rhMMP2 (Fig. 3F; rhCTGF + rhMMP2: 137.2 ± 37.2% mean difference vs. untreated; *P* = 0.0015; F = 21.61; n = 4). Adding hFN1, combination of hFN1 with rhCTGF or rhMMP2 does not alter spheroid area growth (Fig. 3F). In contrast, combining hFN1 and BB-94 treatment (Fig. 3F; hFN1 + BB-94: 68.4 ± 31.6% mean difference vs. untreated; *P* = 0.0028; F = 21.61; n = 4), or rhMMP2 and BB-94 (Fig. 3F; rhMMP2 + BB-94: 69.4 ± 30.1% mean difference vs. untreated; *P* = 0.0041; F = 21.61; n = 6) or rhCTGF and hFN1 and rhMMP2 and BB-94 (Fig. 3F; rhCTGF + hFN1 + rhMMP2 + BB-94: 75.66 ± 24.34% mean difference vs. untreated control; *P* = 0.0006; F = 21.61; n = 6) results in decreased area growth. Combination of rhCTGF with BB-94 did not alter invasive area growth. While none of the settings altered proliferation (supplementary Fig. 5C, D).

### CTGF alters cell-ECM adhesion and proteolytic activity of breast cancer cells

**Figure 4:**
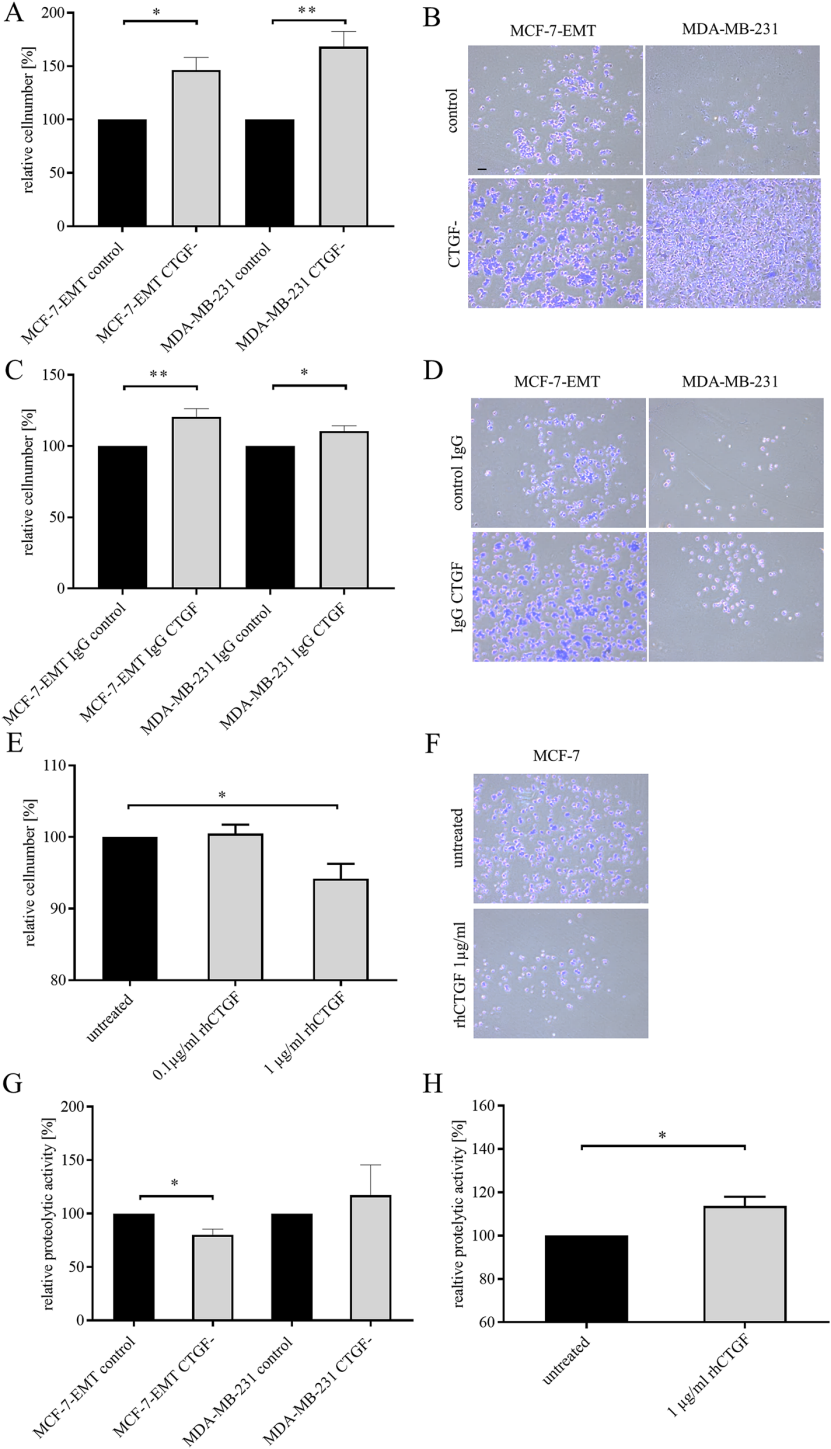
CTGF alters cell-ECM adhesion and proteolytic activity of breast cancer cells. (**A**) Adhesion analysis of transiently transfected mesenchymal transformed and triple-negative breast cancer cells. Adhesive cells where counter-stained with crystal violet and absorption was measured at 570 nm. Data represent mean ± SEM. MCF-7-EMT n = 3, MDA-MB-231 n = 3 using unpaired, two-tailed t-test analysis to respective control. *P < 0.05; **P < *0.01* (**B**) Representative images corresponding to A. (**C**) Extracellular CTGF was reduced using a blocking-antibody against CTGF and cell-ECM adhesion was assessed. Data represent mean ± SEM. MCF-7-EMT n = 6, MDA-MB-231 n = 3 using unpaired, two-tailed t-test analysis to respective control (IgG control). *P < 0.05; **P < *0.01* (**D**) Representative images corresponding to C. (**E**) MCF-7 cells where treated with recombinant human CTGF (rhCTGF) in different concentrations prior to assessing of cell-ECM adhesion. Data represent mean ± SEM. n = 3 using one-way ANOVA with F = 6.244 and a Dunnett ‘s multiple comparison test with no matching or pairing between groups. *P < *0.05* (**F**) Representative images corresponding to E. (**G**) Following transient transfection mesenchymal transformed and triple negative breast cancer cells were seeded on FITC-conjugated gelatin (0.2%). Degradation of gelatin /proteolytic activity results in an increase of fluorescence. Data represent mean ± SEM. MCF-7-EMT n = 3, MDA-MB-231 n = 3 using unpaired, two-tailed t-test analysis to respective control. *P < *0.05* (**H**) Assessment of proteolytic activity of MCF-7 breast cancer cells after treatment with rhCTGF. Data represent mean ± SEM. M n = 3 using unpaired, two-tailed t-test analysis to respective control (untreated).*P < *0.05.* Scale bar gauges 200 µm.

Cell invasion as initial step of metastatic cascade results from suppression of cell–cell adhesion modulated by cadherin’s and cell-ECM adhesion promoted through different receptors including integrins^25^. Secretome- and proteome analysis elucidated that co-culturing non-invasive MCF-7 breast cancer cells with osteosarcoma cells led to an expression alteration of proteins involved in cell adhesion. We tested if cell-ECM adhesion was altered in invasive breast cancer cells (MCF-7-EMT, MDA-MB-231) when intracellular CTGF was suppressed by RNA interference, extracellular CTGF was blocked using CTGF-specific antibodies or non-invasive MCF-7 breast cancer cells were treated with rhCTGF. CTGF suppression increased cell-ECM adhesion (Fig. 4A, B; MCF-7-EMT: 146.3 ± 12.1% vs. control; *P* = 0.0185; n = 3; MDA-MB-231: 168.3 ± 14.3% vs. control; *P* = 0.0083; n = 3). Blocking extracellular CTGF increased cell-ECM adhesion (Fig. 4C, D; MCF-7-EMT: 120.6 ± 5.724% vs. IgG control; *P* = 0.0071; n = 5; MDA-MB-231: 110.5 ± 3.776% vs. IgG control; *P* = 0.0493; n = 3). Adding rhCTGF to non-invasive MCF-7 breast cancer cells resulted in dose-dependent decreased cell-ECM adhesion (Fig. 4E, F; MCF-7 1 µg/ml rhCTGF: 94.2 ± 5.809% mean difference vs. untreated; *P* = 0.0459; F = 6.244; n = 3).

Matrix metalloproteinases contribute to invadopodia formation and tissue invasion through proteolytic activity alteration of cells^26^. We examined, whether suppression of CTGF or treatment with rhCTGF regulates relative proteolytic activity of breast cancer cells. Reduced CTGF expression decreased relative proteolytic activity of mesenchymal transformed breast cancer cells (Fig. 4G; MCF-7-EMT CTGF^-^: 117 ± 28.92% vs. control; *P* = 0.0205; n = 3), while it did not alter relative proteolytic activity of TNBC cells (Fig. 4G). Treatment with rhCTGF induced proteolytic activity in non-invasive MCF-7 breast cancer cells (Fig. 4H; MCF-7 rhCTGF: 113.7 ± 4.229% vs. untreated; *P* = 0.0314; n = 3).

### CTGF differentially regulates potential drivers of invasion and EMT-markers in mesenchymal transformed and triple negative breast cancer cells

**Figure 5:**
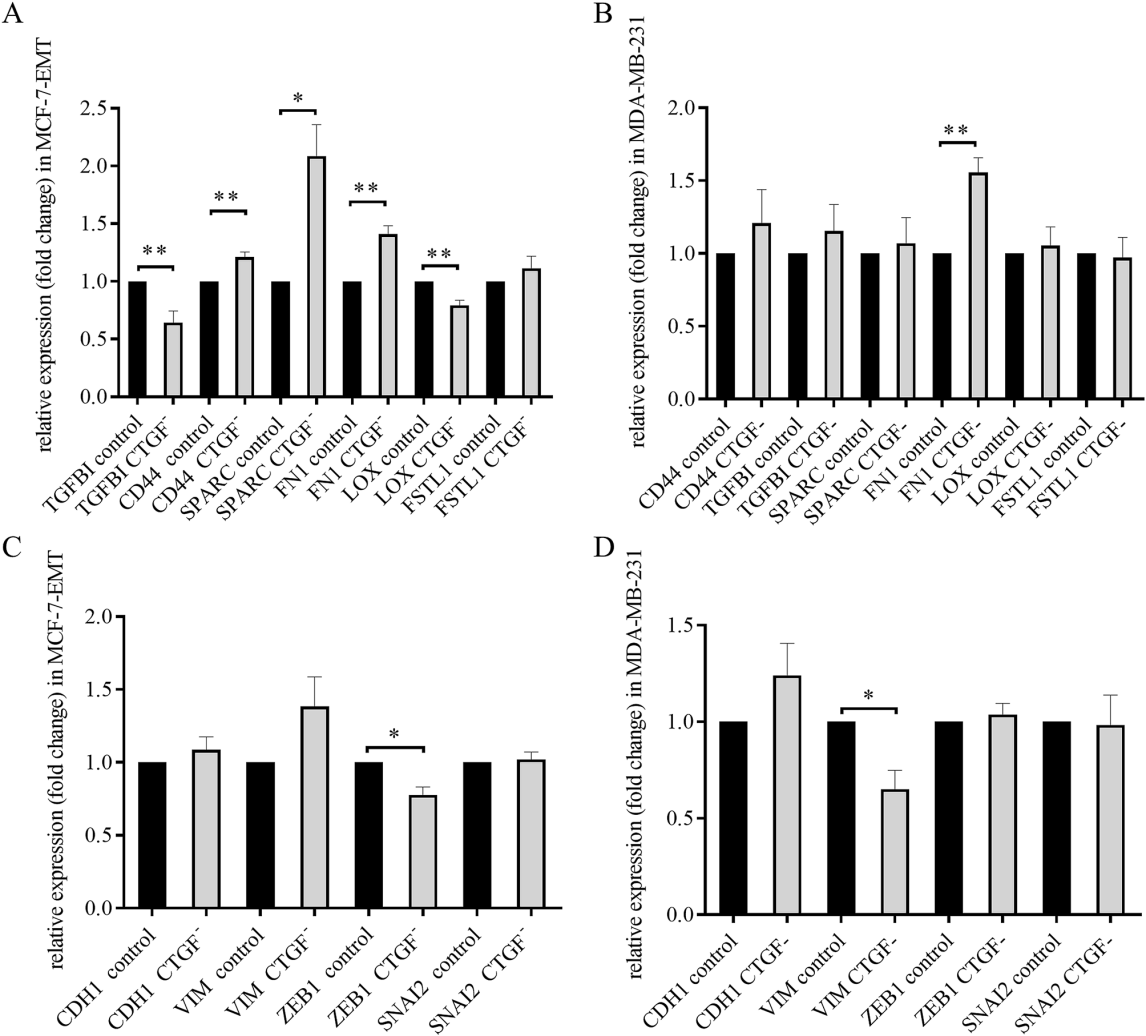
CTGF regulates expression of potential drivers of invasion and EMT-markers. (**A**) Relative quantification of TGFBI, CD44, SPARC, FN1, LOX and FSTL1 mRNA expression in mesenchymal transformed breast cancer cells treated transiently with CTGF siRNA for 48 h. Data represent mean ± SEM. MCF-7-EMT n = 3 using unpaired, two-tailed t-test analysis to respective control. *P < 0.05; **P < *0.01* (**B**) Relative quantification of TGFBI, CD44, SPARC, FN1, LOX and FSTL1 mRNA expression in triple negative breast cancer cells treated transiently with CTGF siRNA for 48 h. Data represent mean ± SEM. MDA-MB-231 n = 3 using unpaired, two-tailed t-test analysis to respective control. *P < *0.05* (**C**) Relative quantification of EMT markers VIM, CDH1, SNAI2 and ZEB1 mRNA expression in mesenchymal transformed breast cancer cells treated transiently with CTGF siRNA for 48 h. Data represent mean ± SEM. MCF-7-EMT n = 3 using unpaired, two-tailed t-test analysis to respective control. *P < *0.05* (**D**) Relative quantification of EMT markers VIM, CDH1, SNAI2 and ZEB1 mRNA expression in triple negative breast cancer cells treated transiently with CTGF siRNA for 48 h. Data represent mean ± SEM. MDA-MB-231 n = 3 using unpaired, two-tailed t-test analysis to respective control. *P < *0.05.*

To further analyze underlying mechanisms of CTGF-induced invasion and suppressed adhesion we examined, if reduced CTGF expression alters expression of TGFBI, CD44, SPARC, FN1, LOX and FSTL1 which were all identified potential drivers for invasion by secretome analysis. We could detect, that reduced CTGF in mesenchymal transformed breast cancer cells suppressed expression of TGFBI (Fig. 5A; TGFBI CTGF^-^: 0.6474 ± 0.1107 FC vs. TGFBI control; *P* = 0.0052; n = 6) and LOX (Fig. 5A; LOX CTGF^-^: 0.7933 ± 0.043 FC vs. LOX control; *P* = 0.0088; n = 3), and increased expression of CD44 (Fig. 5A; CD44 CTGF^-^:1.21 ± 0.045 FC vs. CD44 control; *P* = 0.0096; n = 3), SPARC (Fig. 5A; SPARC CTGF^-^: 2.083 ± 0.2749 FC vs. SPARC control; *P* = 0.0169; n = 3) and FN1 (Fig. 5A; FN1 CTGF^-^: 1.41 ± 0.07234 FC vs. FN1 control; *P* = 0.0048; n = 3). Suppressed CTGF expression altered FN1 (Fig. 5B; FN1 CTGF^-^: 1.557 ± 0.1014 FC vs. FN1 control; *P* = 0.0054; n = 3) expression in TNBC cells.

We found that CTGF had in impact on TGFBI-expression, and further wanted to test, whether a reduced CTGF expression can regulate expression of EMT transcription factors. We examined expression of Cadherin 1 (CDH1), Vimentin (Vim), ZEB1 and SNAIl family transcriptional repressor 2 (SNAI2) after transient CTGF suppression in mesenchymal transformed and TNBC cells. We found that downregulation of CTGF led to reduced ZEB1 expression in mesenchymal transformed breast cancer cells (Fig. 5C; 0.7767 ± 0.063 FC vs. control; *P* = 0.0138; n = 3). In contrast, suppressed CTGF resulted in downregulated Vimentin expression in TNBC cells (Fig. 5D; 0.65 ± 0.0985; *P* = 0.0237; n = 3).

**Figure 6:**
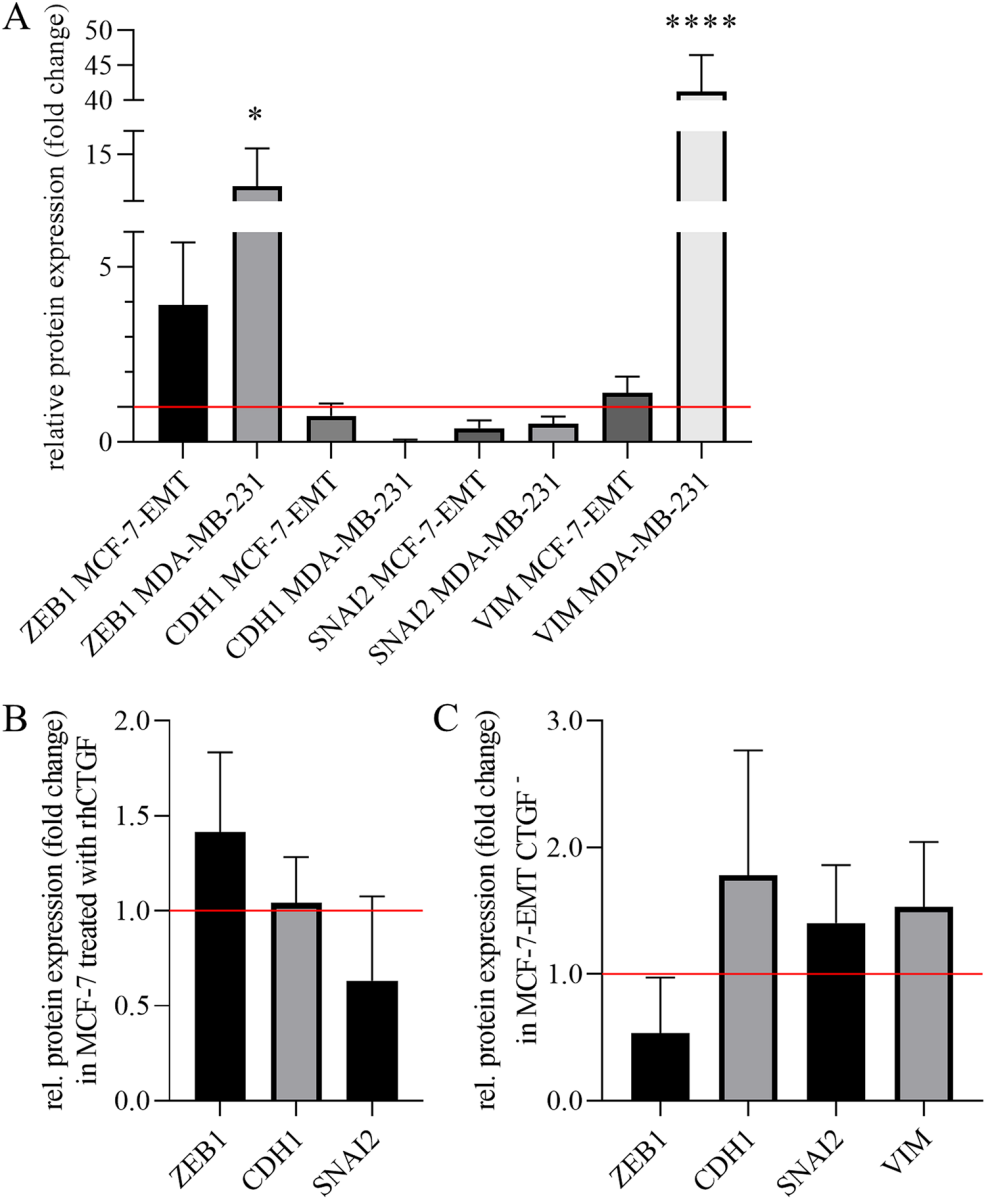
CTGF regulates protein expression of potential drivers of invasion and EMT-markers. (**A**) Relative quantification of ZEB1, CDH1, SNAI2 and VIM protein expression in mesenchymal transformed MCF-7-EMT and MDA-MB-231 breast cancer cells in comparison to MCF-7 breast cancer cells. Data represent mean ± SEM. n = 3 using one-way ANOVA with F = 39.31 and a Dunnett ‘s multiple comparison test with no matching or pairing between groups. *P < 0.05; ****P < *0.0001* (**B**) Relative quantification of ZEB1, CDH1 and SNAI2 protein expression in MCF-7 cells treated with rhCTGF. Data represent mean ± SEM. n = 3 using one-way ANOVA with F = 3.123 and a Dunnett ‘s multiple comparison test with no matching or pairing between groups. (**C**) Relative quantification of ZEB1, CDH1, SNAI2 and VIM protein expression in mesenchymal transformed breast cancer cells (MCF-7-EMT) treated transiently with CTGF siRNA for 48 h. Data represent mean ± SEM. n = 3 using one-way ANOVA with F = 2.191 and a Dunnett ‘s multiple comparison test with no matching or pairing between groups.

In a next step we compared protein expression of ZEB1, Cadherin 1 (CDH1), SNAIl family transcriptional repressor 2 (SNAI2) and Vimentin (Vim) in mesenchymal transformed MCF-7-EMT and MDA-MB-231 cells with their protein expression in MCF-7 cells (Fig.6A and supplementary Fig. 3). We found that protein expression of ZEB1 and Vimentin (Vim) in MCF-7-EMT and MDA-MB-231 cells was increased in comparison to MCF-7 cells, whereas protein expression of Cadherin 1 (CDH1) and SNAIl family transcriptional repressor 2 (SNAI2) was reduced (Fig. 6A; ZEB1 MCF-7-EMT: 3.93 ± 1.78 vs. ZEB1 MCF-7; ZEB1 MDA-MB-231: 12.27 ± 3.28 vs. ZEB1 MCF-7; CDH1 MCF-7-EMT: 0.75 ± 0.35 vs. CDH1 MCF-7; CDH1 MDA-MB-231: 0.04 ± 0.04 vs. CDH1 MCF-7; SNAI2 MCF-7-EMT: 0.40 ± 0.23 vs. SNAI2 MCF-7; SNAI2 MDA-MB-231: 0.52 ± 0.21 vs. SNAI2 MCF-7; VIM MCF-7-EMT: 1.14 ± 0.47 VIM MCF-7; VIM MDA-MB-231: 41.28 ± 5.21 vs. VIM MCF-7). When MCF-7 cells were treated with rhCTGF, expression of ZEB1 increased while SNAIl family transcriptional repressor 2 (SNAI2) was reduced (Fig. 6B; ZEB1: 1.41 ± 0.24 vs. untreated control; CDH1: 1.04 ± 0.14 vs. untreated control; SNAI2 0.63 ± 0.20 vs. untreated control). There was no change in Cadherin 1 (CDH1). Expression of Vimentin (Vim) was not detectable (not shown). Transient suppression of CTGF expression led to a reduced expression of ZEB1 and to an increased expression of Cadherin 1 (CDH1), SNAIl family transcriptional repressor 2 (SNAI2) and Vimentin (Vim) (Fig. 6C; ZEB1: 0.53 ± 0.25 vs. untreated control; CDH1: 1.78 ± 0.57 vs. untreated control; SNAI2: 1.40 ± 0.26 vs. untreated control; VIM: 1.53 vs. untreated control).

### GnRH agonist regulates CTGF expression through altered RhoA activity in mesenchymal transformed breast cancer cells

**Figure 7:**
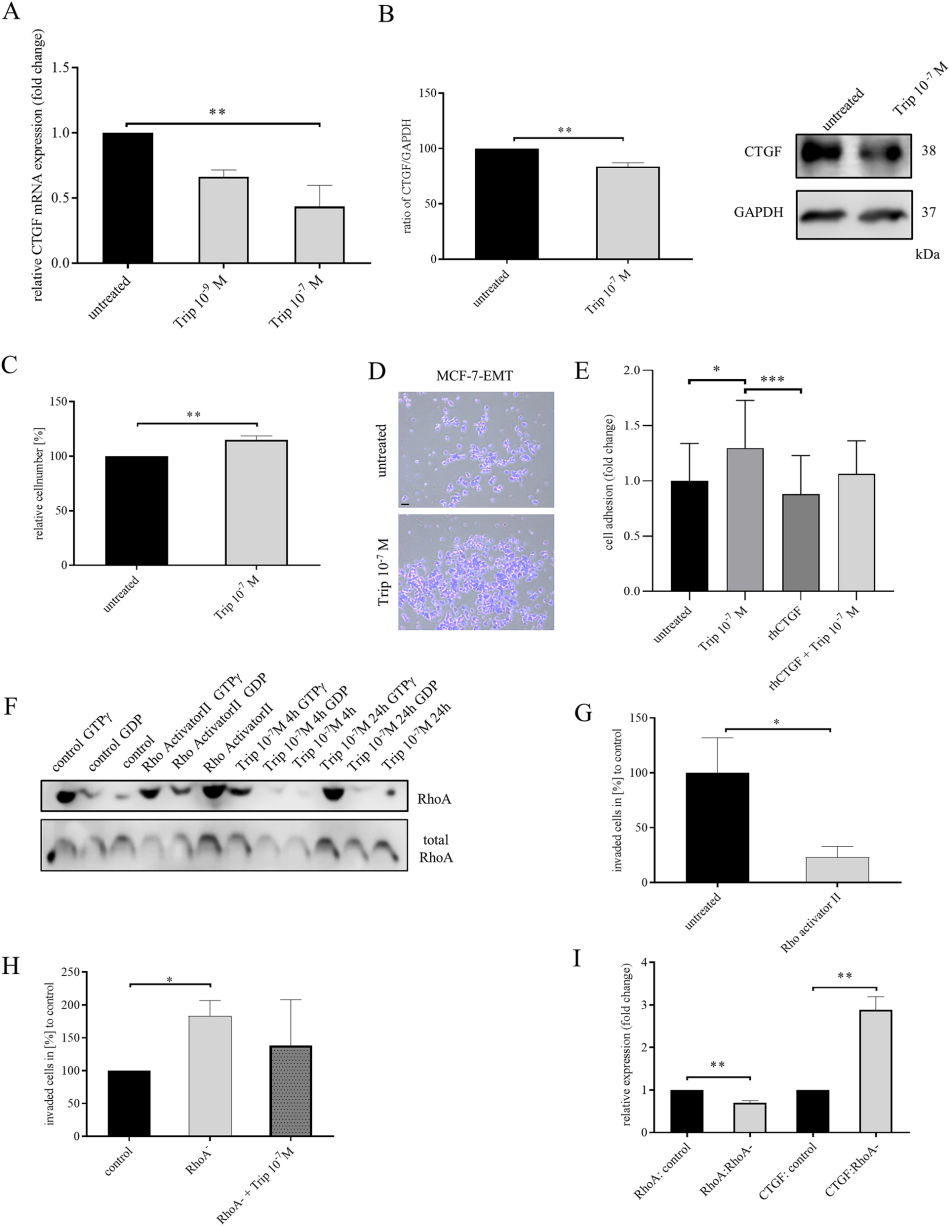
GnRH agonist regulates CTGF through RhoA activity in mesenchymal transformed breast cancer cells. (**A**) Relative quantification of CTGF mRNA expression in mesenchymal transformed breast cancer cells (MCF-7-EMT) treated for 48 h with 10^-9^ M or 10^-7^ M Triptorelin. Data represent mean ± SEM. MCF-7-EMT n = 3 using one-way ANOVA with F = 8.366 and a Dunnett ‘s multiple comparison test with no matching or pairing between groups. **P < *0.01* (**B**) Quantification and representative experiment of CTGF protein expression after Triptorelin treatment for 48 h (10^-7^ M). CTGF band intensity was quantified by densitometry and normalized to GAPDH. Lower panel shows loading control GAPDH that was detected in the same sample and were run in the same gel lane and detected in the same Western blot membrane. Data represent mean ± SEM. MCF-7-EMT n = 3 using unpaired, two-tailed t-test analysis to respective control (untreated). **P < *0.01* (**C**) Adhesion analysis of mesenchymal transformed breast cancer cells treated with 10^-7^ M Triptorelin. Adhesive cells where counter-stained with crystal violet and absorption was measured at 570 nm. Data represent mean ± SEM. MCF-7-EMT n = 5 using unpaired, two-tailed t-test analysis to respective control (untreated). **P < *0.01* (**D**) Representative images corresponding to C. Scale bar gauges 200 µm. (**E**) Adhesion analysis of mesenchymal transformed breast cancer cells treated with 10^-7^ M Triptorelin, rhCTG or rhCTGF and Triptorelin. Data represent mean ± SEM. n = 24 using one-way ANOVA with F = 5.784 and a Tukey ‘s multiple comparison test with no matching or pairing between groups. *P < 0.05; ***P < *0.001* (**F**) RhoA activity pull-down of untreated MCF-7-EMT cells, MCF-7-EMT cells treated 3 h with an specific Rho activator (1 µg/ml) and MCF-7-EMT cells treated with 10^-7^ M Triptorelin for 4 or 24 h. Lower panel shows total RhoA that was detected in the same sample and were run in the same gel lane and detected in the same Western blot membrane. (**G**) 2D invasion assay. After 48 h treatment with or without Rho activator II treatment (1 µg/ml) supplement invaded cells under filter were counted in four randomly selected regions. Data represent mean ± SEM. MCF-7-EMT n = 7 using unpaired, two-tailed t-test analysis to respective control (untreated). *P < *0.05* (**H**) Following transient RhoA siRNA transfection without or with treatment with 10^-7^ M Triptorelin invaded cells under filter was counted in four randomly selected regions. Data represent mean ± SEM. n = 9 using one-way ANOVA with F = 4.780 and a Tukey ‘s multiple comparison test with no matching or pairing between groups. *P < *0.05* (**I**) Relative quantification of RhoA and CTGF mRNA expression in MCF-7 cells after transient RhoA transfection (t0h). Data represent mean ± SEM. M n = 3 using unpaired, two-tailed t-test analysis to respective control. **P < *0.01.*

Most luminal breast cancer will metastasize to bone^27^. Suppression of ovarian function is part of therapy of endocrine-sensitive premenopausal early and advanced hormone receptor-positive breast cancer. Triptorelin, a GnRH agonist, revealed clinical benefit in high-risk patients by suppressing ovarian steroids and it has been investigated in attempt to preserve ovarian function during chemotherapy in young female patients^28^. GnRH receptor is expressed in 50–64% of all human breast cancers^29–33^. Around 15% of all human breast cancers are stated as TNBC, which is associated with high risk recurrence and metastasis^34,35^. Approximately 74% of all TNBC express GnRH receptor^13,36,37^. It was observed that GnRH agonist Triptorelin has impact on breast cancer invasiveness^13,14,38^. Accordingly, we wanted to assess, whether Triptorelin treatment suppresses CTGF expression. Mesenchymal transformed breast cancer cells were treated for 48 h with 10^–9^ M or 10^–7^ M Triptorelin every 24 h. We found that treatment with 10^–7^ M Triptorelin reduced CTGF expression (Fig. 7A; Triptorelin 10^-7^ M: 0.435 ± 0.565 FC vs. untreated; *P* = 0.0052; F = 8.366; n = 3; and Fig.7B; 83.67 ± 3.383% vs. untreated control; *P* = 0.0085; n = 3) which we could verify in TNBC cell as well (supplementary Fig. 6A,B). Furthermore, we analyzed, whether Triptorelin treatment altered cell-ECM adhesion. We found that 10^–7^ M Triptorelin treatment increased cell-ECM adhesion (Fig. 7C, D; Triptorelin 10^-7^ M: 114.9 ± 3.861% vs untreated; *P* = 0.0049; n = 5), which we found to be true for TNBC cells as well (supplementary Fig. 6C, D). Treatment with Triptorelin significantly increased cell adhesion (Fig. 7E; 1.30 ± 0.09 vs. untreated control; *P* = *0.0249*), while treatment with rhCTGF slightly decreased adhesion (Fig. 7E; 0.88 ± 0.07; not significant vs. control; *P* = *0.0006* vs. Trip 10^–7^ M; n = 24). In combination with rhCTGF, treatment with Triptorelin led to a reduced increase of adhesion (Fig. 7E; 1.06 ± 0.06; n = 24) indicating that rhCTGF could reverse the effect of Triptorelin.

It was suggested earlier that RhoA determines mesenchymal cell fate and regulates CTGF cleavage^39^. We wanted to test, if GnRH agonist Triptorelin facilitates reduced invasiveness and increased adhesion by regulating RhoA activity. We found that Triptorelin regulates RhoA activity in a time-dependent manner. After 4 h Triptorelin treatment (10^-7^ M) no increased RhoA activity could be detected by active RhoA pulldown. After 24 h a clear increased RhoA activity appeared (Fig. 7F). Furthermore, we found that mesenchymal transformed breast cancer cells treated with a Rho activator exhibit a decreased invasive capacity (Fig. 7G; RhoA activator II: 22.99 ± 9.922% vs. untreated; *P* = *0.0401*; n = 7), which could be verified for TNBC cells as well (supplementary Fig. 6E). Besides, non-invasive MCF-7 breast cancer cells with transiently suppressed RhoA expression exhibit an increased invasiveness (Fig. 7H; RhoA^-^: 183.1 ± 23.45% vs. control; *P* < 0.05; n = 9). This increased invasiveness was partially reduced by treatment with Triptorelin (Fig. 7H; RhoA^-^ + Trip 10^-7^ M: 138.48 ± 23.23 vs. control; not significant; n = 9). Furthermore, we tested if this increased invasiveness is due to an increased CTGF expression. We could observe that through reduction of RhoA expression (verification; supplementary Fig. 7A and Fig. 7I; RhoA: RhoA^-^: 0.7033 ± 0.04702 FC vs. control; *P* = 0.0032; n = 3) CTGF expression is increased (Fig. 7I; CTGF RhoA^-^: 2.88 ± 0.3143 FC vs. control; *P* = 0.0039; n = 3), while proliferation was not altered (supplementary Fig. 7B).

### Discussion

Tumor metastasis is highly regulated by micro environmental changes. Drugs are needed to modify breast micro environment were tumor cells gain ability to disseminate and bone micro environment, which is the niche where breast cancer cells preferentially colonize and remain in a state of survival and dormancy. Micro environmental modifications may be lethal for isolated, dormant cancer cells, reducing risk of reactivating dormant cells and growth of distant metastases over time is a high priority in preventing metastasis. Here we suggest potential drivers of initial dissemination of tumor cells with regards to bone-directed metastasis.

An increased CTGF expression in human breast cancer correlates with poor patient outcome and drug resistance^40^. It was suggested previously that downregulation of CTGF inhibits bone metastasis in a BMP9-dependent manner^41^. Two major questions have remained: will targeting CTGF help to prevent breast cancer cell dissemination into surrounding tissue, which underlying molecular mechanisms are involved in breast cancer directed bone metastasis.

We found that CTGF is highly upregulated in invasive ductal carcinoma and during co-culture of breast cancer cells with osteosarcoma cells. Furthermore, CTGF expression is comparable in bone and mammary gland tissue.

Consistent with recent findings we could assess that an elevated expression of CTGF led to increased cell invasiveness and correlated with bone-directed metastasis. Reducing CTGF expression resulted in a decreased invasion in 2D and 3D invasion assays. It was suggested earlier, that FN1 has a protective function against metastasis when uncleaved^24^ and that autocrine FN1 inhibits breast cancer metastasis^42^. Additionally, it was proposed that CTGF is cleaved by MMPs to reactivate angiogenesis^22^. Expression of MMP2 was upregulated when MCF-7 cells were co-cultured with osteosarcoma cells. We tested if 3D MCF-7 spheroid area growth can be altered when CTGF and/or FN1 and/or MMP2 and /or a MMP inhibitor are added. Interestingly, we found that spheroid area growth was significantly increased when CTGF or MMP2, CTGF and MMP2, and CTGF with FN1 and MMP2 are added. But there was no increased area growth when FN1 or FN1 with CTGF neither FN1 with MMP2 was added. Therefore we could assess that FN1 does not alter invasive behavior of breast cancer cells in 3D invasion setup. Also, treatment with CTGF and FN1 or MMP2 with FN1 did not alter invasive behavior as well, which could be an indicator for a protective FN1 feature. Only treatment with CTGF, MMP2 and FN1 led to an increased area growth. This effect could be reversed by an additional treatment with an MMP inhibitor (BB-94). But surprisingly this inhibitor was not effective enough to reverse effect of CTGF treatment, which could be an indicator for a MMP2-independent mechanism.

Loss of intercellular and cell-ECM adhesion allows malignant cells to escape from their site of origin^43^. To further analyze, why cancer cells treated with extracellular CTGF are highly invasive, we analyzed their cell-ECM adhesive and proteolytic abilities. We suggest that reduced CTGF increases cell-ECM adhesion, while ECM degradation was decreased. Increased extracellular CTGF expression led to decreased cell-ECM adhesion and increased ECM degradation. This is supported by previous findings that CTGF induces expression of ECM degradations genes and fibronectin^44^.

MCF-7-EMT cells exhibited increased expression of TGFBI, Twist, Vimentin and N-cadherin, while E-cadherin expression was reduced. Also MCF-7-EMT cells are more invasive^14^. We could furthermore identify, that these mesenchymal transformed breast cancer cells revealed a high ITGαV (CD51) and VCAM-1 (CD106) co-expression compared to non- invasive MCF-7 breast cancer cells. Interestingly, it was suggested, that CTGF stimulates osteosarcoma metastasis by upregulating VCAM-1 expression. Additionally, VCAM-1 may have a role in activation of dormant micro metastasis^9,45,46^. CTGF enhances cell motility in breast cancer through integrinαVβ3-ERK1/2 dependent S100A4 upregulation^47^. We analyzed impact of CTGF on other secretome analysis detected targets and could detect that reducing CTGF expression represses TGFBI, LOX and ZEB1 expression in mesenchymal transformed breast cancer cells. LOX was demonstrated to be involved in collagen I stabilization leading to chemo resistance^48^. It was proposed previously that EMT-TFs SNAI1 and SNAI2 activate TGBFI signaling in breast cancer and that CTGF and SPARC are upregulated as well^49^. Reduced CTGF expression led to increased CD44, SPARC, and FN1 expression in mesenchymal transformed breast cancer cells. CD44 is a stem cell marker and appears to have a dual nature regarding tumor progression and metastasis^50^. SPARC has anticancer effects^51^, inhibits bone metastasis^52^, and was suggested to be involved in same biological pathways than CTGF^53^. We could assess earlier in that study that an increased FN1 expression prevents 3D invasion, even when CTGF is added as well. This could indicate that downregulation of CTGF leads to an increased FN1 expression. We found that suppressed CTGF upregulated FN1 in TNBC cells, and downregulated Vimentin. Except for similar CTGF-dependent FN1 regulation, regulated targets are cell-type specific and could be related to expression of hormone- receptors or to MDA-MB-231 cell line specific mutations. These interesting observations need further evaluation by analyzing CTGF driven mechanism in another TNBC cell line and a hormone receptor positive mesenchymal transformed cell line.

Discovering the prominent role of CTGF during breast cancer invasion by modifying cell adhesion, ECM degradation and FN1 expression, we wanted to test if CTGF can be targeted and elucidated molecular mechanism by which CTGF can be repressed to suppress cell dissemination and colonization at distant sites. We found that GnRH agonist Triptorelin, which is in clinical use for ovarian function suppression of premenopausal breast cancer with high clinical risk of recurrence^28^, and was demonstrated to reduce breast cancer invasion^37^, reduced CTGF expression in mesenchymal transformed breast cancer in a dose-dependent manner. Furthermore, we found that CTGF was downregulated by Triptorelin treatment in TNBC cells. GnRH receptor is expressed in 50–60% of all human breast cancer and to a further extent in approximately 74% of all TNBC^13,36,37^. We could demonstrate that treatment with 10^–7^ M Triptorelin led to an increased cell-ECM adhesion in mesenchymal transformed breast cancer cells and TNBC cells as it was detected by CTGF suppression as well.

It was suggested that RhoA determines lineage fate of mesenchymal stem cells in ECM and that RhoA activity controls CTGF cleavage^39^. Beside, Arguilar-Rojas and colleagues found out that Busrelin, a GnRH agonist, regulates RhoA activity in MDA-MB-231 breast cancer cells thereby decreasing invasiveness^54^. We wanted to examine, if Triptorelin regulates RhoA activity and also if RhoA expression has an impact on CTGF expression. We could observe that Triptorelin induces RhoA activity in a time-dependent manner in mesenchymal transformed breast cancer cells. As expected, invasiveness of mesenchymal transformed breast cancer cells was reduced when RhoA was activated. Later we wanted to assess if reducing RhoA expression has an impact on invasiveness of non-invasive MCF-7 breast cancer. We found that transient RhoA suppression led to increased invasion, which is facilitated through upregulation of CTGF. The increased invasiveness was partially reduced by treatment with Triptorelin, indicating a specific regulation of RhoA by Triptorelin. In addition, CTGF treatment reduced the effect of Triptorelin, indicating a specific regulation of CTGF by Triptorelin. This led to the conclusion that CTGF expression is dynamically regulated through RhoA activation and thereby regulates cell-ECM adhesion.

On molecular level it would be interesting to evaluate, if Triptorelin treatment has an impact on cell plasticity by regulating EMT-TF expression. CTGF activates ERK1/2 signaling through ITGαV cascade^47^ and plastic breast cancer cell co-express higher ITGαV and VCAM-1 receptors and exhibit an increased CTGF expression. ERK1/2 appears to be a new treatment option with promising preclinical phase I trials^55,56^. Targeting CTGF when cancer cells gained drug resistance, could help to identify new treatment options. In addition, a new phase III trial study (HOrmonal BOne Effects-2, HOBOE-2) revealed interesting results using zoledronic acid which is approved to treat osteoporosis^57^. In this context it may be worthwhile to examine if zoledronic acid reduces extracellular CTGF, which may open up possibilities for preventing bone metastasis.

Using proteome analysis it was detected, that heat shock proteins (HSP) are dysregulated when breast cancer cells are co-cultured with osteosarcoma cells (supplementary Fig. 2C, D and supplementary table 4). Nonetheless, further evaluation is necessary due to different basal expression of detected potential drivers within different cell lines. It was suggested previously, that cancer cells are more dependent on heat shock protein chaperonage due to an elevated level of misfolded onco-proteins^58,59^. Additionally, inhibiting HSP90 suppresses versatile pro-invasive and proangiogenic pathways^60^. Inhibiting HSP90 led to LATS1 and LATS2 depletion, which led to reduced YAP phosphorylation and decreased CTGF expression^61^. Targeting HSP90 could be of great interest to regulate CTGF expression and HSP90 inhibitors are currently under investigation for metastatic breast cancer^62–64^.

It should be noted that breast cancer tissue is a heterogeneous tissue with different cell types beside tumor cells, i.e. fibroblasts and stromal cells^65^. Increased expression of CTGF was found in the fibrous stroma of breast cancers^66,67^. And it has proposed that CTGF plays pivotal role in pathophysiology of many fibrotic disorders and is associated with TGFβ signaling^68^. We found that CTGF protein expression and secretion of MG-63 cells is highly increased as compared to MCF-7 breast cancer cells. It is therefore necessary to further investigate the influence that CTGF expression of fibroblasts and stromal cells has on initiation and progression of tumors in the breast.

**Figure 8:**
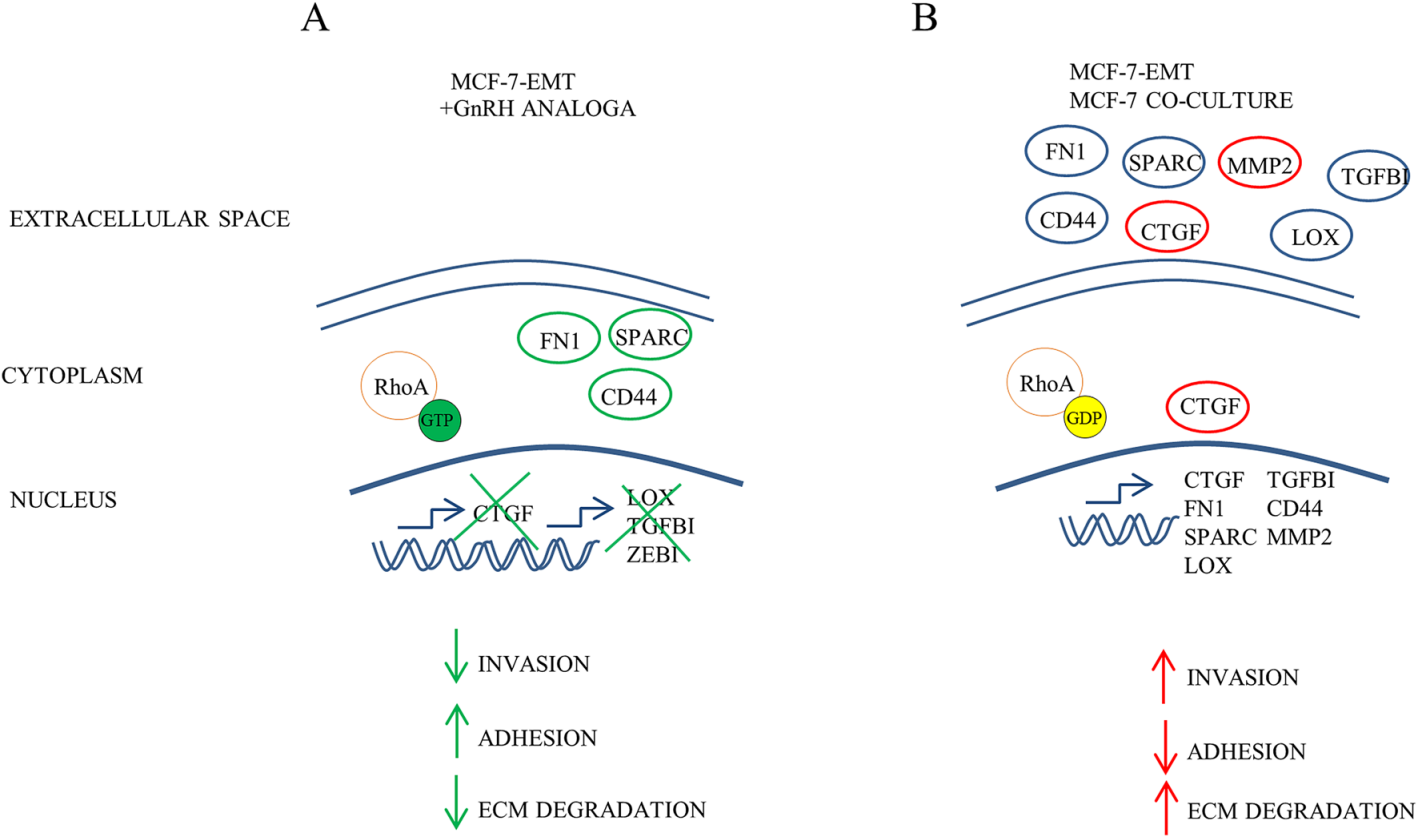
Proposed model of CTGF driven invasion in breast cancer. (**A**) Mesenchymal transformed breast cancer cells with Triptorelin treatment, CTGF blocking antibody or transiently suppressed CTGF expression reduced invasiveness, increased cell-ECM adhesion and reduced ECM degradation. (**B**) On the other hand, co-cultured non-invasive MCF-7 breast cancer cells or mesenchymal transformed breast cancer cells exhibit an increased CTGF expression, higher invasion, decreased cell-ECM adhesion and increased ECM degradation. The graphic was created using Microsoft PowerPoint 2016; www.microsoft.com.

Figure 8 shows our proposed model of CTGF driven invasion in breast cancer cells in vitro. Mesenchymal transformed breast cancer cells with GnRH agonist Triptorelin treatment, CTGF blocking antibody or transiently suppressed CTGF expression reduced invasiveness, increased cell-ECM adhesion and reduced ECM degradation (Fig. 8A). On the other hand, co-cultured non-invasive MCF-7 breast cancer cells or mesenchymal transformed breast cancer cells exhibit an increased CTGF expression, higher invasion, decreased cell-ECM adhesion and increased ECM degradation (Fig. 8B).

In summary, we identified a novel mechanism by which extracellular CTGF drives cell dissemination by regulating cell adhesion, ECM degradation, and regulation of EMT inducing factor TGFBI in vitro. Furthermore, we propose that CTGF is a versatile regulator in breast cancer and facilitates SPARC, LOX, ZEB1, VIM and FN1 expression changes. Moreover, it was assessed that CTGF expression is regulated by RhoA activity. Performed experiments support value of CTGF as therapeutic target for invasive breast cancer, and GnRH agonist Triptorelin could be of value in clinical applications. However, due to the high complexity of metastasis and diverse interactions between different cell types, it is necessary to confirm our findings in an animal model.

## Methods

### Cell culture

Human breast cancer cell lines MCF-7, MDA-MB-231 were obtained from the American Type Cell Collection (ATCC; Manassas, VA, USA) and cultured in minimum essential medium (MEM; biowest, Nuaillé, France) supplemented with 10% fetal bovine serum (FBS; biochrom, Berlin, Germany), 1% Penicillin/Streptomycin (P/S; Gibco, Carlsbad, CA, USA), 0,1% Transferrin (Sigma, St. Louis, USA) and 26 IU Insulin (Sanofi, Frankfurt, Germany). Human osteosarcoma cell line MG-63 was purchased from ATCC and cultured Dulbecco’s modified eagle medium (DMEM; Gibco) supplemented with 10% FBS (biochrom) and 1% Penicillin/Streptomycin (Gibco). To retain identity of cell lines, purchased cells were expanded and aliquots were frozen in liquid nitrogen. A new frozen stock was used every half year and mycoplasma testing of cultured cell lines was performed routinely using PCR Mycoplasma Test Kit I/C (PromoCell GmbH, Heidelberg, Germany). All cells were cultured in a humidified atmosphere with 5% CO_2_ at 37 °C.

### Generation of mesenchymal transformed MCF-7 cells

Mesenchymal transformed MCF-7 breast cancer cells (MCF-7-EMT) were generated as described earlier^14^. Briefly, 4 × 10^4^ cells/ml were cultured in prolonged mammosphere culture (5–6 weeks) in ultralow adherence six well plates (Corning, Lowell, MA, USA) in DMEM/F12 supplemented with 10% charcoal-stripped fetal calf serum (cs-FCS;PAN-biotech, Aidenbach, Germany), 2% B27 supplement (Invitrogen, Darmstadt, Germany), 1% penicillin/streptomycin, 0.5 mg/ml hydrocortisone (Sigma, St. Louis, MO, USA ), 5 µg/ml insulin, 20 ng/ml epidermal growth factor (EGF; Sigma, St. Louis, MO, USA).

### Small interfering RNA transfection

Breast cancer cell lines MCF-7-EMT (5 × 10^5^ cells/ml), MDA-MB-231 (2.5 × 10^5^ cells/ml) were seeded in 2 ml of MEM with 10% FBS (-P/S) in 25 cm^2^ cell culture flask. Cells were transiently transfected with siRNA specific to CTGF (sc-39329 pool of three specific siRNAs; Santa Cruz Biotechnology (SCBT), Dallas, USA) or RhoA (sc-44209 pool of three specific siRNAs; SCBT) in OPTI-MEM I medium (Gibco, Carlsbad, CA, USA) with siRNA transfection reagent (sc-29528; Santa Cruz Biotechnology, Dallas, USA). A non-targeting control was used as control (sc-37007 control-A; Santa Cruz Biotechnology, Dallas, TX, USA). After an incubation period of 6 h, MEM supplemented with 20% FBS and 20% penicillin/streptomycin was added.

### Transwell co-culture invasion assay

Using co-culture transwell assay as describes earlier^13^, 1 × 10^4^ breast cancer cells were seeded in DMEM w/o phenol red, supplemented with 10% cs-FCS into a cell cultural insert (upper well) with a polycarbonate membrane (8 µm pore diameter, Merck Millipore, Cork, Ireland) coated with 30µL of a Matrigel (BD Bioscience, Bedford, MA, USA) solution (1:2 in serum-free DMEM) or gelatin (1 mg/ml in PBS, Sigma). Osteosarcoma cells were seeded (2.5 × 10^4^) in DMEM supplemented with or without 10% cs-FCS into the lower well (24-well-plate). After 24 h cells were co-cultured for 96 h or 48 h when treated with RhoA activator II. Invaded cells on lower side of inserts were stained with hematoxylin and number of cells in four randomly selected fields of each insert was counted.

### 3D spheroid assay

Assessment of 3D cell invasion was pursued as describes earlier with minor changes^69^. Briefly 1 × 10^3^ breast cancer cells were seeded in 100 µL in a well of an ultra-low-adherence 96-well plate (ULA; Nexcelom, Cenibra GmbH, Bramsche, Germany). After 48 h spheroid formation was visually confirmed and 50 µL of media was removed. Thereafter, 50 µL Matrigel were added to wells with spheroids. Central position of spheroids was checked visually and Matrigel was allowed to solidify for 1 h at 37 °C and 5% CO^2^. Afterwards 50 µL media were added to each well and a picture was taken marking time point 0 (t0h). When indicated rhCTGF (recombinant human connective tissue growth factor; 1 µg/ml; R&D systems), pdhFN1 (plasma-derived human fibronectin 1; 1 µg/ml; R&D systems), rhMMP2 (recombinant human matrix metalloproteinase 2; 0.01 µg/ml; R&D systems, Minneapolis, MN, USA), Batimastat (BB-94, 4 nM; Selleckchem, Munich, Germany) polyclonal rabbit IgG control (15 µg/ml; R&D systems) or anti-CTGF (15 µg/ml; Novus Biologicals). Spheroid growth area was analyzed using ImageJ polygonal selection and measurement. Mean values were calculated and compared to respective control.

### Adherence assay

Cell-ECM adherence was examined by coating 96-well plates with bovine collagen I (30 µL; 0.04 mg/ml; BD Bioscience) for 12 h at 4 °C. Solution was aspirated and plate was left to dry under bench. Cells were washed 3 times with FBS-free DMEM and cultured for 8 h in DMEM-FBS prior to adhesion assessment. Cells were detached using 10 mM EDTA-PBS solution. Cells were pelleted (1300 rpm for 5 min) and washed twice with DMEM supplemented with 0.1% BSA. Cells were seeded (2 × 10^4^) in DMEM supplemented with 0.1% BSA, when indicated treated with rhCTGF (1 µg/ml; R&D systems), Triptorelin (10^–7^ M), polyclonal rabbit IgG control (15 µg/ml; R&D systems) or anti-CTGF (15 µg/ml; Novus Biologicals), and incubated at 37 °C with 5% CO_2_ for 20 min. Non-adherent cells were washed of by adding 100 µL of DMEM four times. Adherent cells were counter-stained with crystal violet solution (0.5%) for 20 min at RT shaking. Wells were washed four times with ddH_2_O and dried for at least 2 h. Pictures were taken and afterwards 200 µL Methanol were added to each well, incubated for 20 min shaking and absorbance was assessed at 570 nm using Synergy (BioTek Instruments, Bad Friedrichshall, Germany). Each experiment was performed in six replicates. Mean values were compared to respective control.

### ECM degradation

Degradation of ECM was examined by depriving cell from FBS 12 h prior to seeding. Wells of a 96-well plate were coated with 50 µL FITC-conjugated gelatin (2 mg/ml; BioVision Inc, Milpitas, CA,USA) diluted 1:5 with unlabeled gelatin (Sigma) and incubated for 1 h at 37 °C and 5% CO_2_. Solution was discarded and plate left to dry under bench. FBS deprived cells were seeded (1 × 10^4^) on gelatin coated wells and when indicated treated with rhCTGF (1 µg/ml; R&D systems). After 24 h proteolytic activity was detected by measuring fluorescence (extinction 490 nm/emission 520 nm) using Synergy (BioTek Instruments, Bad Friedrichshall, Germany). Each experiment was performed in three replicates. Mean values were compared to respective control.

### AlamarBlue assay

Transiently transfected breast cancer cells were seeded in 96- wells (1.25 × 10^3^) in DMEM w/o phenol-red supplemented with 10% cs-FBS and relative AlamarBlue reduction (BioRad, Hercules, USA) was assessed at 48 h and/or 120 h. 3D spheroids were grown as described above and 48 h after seeded in Matrigel AlamarBlue was added and incubated for 4 h. Thereafter, relative AlamarBlue reduction was measured by absorbance reading at 540 nm and 630 nm, using Synergy (BioTek Instruments). Relative AlamarBlue Reduction was calculated as indicated by manufacturer.

### Immunohistochemical staining

Immunohistochemical staining of human tissue array slides (BR248a; BRC961; BR20837; US Biomax, Derwood, MD, USA) was performed as described earlier^37^. Sections were deparaffinized and rehydrated. Thereafter, antigens were retrieved by slide incubation in 0.01 M citrate buffer (pH 6.0) in microwave (700 W) for 5 min. Using 3% hydrogen peroxidase solution for 6 min endogenous peroxidase activity was quenched. Sections were incubated over night with primary labeled antibodies against CTGF 1:1000 (#NB100-724, Novus Biologicals) in fluorescence staining solution (1% BSA + 0.4% TRITON X-100 in PBS) at 4 °C. Labeling was performed by incubating slide with secondary rabbit antibody Alexa488 (Invitrogen) and DAPI (1 µg/ml; Novus Biologicals) in fluorescence staining solution for 30 min at room temperature protected from light. Staining was visualized using a Zeiss Scope A1 Axio microscope (ZEISS, Oberkochen, Germany) with an oil EC PLAN-NEOFLUAR 100x (ZEISS, Oberkochen, Germany) objective and ZEN software (ZEISS, Oberkochen, Germany).

### Immunocytochemical staining

For immunocytochemical staining MCF-7, MCF-7-EMT, and MDA-MB-231 cells were grown on slides, fixed in 3.7% paraformaldehyde/PBS for 30 min and then rinsed twice with fluorescence staining solution (1% BSA + 0.4% TRITON X-100 in PBS) for 10 min. The fixed cells were incubated with primary labeled antibodies against vimentin 1:250 (VIM; #ab92547, Abcam), E-cadherin 1:500 (CDH1; #ab40772, Abcam), and CTGF 1:50 (#NB100-724, Novus Biologicals) in fluorescence staining solution at 4 °C for 1 h. Labeling was performed by incubating slide with secondary rabbit antibody Alexa488 (Invitrogen) and DAPI (1 µg/ml; Novus Biologicals) in fluorescence staining solution for 30 min at room temperature protected from light. After washing twice with fluorescence staining solution, slides were rinsed twice with DPBS. Staining was visualized as described above. Overlay images were made using ImageJ 1.52a software (NIH, Bethesda, MD, USA).

### Flow cytometry

Cells were detached from culture dish with trypsin for 5 min and washed once with PBS. 1 × 10^6^ were suspended in pre-cooled flow cytometry staining solution (PBS, 10% FCS, 1NaN_3_) and incubated with conjugated primary antibodies (CD51-FITC; CD-106-APC; eBioscience Inc., ThermoFisher Scientific, Waltham, MN, USA) for 20 min at 4 °C. Stained cells were washed twice with flow cytometry staining solution and analyzed immediately by BD CANTOII flow cytometer (BD Biosciences). Untreated cells and UltraComp compensations beads (Invitrogen) incubated with labeled antibodies were used as negative control for determining specificity of signal.

### Western Blot analysis

In Western Blot analysis, cells were lysed in lyse buffer consisting of cell lytic M buffer (Sigma, St. Louis, USA) supplemented with 0.1% phosphatase-inhibitor (Sigma, St. Louis, MO, USA) and 0.1% protease-inhibitor (Sigma, St. Louis, MO, USA ). Isolated proteins (40 µg) were fractioned using 12% SDS gels and electro-transferred to a polyvinylidene difluoride membrane (Merck Millipore, Cork, Ireland). Primary antibodies against CTGF 1:1000 (#NB100-724, Novus Biologicals), RhoA 1:500 (#ARH04, Cytoskeleton, Denver, CO, USA), E-cadherin 1:1000 (CDH1; #ab40772, Abcam, Cambridge, Great Britain), SNAI2 1:500 (#ab180714, Abcam), vimentin 1:1000 (VIM; #ab92547, Abcam), ZEB1 1:500 (#ab203829, Abcam), and GAPDH 1:2000 (#5174S, Cell Signaling, Danvers, MA, USA) were used. Membrane was washed and incubated in horseradish peroxidase-conjugated secondary antibody (GE Healthcare, Buckinghamshire, UK). Antibody-bond protein bands were assayed using a chemiluminescent luminol enhancer solution (Cyanagen, Bologna, Italy) and detected by a C-DiGit Blot Scanner (LI-COR Bioscience, Lincoln, NE, USA). Full-length Western blot images are shown in the supplement.

### RhoA pull-down

RhoA pulldown assay was examined using Rho activation assay biochem kit as described by the manufacturer (BK036-S; Cytoskeleton Inc.). Briefly, 300 µg proteins was loaded with 50 µg Rhotekin rho binding domain (RBD) glutathione agarose bound beads which binds/precipitates specifically active GTP-bond Rho proteins. To quantify active RhoA total RhoA protein was determined. A positive cellular control loaded with non-hydrolysable GTP analog (GTPγS) and a negative control loaded with GDP were determined from each examined sample. To assess functionality of assay one sample was treated with RhoA activator II (CN03, 1 µg/ml, Cytoskeleton). As quantitation estimate for endogenous Rho, His-RhoA protein was run on gel together with examined samples.

### Mass spectrometric secretome and proteome analysis

#### Sample preparation

Breast cancer cells were seeded (0.75 × 10^5^) in Bio-one ThinCert (Greiner Bio-one, Kremsmünster, Austria) and Osteosarcoma cells were seeded (1,3 × 10^5^) in 6 wells. After 24 h cells were deprived of FBS and co-cultured for 96 h. Cell medium was precipitated with acetone. Medium was centrifuged for 10 min at 13,300 rpm at 4 °C and five times volume on pre-cooled (-20 °C) was added to samples. Samples were vortexed and protein precipitation performed for 2 h at -20 °C. Protein was pelleted by centrifugation for 30 min at 13,300 rpm at 4 °C. Protein pellets were washed with ethanol (80%, pre-cooled at -20 °C), centrifuged for 30 min at 13,300 rpm at 4 °C, and protein pellets were air dried.

Cell lysates were generated by cutting membranes from insert and recovering cells with Recovery solution (Corning, New York, NY, USA) for 1 h at 4 °C while shaking. Cells were pelleted and resuspended in 30 µL lysis buffer.

#### MS sample processing

For generation of a peptide library, equal amount aliquots from comparable samples were pooled to a total amount of 100 µg, and separated into eight fractions using a reversed phase spin column (Pierce High pH Reversed-Phase Peptide Fractionation Kit, ThermoFisher Scientific). All samples were spiked with a synthetic peptide standard used for retention time alignment (iRT Standard, Schlieren, Schweiz).

Protein digests were analyzed on a nanoflow chromatography system (Eksigent nanoLC425) hyphenated to a hybrid triple quadrupole-TOF mass spectrometer (TripleTOF 5600 +) equipped with a Nanospray III ion source (Ionspray Voltage 2400 V, Interface Heater Temperature 150 °C, Sheath Gas Setting 12) and controlled by Analyst TF 1.7.1 software build 1163 (all AB Sciex). In brief, peptides were dissolved in loading buffer (2% acetonitrile, 0.1% formic acid in water) to a concentration of 0.42 µg/µl. For each analysis 2.1 µg of digested protein were enriched on a precolumn (0.18 mm ID × 20 mm, Symmetry C18, 5 µm, Waters, Milford/MA, U.S.A) and separated on an analytical RP-C18 column (0.075 mm ID × 250 mm, HSS T3, 1.8 µm, Waters) using a 90 min linear gradient of 5–35% acetonitrile/0.1% formic acid (v: v) at 300 nl min^-1^.

Qualitative LC/MS/MS analysis was performed using a Top25 data-dependent acquisition method with an MS survey scan of *m/z* 350–1250 accumulated for 350 ms at a resolution of 30,000 full width at half maximum (FWHM). MS/MS scans of *m/z* 180–1600 were accumulated for 100 ms at a resolution of 17,500 FWHM and a precursor isolation width of 0.7 FWHM, resulting in a total cycle time of 2.9 s. Precursors above a threshold MS intensity of 125 cps with charge states 2 + , 3 + , and 4 + were selected for MS/MS, the dynamic exclusion time was set to 30 s. MS/MS activation was achieved by CID using nitrogen as a collision gas and manufacturer’s default rolling collision energy settings. Three technical replicates per reversed phase fraction were analyzed to construct a spectral library.

For quantitative SWATH analysis, MS/MS data were acquired using 65 variable size windows^70^ across the 400–1,050 m*/z* range. Fragments were produced using rolling collision energy settings for charge state 2 + , and fragments acquired over an *m/z* range of 350–1400 for 40 ms per segment. Including a 100 ms survey scan this resulted in an overall cycle time of 2.75 s. Two replicate injections were acquired for each biological sample.

Protein identification was achieved using ProteinPilot Software version 5.0 build 4769 (AB Sciex) at “thorough” settings. MS/MS spectra from combined qualitative analyses were searched against UniProtKB human reference proteome (revision 04–2018, 93.661 entries) augmented with a set of 52 known common laboratory contaminants to identify 217 proteins at a False Discovery Rate (FDR) of 5% in the secretome, and 2,033 proteins at an FDR of 1% for whole proteome analysis. We consciously allowed for a larger FDR in the secretome analysis since identified candidate proteins were further validated during SWATH data extraction and by biochemical experimentation.

Spectral library generation and SWATH peak extraction were achieved in PeakView Software version 2.1 build 11,041 (AB Sciex) using SWATH quantitation microApp version 2.0 build 2003. Following retention time correction using iRT standard, peak areas were extracted using information from MS/MS library at an FDR of 1%^71^. Resulting peak areas were then summed to peptide and finally protein area values, which were used for further statistical analysis.

#### MS data availability

The mass spectrometry proteomics data have been deposited to the ProteomeXchange Consortium via the PRIDE^72^ partner repository with the dataset identifier PXD021539.

### Real-time quantitative PCR analysis

Total RNA was extracted using an RNeasy mini kit (Qiagen, Hilden, Germany) and 2 µg were reverse transcribed with high capacity cDNA reverse transcription kit (Qiagen, Hilden, Germany). Real- time qPCR was performed using SYBR green PCR master mix kit (Qiagen, Hilden, Germany) and following Primers: CTGF (forward) 5′- CTTGCGAAGCTGACCTGGAA-3′, CTGF (reverse) 5′- GTGCAGCCAGAAAGCTCAAA-3′, TGFBI (forward) 5′- AGGCCTTCGAGAAGATCCCT -3′, TGFBI (reverse) 5′- GAGATGATCGCCTTCCCGTT-3′, CD44 (forward) 5′- CACACCCTCCCCTCATTCAC-3′, CD44 (reverse) 5′- CAGCTGTCCCTGTTGTCGAA -3′, SPARC (forward) 5′- GTGCGAGCTGGATGAGAACA-3′, SPARC (reverse) 5′- TTGCAAGGCCCGATGTAGTC-3′, FN1 (forward) 5′- GCTGCACATGTCTTGGGAAC-3′, FN1 (reverse) 5′- CATGAAGCACTCAATTGGGCA-3′, LOX (forward) 5′- GGGCGACGACCCTTACAAC-3′, LOX (reverse) 5′- GCCCTGTATGCTGTACTGGC-3′, FSTL1 (forward) 5′- TCTGCCAGCCCAGTTGTTTG-3′, FSTL1 (reverse) 5′- GAGTCCAGGCGAGAATCACC-3′, CDH1 (forward) 5′-CCTCCTGAAAAGAGAGTGGA -3′, CDH1 (reverse) 5′- GTGTCCGGATTAATCTCCAG-3′, VIM (forward) 5′- GCTGCTAACTACCAAGACAC-3′, VIM (reverse) 5′-TCAGGTTCAGGGAGGAAAAG -3′, ZEB1 (forward) 5′- AAGACAAACTGCATATTGTGGAAG-3′, ZEB1 (reverse) 5′- CTGCTTCATCTGCCTGAGCTT-3′, SNAI2 (forward) 5′-GCCAAACTACAGCGAACTGG -3′, SNAI2 (reverse) 5′-GAGAGAGGCCATTGGGTAGC -3′, RhoA (forward) 5′-CAAGGACCAGTTCCCAGAGG -3′, RhoA (reverse) 5′-TGTCCCACAAAGCCAACTCT -3′, and GAPDH (forward) 5′- GAAGGTCGGAGTCAACGGAT -3′, GAPDH (reverse) 5′- TGGAATTTGCCATGGGTGGA -3′ .PCR conditions were: denaturing once at 95 °C (2 min), 95 °C (5 s), 60 °C (15 s) for 40 cycles.

### Data analysis

Gene ontology enrichment analysis, networks summarizing overlapping terms and hierarchical lustering trees were conducted using Shiny GO v0.60 with a p-value cutoff (FDR) of 0.05^73^.

### CTGF expression analysis in human tissues

Statistical analysis of tissue-specific CTGF expression was conducted using large public cancer genomics datasets (GTEX, TARGET, TCGA) as described previously^74^.

### Statistical analysis

All experiments were performed at least in three biological and technical replicates. Data were analyzed by GraphPad Prism Software version 8.41 (GraphPad Software Inc., La Jolla, CA/USA) using unpaired, two-tailed, parametric t-test comparing two groups (treatment to respective control) by assuming both populations have same standard derivation or ANOVA one-way analysis when more than two groups were compared. F-values were recorded and a Dunnett ‘s or a Tukey ‘s multiple comparison test with no matching or pairing between groups was calculated. *P* < 0.05 was considered statistically significant.

## Supplementary information

Supplementary file1

## Data Availability

Secretome and proteome data are available via ProteomeXchange with identifier PXD021539. The datasets used and/or analyzed during current study are available from the corresponding author on reasonable request.
