# Supplementary material for: Identification of drivers of breast cancer invasion by secretome analysis: insight into CTGF signaling
Source: Sci Rep. 2020 Oct 21;10:17889. doi: 10.1038/s41598-020-74838-8 (PMC7578015; doi:10.1038/s41598-020-74838-8)
Supplement: Supplementary file 1 — Supplementary file1 [file 41598_2020_74838_MOESM1_ESM.docx]

Supplementary Information

**Identification of breast cancer invasion drivers by secretome analysis: insight into CTGF signaling**

Johanna W. Hellinger^1^, Franziska Schömel^1^, Judith V. Buse^1^, Christof Lenz^2^, Gerd Bauerschmitz^1^, Günter Emons^1^, Carsten Gründker^1*^

**Affiliation**

^1^Department of Gynecology and Obstetrics, University Medical Center Göttingen, Göttingen, Germany

^2^Institute of Clinical Chemistry, University Medical Center Göttingen, Göttingen, Germany

***Corresponding Author:**

Carsten Gründker

Department of Gynecology and Obstetrics

University Medicine Göttingen

Robert-Koch-Str. 40

37075 Göttingen

Germany

Phone: (+49) 551 39 69810

Email: grundker@med.uni-goettingen.de

**Includes:**

Supplementary tables: 1 – 7

Supplementary figures: 1 – 7

Full-length gels / blots: figures 3b, 8b, 8f and supplemental figures 12b, 13b, 14a

Supplementary table 1: Protein findings from secretome analysis of breast cancer cells co-cultured with osteosarcoma cells. Information is given about gene symbol/User ID, Ensemble Gene ID, p-value and mean values of co-culture media and MCF-7 control media. List was used to further examine Gene Ontology (GO) enrichment using Shiny GO v06.0.

| **i** | **User ID** | **Ensemble Gene ID** | **p-value** | **mean co-culture** | **mean MCF-7 control** |
| --- | --- | --- | --- | --- | --- |
| 1 | HTRA1 | ENSG00000166033 | 0,00021228 | 28234 | 6597 |
| 2 | CD44 | ENSG00000026508 | 0,00037961 | 12615 | 1688 |
| 3 | C1R | ENSG00000159403 | 0,00033183 | 61150 | 8715 |
| 4 | POSTN | ENSG00000133110 | 0,0011454 | 95683 | 18550 |
| 5 | HEXA | ENSG00000213614 | 9,7674E-05 | 16849 | 2926 |
| 6 | B2M | ENSG00000166710 | 0,00135842 | 70650 | 3738 |
| 7 | LOXL1 | ENSG00000129038 | 3,8422E-10 | 65281 | 7724 |
| 8 | MMP2 | ENSG00000087245 | 0,00146649 | 4431667 | 96000 |
| 9 | COL1A1 | ENSG00000108821 | 0,0002255 | 3191667 | 78133 |
| 10 | NUCB1 | ENSG00000104805 | 0,00158982 | 120358 | 12667 |
| 11 | CNN2 | ENSG00000064666 | 7,0889E-06 | 81240 | 7659 |
| 12 | CAB39 | ENSG00000135932 | 0,00129469 | 45224 | 143832 |
| 13 | SERPINE2 | ENSG00000135919 | 9,9725E-05 | 7465 | 1199 |
| 14 | FN1 | ENSG00000115414 | 0,00127185 | 2013667 | 45350 |
| 15 | FSTL1 | ENSG00000163430 | 0,00158654 | 77483 | 11262 |
| 16 | IGFBP7 | ENSG00000163453 | 4,6509E-05 | 8350 | 1784 |
| 17 | TGFBI | ENSG00000120708 | 0,00065803 | 10008 | 1168 |
| 18 | SPARC | ENSG00000113140 | 0,00082713 | 2172000 | 116933 |
| 19 | LOX | ENSG00000113083 | 0,00111492 | 18118 | 5004 |
| 20 | COL23A1 | ENSG00000050767 | 0,00116807 | 23983 | 1270 |
| 21 | THBS2 | ENSG00000186340 | 0,00035021 | 59250 | 8683 |
| 22 | COL1A2 | ENSG00000164692 | 0,00108052 | 5150000 | 31383 |
| 23 | SERPINE1 | ENSG00000106366 | 0,00054046 | 37167 | 7040 |
| 24 | PCOLCE | ENSG00000106333 | 8,2456E-05 | 367167 | 34500 |
| 25 | CTSB | ENSG00000164733 | 0,00064317 | 827167 | 82000 |
| 26 | CLU | ENSG00000120885 | 0,00071045 | 130026 | 51465 |
| 27 | SVEP1 | ENSG00000165124 | 0,00046555 | 218000 | 29017 |
| 28 | TIMP1 | ENSG00000102265 | 0,00135049 | 168650 | 37333 |

Supplementary table 2: GO enrichment analysis of findings from secretome analysis. Protein discoveries listed in supplementary table 1 were examined for GO enrichment using Shiny GO v06.0. Information is given about enrichment FDR, how many genes within the discovery list are enriched within specific functional category, total number of genes within specific functional category, functional category and genes listed from discoveries which are enriched in specific category.

| **Enrichment FDR** | **Genes in list** | **Total genes** | **Functional Category** | **Genes** |
| --- | --- | --- | --- | --- |
| 3,26E-15 | 14 | 392 | Extracellular matrix organization | COL23A1, MMP2 , COL1A1 , TGFBI , POSTN ,  COL1A2 , LOX , CD44 , TIMP1 , SERPINE1 ,  SPARC , FN1 , LOXL1 , HTRA1 |
| 1,52E-14 | 14 | 460 | Extracellular structure organization | COL23A1, MMP2, COL1A1, TGFBI, POSTN,  COL1A2, LOX, CD44, TIMP1, SERPINE1, SPARC,  FN1, LOXL1, HTRA1 |
| 8,92E-09 | 11 | 585 | Wound healing | SERPINE1, SERPINE2, CNN2, TIMP1, COL1A1,  LOX, SPARC, FN1, POSTN, CD44, COL1A2 |
| 3,22E-08 | 11 | 687 | Blood vessel development | COL23A1, COL1A1, THBS2, SPARC, MMP2, LOX,  FN1, LOXL1, SERPINE1, TGFBI, COL1A2 |
| 3,22E-08 | 11 | 716 | Response to wounding | SERPINE1, SERPINE2, CNN2, TIMP1, COL1A1,  LOX, SPARC, FN1, POSTN, CD44, COL1A2 |
| 3,22E-08 | 11 | 715 | Vasculature development | COL23A1,COL1A1,THBS2,SPARC,MMP2,LOX,  FN1,LOXL1,SERPINE1,TGFBI,COL1A2 |
| 3,22E-08 | 18 | 2983 | Cellular response to organic substance | CD44, COL1A2, MMP2, PCOLCE, COL1A1, LOX,  SPARC, POSTN, IGFBP7, HTRA1, CTSB, SERPINE1,  CLU, CNN2, TIMP1, FN1, FSTL1, B2M |
| 3,22E-08 | 11 | 724 | Cardiovascular system development | COL23A1, COL1A1, THBS2, SPARC, MMP2, LOX,  FN1, LOXL1, SERPINE1, TGFBI, COL1A2 |
| 5,11E-08 | 19 | 3547 | Response to organic substance | TIMP1, CD44, CLU, COL1A2, MMP2, PCOLCE,  COL1A1, LOX, SPARC, LOXL1, POSTN, IGFBP7,  HTRA1, B2M, CTSB, SERPINE1, CNN2, FN1, FSTL1 |
| 3,11E-07 | 20 | 4507 | Response to stress | CD44, MMP2, SERPINE1, SERPINE2, CAB39, CLU,  CNN2, TIMP1, COL1A1, LOX, SPARC, FN1, POSTN,  C1R, FSTL1, IGFBP7, HTRA1, COL1A2, CTSB, B2M |
| 4,16E-07 | 18 | 3536 | Cellular response to chemical stimulus | CD44, POSTN, COL1A2, MMP2, PCOLCE, COL1A1,  LOX, SPARC, IGFBP7, HTRA1, B2M, CTSB, SERPINE1,  CLU, CNN2, TIMP1, FN1, FSTL1 |
| 1,31E-06 | 12 | 1372 | Response to cytokine | TIMP1, PCOLCE, COL1A1, LOX, SPARC, POSTN,  CD44, CNN2, MMP2, FN1, COL1A2, B2M |
| 1,31E-06 | 11 | 1077 | Circulatory system development | COL23A1, COL1A1, THBS2, SPARC, MMP2, LOX,  FN1, LOXL1, SERPINE1, TGFBI, COL1A2 |
| 2,76E-06 | 10 | 901 | Regulated exocytosis | CD44, CNN2, TIMP1, SERPINE1, SPARC, FN1,  CLU, CAB39, CTSB, B2M |
| 6,12E-06 | 15 | 2785 | Anatomical structure morphogenesis | COL23A1, THBS2, SERPINE1, SPARC, FN1, CLU,  MMP2, COL1A1, LOX, POSTN, SERPINE2, HTRA1,  TGFBI, CD44, COL1A2 |
| 6,12E-06 | 11 | 1278 | Cellular response to cytokine stimulus | PCOLCE, COL1A1, LOX, POSTN, CD44, CNN2, MMP2,  TIMP1, FN1, COL1A2, B2M |
| 7,03E-06 | 10 | 1023 | Exocytosis | CD44, CNN2, TIMP1, SERPINE1, SPARC, FN1,  CLU, CAB39, CTSB, B2M |
| 7,03E-06 | 8 | 541 | Skeletal system development | COL1A1, MMP2, TIMP1, LOX, SPARC, TGFBI,  CD44, COL1A2 |
| 9,69E-06 | 12 | 1704 | Response to endogenous stimulus | TIMP1, CD44, COL1A2, MMP2, COL1A1, LOX,  SPARC, POSTN, IGFBP7, HTRA1, CTSB, FSTL1 |
| 9,87E-06 | 12 | 1715 | Secretion by cell | FN1, POSTN, SERPINE2, CD44, CNN2, TIMP1,  SERPINE1, SPARC, CLU, CAB39, CTSB, B2M |
| 1,35E-05 | 8 | 603 | Blood vessel morphogenesis | COL23A1, THBS2, SPARC, MMP2, LOX, FN1,  SERPINE1, TGFBI |
| 1,39E-05 | 13 | 2168 | Tissue development | COL23A1, SERPINE1, COL1A1, FN1, TIMP1,  LOX, TGFBI, POSTN, SERPINE2, COL1A2, CTSB,  CD44, MMP2 |
| 1,63E-05 | 5 | 135 | Platelet degranulation | TIMP1, SERPINE1, SPARC, FN1, CLU |
| 1,94E-05 | 11 | 1506 | Cell migration | CD44, SERPINE1, COL1A1, SPARC, FN1, CNN2,  LOX, POSTN, TIMP1, SERPINE2, COL1A2 |
| 1,94E-05 | 12 | 1861 | Secretion | FN1, POSTN, SERPINE2, CD44, CNN2, TIMP1,  SERPINE1, SPARC, CLU, CAB39, CTSB, B2M |
| 3,13E-05 | 8 | 694 | Cellular response to growth factor stimulus | CD44, COL1A2, COL1A1, LOX, SPARC, POSTN,  HTRA1, FSTL1 |
| 4,09E-05 | 8 | 723 | Response to growth factor | CD44, COL1A2, COL1A1, LOX, SPARC, POSTN,  HTRA1, FSTL1 |
| 4,53E-05 | 7 | 511 | Angiogenesis | COL23A1, THBS2, SPARC, MMP2, FN1, SERPINE1,  TGFBI |
| 4,53E-05 | 11 | 1670 | Cell motility | CD44, SERPINE1, COL1A1, SPARC, FN1, CNN2,  LOX, POSTN, TIMP1, SERPINE2, COL1A2 |
| 4,53E-05 | 11 | 1670 | Localization of cell | CD44, SERPINE1, COL1A1, SPARC, FN1, CNN2,  LOX, POSTN, TIMP1, SERPINE2, COL1A2 |

Supplementary table 3 GO group enrichment analysis of findings from secretome analysis. Protein discoveries listed in supplementary table 1 were examined for GO group enrichment using Shiny GO v06.0.

| **N** | **High level GO category** | **Genes** |
| --- | --- | --- |
| 20 | Response to stress | CD44, MMP2, SERPINE1, SERPINE2, CAB39, CLU, CNN2, TIMP1, COL1A1, LOX, SPARC, FN1, POSTN, C1R, FSTL1, IGFBP7, HTRA1, COL1A2, CTSB, B2M |
| 15 | Anatomical structure morphogenesis | COL23A1, THBS2, SERPINE1, SPARC, FN1, CLU, MMP2, COL1A1, LOX, POSTN, SERPINE2, HTRA1, TGFBI, CD44, COL1A2 |
| 14 | Regulation of response to stimulus | CD44, SERPINE1, SERPINE2, COL1A1, FN1, B2M, TIMP1, LOX, CLU, POSTN, C1R, HTRA1, COL1A2, CTSB |
| 13 | Immune system process | CD44, CLU, B2M, CNN2, LOX, C1R, HTRA1, SERPINE1, COL1A1, FN1, CAB39, COL1A2, CTSB |
| 13 | Response to external stimulus | COL1A1, SERPINE1, SERPINE2, CNN2, POSTN, LOX, SPARC, LOXL1, FSTL1, HTRA1, B2M, CLU, C1R |
| 12 | Response to endogenous stimulus | TIMP1, CD44, COL1A2, MMP2, COL1A1, LOX, SPARC, POSTN, IGFBP7, HTRA1, CTSB, FSTL1 |
| 12 | Regulation of localization | CAB39, SERPINE1, COL1A1, SPARC, FN1, CNN2, NUCB1, POSTN, SERPINE2, CLU, B2M, TIMP1 |
| 12 | Multi-organism process | FN1, MMP2, SPARC, LOXL1, SERPINE2, IGFBP7, CTSB, HTRA1, B2M, CLU, SERPINE1, TIMP1 |
| 11 | Locomotion | CD44, SERPINE1, COL1A1, SPARC, FN1, CNN2, LOX, POSTN, TIMP1, SERPINE2, COL1A2 |
| 11 | Cell motility | CD44, SERPINE1, COL1A1, SPARC, FN1, CNN2, LOX, POSTN, TIMP1, SERPINE2, COL1A2 |
| 11 | Regulation of developmental process | THBS2, SERPINE1, COL1A1, SPARC, SERPINE2, LOX, FN1, POSTN, B2M, CD44, TIMP1 |
| 11 | Regulation of multicellular organismal process | THBS2, SERPINE1, SERPINE2, COL1A1, SPARC, FN1, CLU, B2M, LOX, POSTN, TIMP1 |
| 11 | Localization of cell | CD44, SERPINE1, COL1A1, SPARC, FN1, CNN2, LOX, POSTN, TIMP1, SERPINE2, COL1A2 |
| 11 | Regulation of biological quality | B2M, SERPINE1, SERPINE2, CLU, LOX, SPARC, FN1, POSTN, THBS2, COL1A2, COL1A1 |
| 11 | Regulation of molecular function | TIMP1, SERPINE1, SERPINE2, CAB39, CD44, B2M, PCOLCE, LOX, FN1, CTSB, CLU |
| 10 | Cell adhesion | CD44, TGFBI, POSTN, SERPINE1, FN1, IGFBP7, COL1A1, SERPINE2, SVEP1, THBS2 |
| 10 | Cell proliferation | TIMP1, SPARC, FN1, CLU, CNN2, MMP2, TGFBI, SERPINE2, HTRA1, IGFBP7 |
| 10 | Biological adhesion | CD44, TGFBI, POSTN, SERPINE1, FN1, IGFBP7, COL1A1, SERPINE2, SVEP1, THBS2 |
| 10 | Regulation of signalling | CD44, SERPINE1, COL1A1, FN1, TIMP1, LOX, POSTN, SERPINE2, HTRA1, CLU |
| 9 | Regulation of immune system process | B2M, LOX, CLU, C1R, HTRA1, SERPINE1, COL1A1, COL1A2, CTSB |
| 9 | Immune response | B2M, CLU, C1R, CD44, CNN2, COL1A1, CAB39, COL1A2, CTSB |
| 9 | Anatomical structure formation involved in morphogenesis | COL23A1, THBS2, SERPINE1, SPARC, FN1, MMP2, COL1A1, HTRA1, TGFBI |
| 8 | Immune effector process | B2M, CLU, C1R, HTRA1, CD44, CNN2, CAB39, CTSB |
| 8 | Response to abiotic stimulus | MMP2, COL1A1, CAB39, CNN2, SPARC, POSTN, SERPINE2, IGFBP7 |
| 8 | Regulation of locomotion | SERPINE1, COL1A1, SPARC, FN1, CNN2, POSTN, TIMP1, SERPINE2 |
| 7 | Catabolic process | MMP2, TIMP1, CLU, CTSB, CD44, SERPINE2, HEXA |
| 7 | Regulation of cell adhesion | SERPINE1, FN1, COL1A1, POSTN, SERPINE2, CD44, TGFBI |
| 7 | Cellular component biogenesis | CLU, COL1A1, LOX, FN1, CAB39, COL1A2, THBS2 |
| 6 | Reproduction | MMP2, SERPINE2, IGFBP7, CTSB, HTRA1, TIMP1 |
| 6 | Response to biotic stimulus | SPARC, LOXL1, HTRA1, B2M, CLU, SERPINE1 |
| 6 | Reproductive process | MMP2, SERPINE2, IGFBP7, CTSB, HTRA1, TIMP1 |
| 6 | Leukocyte activation | CD44, CLU, B2M, CNN2, CAB39, CTSB |
| 6 | Response to other organism | SPARC, LOXL1, HTRA1, B2M, CLU, SERPINE1 |
| 5 | System process | TGFBI, POSTN, SERPINE2, COL1A1, COL1A2 |
| 5 | Macromolecule localization | CLU, COL1A1, FN1, NUCB1, POSTN |
| 5 | Multi-organism reproductive process | MMP2, SERPINE2, IGFBP7, CTSB, TIMP1 |
| 5 | Multi-multicellular organism process | MMP2, SERPINE2, IGFBP7, CTSB, TIMP1 |
| 5 | Leukocyte migration | SERPINE1, CD44, COL1A1, FN1, COL1A2 |
| 4 | Cell growth | FN1, POSTN, SERPINE2, IGFBP7 |
| 4 | Growth | FN1, POSTN, SERPINE2, IGFBP7 |
| 3 | Activation of immune response | CLU, C1R, CTSB |
| 3 | Immune system development | CNN2, LOX, B2M |
| 3 | Developmental process involved in reproduction | SERPINE2, CTSB, HTRA1 |
| 3 | Regulation of growth | FN1, SERPINE2, IGFBP7 |
| 3 | Interspecies interaction between organisms | FN1, CTSB, B2M |
| 3 | Cellular localization ","CLU COL1A1 NUCB1" | CLU, COL1A1, NUCB1 |
| 2 | Protein folding | CLU, B2M |
| 2 | Multicellular organism reproduction | SERPINE2, CTSB |
| 2 | Taxis | LOX, SERPINE1 |
| 2 | Regulation of multi-organism process | HTRA1, TIMP1 |
| 2 | Regulation of cellular component biogenesis | CLU, THBS2 |
| 2 | Developmental growth | FN1, POSTN |
| 2 | Multicellular organismal reproductive process | SERPINE2, CTSB |
| 2 | Protein activation cascade | CLU, C1R |
| 2 | Regulation of homeostasis | SERPINE1, SERPINE2 |

Supplementary table 4 Protein findings from proteome analysis of cell lysates from breast cancer cells co-cultured with osteosarcoma cells (MG-63) compared to MCF-7 monoculture. Information about gene symbol/User ID, Ensemble Gene ID, p-value and mean values of co-culture and MCF-7 control is given. List was used to further examine Gene Ontology (GO) enrichment using Shiny GO v06.0.

| **i** | **User ID** | **Ensemble Gene ID** | **p-value** | **mean co-culture** | **mean MCF-7 control** |
| --- | --- | --- | --- | --- | --- |
| 1 | HIST2H3PS2 | ENSG00000203818 | <0,000000000000001 | 711875 | 1418125 |
| 2 | CFL1 | ENSG00000172757 | 3,995E-07 | 1362500 | 1800000 |
| 3 | SLC3A2 | ENSG00000168003 | 4,69E-13 | 1318625 | 694125 |
| 4 | KRT8 | ENSG00000170421 | <0,000000000000001 | 2528750 | 5233750 |
| 5 | HSP90B1 | ENSG00000166598 | <0,000000000000001 | 3183750 | 2406250 |
| 6 | KRT18 | ENSG00000111057 | <0,000000000000001 | 1963750 | 4050125 |
| 7 | ATP5F1B | ENSG00000110955 | 1,11105E-06 | 1610000 | 1189625 |
| 8 | PDIA3 | ENSG00000167004 | 1,48704E-06 | 1531250 | 1115875 |
| 9 | KRT19 | ENSG00000171345 | <0,000000000000001 | 1441250 | 2472250 |
| 10 | FASN | ENSG00000169710 | <0,000000000000001 | 3546250 | 2232500 |
| 11 | ATP5F1A | ENSG00000152234 | 5,68907E-05 | 1645000 | 1297625 |
| 12 | HSPD1 | ENSG00000144381 | 7,80485E-09 | 1988750 | 1490500 |
| 13 | PREX1 | ENSG00000124126 | 6,12649E-06 | 304250 | 694500 |
| 14 | HSPA9 | ENSG00000113013 | 2,96513E-07 | 1378750 | 936375 |
| 15 | HSP90AB1 | ENSG00000096384 | 2,19282E-08 | 1622500 | 1139500 |
| 16 | MDH2 | ENSG00000146701 | 7,73663E-08 | 2807500 | 2343750 |
| 17 | HSPB1 | ENSG00000106211 | <0,000000000000001 | 4387500 | 6976250 |

Supplementary table 5 GO enrichment analysis of findings from proteome analysis. Protein discoveries from co-cultured breast cancer cell lysates listed in supplementary table 4 were examined for GO enrichment using Shiny GO v06.0. Information is given about the enrichment FDR, how many genes with the discovery list are enriched within specific functional category, total number of genes within specific functional category, functional category and genes listed from the discoveries which are enriched in specific category.

| **Enrichment FDR** | **Genes in list** | **Total genes** | **Functional Category** | **Genes** |
| --- | --- | --- | --- | --- |
| 7,55E-06 | 6 | 245 | Protein folding | HSPA9, HSPD1, PDIA3, HSP90AB1, HSP90B1, HSPB1 |
| 1,34E-05 | 11 | 2257 | Programmed cell death | HSPD1, KRT18, HSP90AB1, HSPB1, PDIA3, KRT8, HSP90B1, HSPA9, PREX1, KRT19, CFL1 |
| 1,34E-05 | 9 | 1278 | Cellular response to cytokine stimulus | HSP90AB1, ATP5F1B, KRT18, PDIA3, FASN, KRT8, HSPA9, HSP90B1, CFL1 |
| 1,46E-05 | 11 | 2415 | Cell death | HSPD1, KRT18, HSP90AB1, HSPB1, PDIA3, KRT8, HSP90B1, HSPA9, PREX1, KRT19, CFL1 |
| 1,48E-05 | 9 | 1372 | Response to cytokine | HSP90AB1, ATP5F1B, KRT18, PDIA3, FASN, KRT8, HSPA9, HSP90B1, CFL1 |
| 1,82E-05 | 5 | 193 | Response to unfolded protein | HSPA9, HSPD1, HSP90AB1, HSPB1, HSP90B1 |
| 2,97E-05 | 10 | 2106 | Apoptotic process | HSPD1, KRT18, HSP90AB1, HSPB1, PDIA3, KRT8, HSP90B1, HSPA9, PREX1, CFL1 |
| 2,97E-05 | 12 | 3547 | Response to organic substance | HSPA9, HSP90AB1, HSPD1, HSP90B1, ATP5F1B, KRT18, PDIA3, FASN, KRT8, CFL1, HSPB1, SLC3A2 |
| 2,97E-05 | 5 | 227 | Response to topologically incorrect protein | HSPA9, HSPD1, HSP90AB1, HSPB1, HSP90B1 |
| 3,69E-05 | 9 | 1657 | Regulation of apoptotic process | KRT18, HSPD1, HSP90AB1, HSPB1, PDIA3, HSP90B1, HSPA9, PREX1, CFL1 |
| 3,79E-05 | 9 | 1681 | Regulation of programmed cell death | KRT18, HSPD1, HSP90AB1, HSPB1, PDIA3, HSP90B1, HSPA9, PREX1, CFL1 |
| 7,27E-05 | 9 | 1835 | Regulation of cell death | KRT18, HSPD1, HSP90AB1, HSPB1, PDIA3, HSP90B1, HSPA9, PREX1, CFL1 |
| 0,000115066 | 7 | 966 | Negative regulation of apoptotic process | KRT18, HSP90AB1, HSPB1, HSPD1, HSP90B1, HSPA9, CFL1 |
| 0,00012323 | 7 | 987 | Negative regulation of programmed cell death | KRT18, HSP90AB1, HSPB1, HSPD1, HSP90B1, HSPA9, CFL1 |
| 0,000191624 | 11 | 3536 | Cellular response to chemical stimulus | HSPA9, HSP90AB1, HSP90B1, HSPB1, ATP5F1B, KRT18, PREX1, PDIA3, FASN, KRT8, CFL1 |
| 0,000219305 | 7 | 1099 | Negative regulation of cell death | KRT18, HSP90AB1, HSPB1, HSPD1, HSP90B1, HSPA9, CFL1 |
| 0,000225829 | 6 | 712 | Supramolecular fiber organization | HSP90B1, KRT19, CFL1, PREX1, KRT8, HSP90AB1 |
| 0,000225829 | 3 | 55 | Positive regulation of blood vessel endothelial cell migration | ATP5F1B, ATP5F1A, HSPB1 |
| 0,000251526 | 10 | 2938 | Cellular response to organic substance | HSPA9, HSP90AB1, HSP90B1, ATP5F1B, KRT18, PDIA3, FASN, KRT8, HSPB1, CFL1 |
| 0,000790234 | 2 | 12 | Hepatocyte apoptotic process | KRT18, KRT8 |
| 0,000911637 | 6 | 951 | Viral process | KRT18, HSPD1, KRT8, KRT19, HSP90AB1, CFL1 |
| 0,000911637 | 6 | 950 | Cytokine-mediated signalling pathway | KRT18, KRT8, HSP90AB1, HSPA9, HSP90B1, CFL1 |
| 0,000915372 | 3 | 95 | Regulation of blood vessel endothelial cell migration | ATP5F1B, ATP5F1A, HSPB1 |
| 0,000962601 | 3 | 98 | Positive regulation of endothelial cell migration | ATP5F1B, ATP5F1A, HSPB1 |
| 0,001203543 | 3 | 109 | Mitochondrial transmembrane transport | HSPD1, ATP5F1B, ATP5F1A |
| 0,001203543 | 2 | 17 | Protein folding in endoplasmic reticulum | HSP90B1, PDIA3 |
| 0,001203543 | 6 | 1024 | Symbiont process | KRT18, HSPD1, KRT8, KRT19, HSP90AB1, CFL1 |
| 0,001435777 | 3 | 119 | Blood vessel endothelial cell migration | ATP5F1B, ATP5F1A, HSPB1 |
| 0,001435777 | 6 | 1084 | Interspecies interaction between organisms | KRT18, HSPD1, KRT8, KRT19, HSP90AB1, CFL1 |
| 0,001588854 | 3 | 125 | Cornification | KRT18, KRT8, KRT19 |

Supplementary table 6: GO group enrichment analysis of findings from proteome analysis. Protein discoveries listed in Supplementary table 4 were examined for GO group enrichment using Shiny GO v06.0.

| **N** | **High level GO category** | **Genes** |
| --- | --- | --- |
| 9 | Regulation of biological quality | HSPB1, HSP90AB1, PREX1, PDIA3, CFL1, ATP5F1B, HSPA9, HSPD1, HSP90B1 |
| 8 | Immune system process | HSPD1, HSPA9, PREX1, FASN, HSP90AB1, HSP90B1, PDIA3, SLC3A2 |
| 8 | Response to stress | HSPB1, HSPA9, ATP5F1A, HSP90B1, PDIA3, HSPD1, HSP90AB1, KRT8 |
| 8 | Anatomical structure morphogenesis | KRT19, HSP90AB1, PREX1, KRT8, CFL1, HSPB1, ATP5F1B, KRT18 |
| 8 | Regulation of multicellular organismal process | HSP90AB1, HSPD1, HSPB1, HSPA9, PREX1, ATP5F1B, ATP5F1A, CFL1 |
| 8 | Cellular localization | HSP90AB1, HSPD1, ATP5F1B, ATP5F1A, KRT18, HSPA9, HSPB1, HSP90B1 |
| 7 | Cellular component biogenesis | HSP90AB1, HSP90B1, KRT19, HSPA9, PREX1, KRT8, HSPD1 |
| 7 | Multi-organism process | KRT18, HSPD1, KRT8, KRT19, HSPB1, CFL1, HSP90AB1 |
| 6 | Protein folding | HSPA9, HSPD1, PDIA3, HSP90AB1, HSP90B1, HSPB1 |
| 6 | Biological adhesion | HSPB1, HSPD1, ATP5F1B, KRT18, PREX1, HSP90AB1 |
| 6 | Macromolecule localization | HSP90AB1, HSPD1, KRT18, HSPA9, HSPB1, HSP90B1 |
| 6 | Locomotion | ATP5F1B, PREX1, CFL1, ATP5F1A, HSPB1, SLC3A2 |
| 6 | Interspecies interaction between organisms | KRT18, HSPD1, KRT8, KRT19, HSP90AB1, CFL1 |
| 6 | Regulation of response to stimulus | HSP90AB1, HSPD1, HSPB1, PREX1, PDIA3, HSP90B1 |
| 6 | Cell motility | ATP5F1B, PREX1, CFL1, ATP5F1A, HSPB1, SLC3A2 |
| 6 | Localization of cell | ATP5F1B, PREX1, CFL1, ATP5F1A, HSPB1, SLC3A2 |
| 5 | Cell adhesion | HSPB1, HSPD1, ATP5F1B, KRT18, PREX1 |
| 5 | Response to external stimulus | HSP90B1, PREX1, KRT8, HSPB1, CFL1 |
| 5 | Response to abiotic stimulus | HSPA9, HSP90B1, HSP90AB1, KRT8, HSPD1 |
| 5 | Regulation of localization | HSP90AB1, PREX1, ATP5F1B, ATP5F1A, HSPB1 |
| 5 | Anatomical structure formation involved in morphogenesis | KRT19, KRT8, CFL1, HSPB1, ATP5F1B |
| 5 | Regulation of developmental process | HSPA9, PREX1, HSP90AB1, HSPB1, CFL1 |
| 5 | Regulation of molecular function | HSP90AB1, HSPD1, HSP90B1, HSPB1, PREX1 |
| 4 | Immune system development | HSPD1, HSPA9, PREX1, FASN |
| 4 | Regulation of immune system process | HSPD1, HSPA9, HSP90AB1, HSP90B1 |
| 4 | Response to biotic stimulus | HSPD1, KRT8, HSPB1, CFL1 |
| 4 | Regulation of signalling | HSP90AB1, HSPB1, PREX1, PDIA3 |
| 4 | Regulation of locomotion | PREX1, ATP5F1B, ATP5F1A, HSPB1 |
| 3 | Reproduction | HSP90AB1, KRT8, KRT19 |
| 3 | Activation of immune response | HSPD1, HSP90AB1, HSP90B1 |
| 3 | Developmental process involved in reproduction | HSP90AB1, KRT8, KRT19 |
| 3 | Immune response | HSPD1, HSP90AB1, HSP90B1 |
| 3 | Catabolic process | HSP90AB1, HSP90B1, HSPB1 |
| 3 | Reproductive process | HSP90AB1, KRT8, KRT19 |
| 3 | Regulation of cell adhesion | HSPD1, ATP5F1B, PREX1 |
| 3 | Leukocyte activation | HSPD1, PREX1, HSP90AB1 |
| 3 | Response to other organism | KRT8, HSPB1, CFL1 |
| 2 | Immune effector process | HSPD1, HSP90AB1 |
| 2 | Cell proliferation | HSPD1, ATP5F1A |
| 2 | Response to endogenous stimulus | HSP90AB1, HSP90B1 |
| 2 | Cell cycle process | CFL1, HSP90AB1 |
| 2 | Taxis | PREX1, HSPB1 |
| 2 | Leukocyte migration | PREX1, SLC3A2 |

Supplementary table 7a: CTGF expression in invasive ductal carcinoma and normal breast tissue. CTGF expression was assessed in 24 patient samples (female patients aged 19 to 79 years) from biomax tissue array (BR248a). Following information are given: Sex/Age, pathology diagnosis, TNM (Tumor, Node, and Metastasis), Tumor Grading, Stage, Type, Tissue-ID and corresponding detected CTGF expression.

| **Pathology diagnosis** | **TNM** | **Grade** | **Stage** | **Type** | **Tissue ID.** | **CTGF expression** |
| --- | --- | --- | --- | --- | --- | --- |
| Invasive ductal carcinoma | T3N0M0 | 1--2 | IIB | malignant | Fmg040048 | + |
| Invasive ductal carcinoma | T2N0M0 | 1--2 | IIA | malignant | Fmg020357 | + |
| Invasive ductal carcinoma | T2N0M0 | 2 | IIA | malignant | Fmg040031 | + |
| Invasive ductal carcinoma | T2N0M0 | 2 | IIA | malignant | Fmg040001 | + |
| Invasive ductal carcinoma | T1N0M0 | 2 | I | malignant | Fmg040052 | ++ |
| Invasive ductal carcinoma | T2N0M0 | 2 | IIA | malignant | Fmg040104 | - |
| Invasive ductal carcinoma | T2N0M0 | 2 | IIA | malignant | Fmg040113 | ++ |
| Invasive ductal carcinoma | T2N0M0 | 2 | IIA | malignant | Fmg040118 | +/++ |
| Invasive ductal carcinoma | T2N0M0 | 2 | IIA | malignant | Fmg040120 | ++ |
| Invasive ductal carcinoma | T2N0M0 | 2 | IIA | malignant | Fmg040123 | ++ |
| Invasive ductal carcinoma | T2N0M0 | 2 | IIA | malignant | Fmg040125 | + |
| Invasive ductal carcinoma | T2N0M0 | 2 | IIA | malignant | Fmg040130 | ++ |
| Invasive ductal carcinoma | T2N1M0 | 2 | IIB | malignant | Fmg040131 | + |
| Invasive ductal carcinoma | T2N1M0 | 3 | IIB | malignant | Fmg010491 | + |
| Invasive ductal carcinoma | T2N0M0 | 3 | IIA | malignant | Fmg040004 | - |
| Invasive ductal carcinoma | T3N0M0 | 3 | IIB | malignant | Fmg040074 | + |
| Invasive ductal carcinoma | T4N0M0 | 3 | IIIB | malignant | Fmg010789 | + |
| Medullary carcinoma | T2N0M0 | - | IIA | malignant | Fmg040016 | + |
| Adenosis | - | - | - | normal | Fmg06N024 | - |
| Normal breast tissue (fibro fatty tissue and blood vessel) | - | - | - | normal | Fmg11N017 | - |
| Normal breast tissue | - | - | - | normal | Fmg12N001 | + |
| Adenosis | - | - | - | normal | Fmg08N034 | - |
| Normal breast tissue | - | - | - | normal | Fmg12N002 | - |
| Normal breast tissue | - | - | - | normal | Fmg07N013 | - |

Supplementary table 7b: CTGF expression in breast carcinoma, normal and other non-malignant breast tissues. CTGF expression was assessed in 47 patient samples (female patients aged 16 to 75 years) from biomax tissue array (BRC961). Following information are given: Age, pathology diagnosis, TNM (Tumor, Node, and Metastasis), Tumor Grading, AR/ER/PR/Her-2 IHC results and corresponding detected CTGF expression.

| **Pathology diagnosis** | **TNM** | **Grade** | **AR** | **ER** | **PR** | **HER2** | **CTGF** |
| --- | --- | --- | --- | --- | --- | --- | --- |
| Normal |  |  | +~++, 30% | +~++, 15% | - | - | - |
| Normal |  |  | +~++, 20% | +, 5% | ++, 15% | ++ | + |
| Normal |  |  | +~++, 20% | +~++, 15% | +, 5% | - | - |
| Periductual mastitis |  |  | +~++, 20% | +~++, 5% | ++, 5% | - | + |
| Hyperplasia |  |  | - | +~++, 10% | ++, 10% | - | ++ |
| Hyperplasia |  |  | - | +~++, 5% | ++, 5% | - | ++ |
| Hyperplasia |  |  | +, 20% | +, 15% | ++, 20% | - | +~++ |
| Fibrocystic changes |  |  | +~++, 20% | +~++, 15% | +~++, 20% | + | + |
| Fibrocystic changes |  |  | +, 20% | +~++, 15% | ++~+++, 30% | + | + |
| Fibroadenoma |  |  | +, 5% | +~++, 5% | +~++, 10% | + | + |
| Fibroadenoma |  |  | +, 50% | ++~+++, 80% | ++~+++, 80% | +- | +++ |
| Fibroadenoma |  |  | +~++, 30% | ++~+++, 20% | ++~+++, 20% | + | ++ |
| Normal |  |  | +~++, 30% | +~++, 15% | - | - | - |
| Normal |  |  | +~++, 20% | +, 5% | ++, 15% | ++ | + |
| Normal |  |  | +~++, 20% | +~++, 15% | +, 5% | - | - |
| Periductual mastitis |  |  | +~++, 20% | +~++, 5% | ++, 5% | - | + |
| Hyperplasia |  |  | - | +~++, 10% | ++, 10% | - | ++ |
| Hyperplasia |  |  | - | +~++, 5% | ++, 5% | - | ++ |
| Hyperplasia |  |  | +, 20% | +, 15% | ++, 20% | - | + |
| Fibrocystic changes |  |  | +~++, 20% | +~++, 15% | +~++, 20% | + | ++ |
| Fibrocystic changes |  |  | +, 20% | +~++, 15% | ++~+++, 30% | + | ++ |
| Fibroadenoma |  |  | +, 5% | +~++, 5% | +~++, 10% | + | ++ |
| Fibroadenoma |  |  | +, 50% | ++~+++, 80% | ++~+++, 80% | +- | +++ |
| Fibroadenoma |  |  | +~++, 30% | ++~+++, 20% | ++~+++, 20% | + | ++ |
| Phyllodes sarcoma | TisN0M0 |  | +~++, 10% | +~++, 10% | ++~+++, 50% | +~++ | ++ |
| Intraductal carcinoma | TisN0M0 | I | +- | +++, 80% | - | ++~+++ | ++ |
| Intraductal carcinoma | TisN0M0 | I | +- | ++, 20% | ++~+++, 50% | ++~+++ | ++ |
| Invasive ductal carcinoma (partially intraductal carcinoma) | T3N1M1 | I~II | +~++, 20% | ++~+++, 60% | ++~+++, 50% | + | ++~+++ |
| Ductal carcinoma in situ | TisN0M0 | I | +, 15% | - | - | +++ | +++ |
| Invasive ductal carcinoma | T2N0M0 | II | ++, 5% | ++, 5% | ++~+++, 10% | + | +++(+) |
| Invasive ductal carcinoma | T3N2M0 | III | +~++, 5% | +, 50% | +~++, 5% | + | +++ |
| Invasive ductal carcinoma | T3N0M0 | II~III | - | - | - | - | ++ |
| Invasive ductal carcinoma | T2N0M0 | II | ++, 20% | - | - | ++~+++ | ++ |
| Invasive ductal carcinoma | T3N2M0 | II | - | +, 5% | - | +++ | +++ |
| Invasive ductal carcinoma | T2N0M0 | II~III | +- | +~++, 5% | +~++, 5% | + | ++ |
| Phyllodes sarcoma | TisN0M0 |  | +~++, 10% | +~++, 10% | ++~+++, 50% | +~++ | ++ |
| Intraductal carcinoma | TisN0M0 | I | +- | +++, 80% | - | ++~+++ | ++ |
| Intraductal carcinoma | TisN0M0 | I | +- | ++, 20% | ++~+++, 50% | ++~+++ | ++ |
| Invasive ductal carcinoma (partially intraductal carcinoma) | T3N1M1 | I~II | +~++, 20% | ++~+++, 60% | ++~+++, 50% | + | ++ |
| Ductal carcinoma in situ | TisN0M0 | I | +, 15% | - | - | +++ | +++ |
| Invasive ductal carcinoma | T2N0M0 | II | ++, 5% | ++, 5% | ++~+++, 10% | + | +++ |
| Invasive ductal carcinoma | T3N2M0 | III | +~++, 5% | +, 50% | +~++, 5% | + | +++ |
| Invasive ductal carcinoma | T3N0M0 | II~III | - | - | - | - | ++ |
| Invasive ductal carcinoma | T2N0M0 | II | ++, 20% | - | - | ++~+++ | +~++ |
| Invasive ductal carcinoma | T3N2M0 | II | - | +, 5% | - | +++ | ++ |
| Invasive ductal carcinoma | T2N0M0 | II~III | +- | +~++, 5% | +~++, 5% | + | ++ |
| Invasive ductal carcinoma | T2N0M0 | II~III | +- | - | - | +++ | ++ |
| Invasive ductal carcinoma | T2N0M0 | II | - | - | - | +++ | +++ |
| Invasive ductal carcinoma | T3N0M0 | III | ++, 30% | +, 10% | +~++, 10% | - | +++ |
| Invasive ductal carcinoma | T3N0M0 | I~II | - | - | +~++, 5% | - | ++ |
| Invasive ductal carcinoma | T3N0M0 | III | - | - | - | - | +++ |
| Invasive ductal carcinoma | T2N0M0 | III | - | - | +~++, 5% | +++ | +++(+) |
| Invasive ductal carcinoma | T4N2MX | II~III | - | +, 50% | ++~+++, 60% | + | +++(+) |
| Invasive ductal carcinoma | T2N0M0 | II | - | - | - | - | +++ |
| Invasive ductal carcinoma | T3N0M0 | I~II | - | ++~+++, 50% | +~++, 50% | +- | +++ |
| Invasive ductal carcinoma | T2N0M0 | III | +- | +, 5% | - | + | +++ |
| Invasive ductal carcinoma | T3N1M0 | II | - | +, 10% | +~++, 10% | - | +++ |
| Invasive ductal carcinoma | T3N0M0 | II | - | - | - | ++~+++ | +++ |
| Invasive ductal carcinoma | T2N0M0 | II~III | +- | - | - | +++ | ++ |
| Invasive ductal carcinoma | T2N0M0 | II | - | - | - | +++ | +++ |
| Invasive ductal carcinoma | T3N0M0 | III | ++, 30% | +, 10% | +~++, 10% | - | ++ |
| Invasive ductal carcinoma | T3N0M0 | I~II | - | - | +~++, 5% | - | ++ |
| Invasive ductal carcinoma | T3N0M0 | III | - | - | - | - | +++ |
| Invasive ductal carcinoma | T2N0M0 | III | - | - | +~++, 5% | +++ | ++~+++ |
| Invasive ductal carcinoma | T4N2MX | II~III | - | +, 50% | ++~+++, 60% | + | ++~+++ |
| Invasive ductal carcinoma | T2N0M0 | II | - | - | - | - | +++ |
| Invasive ductal carcinoma | T3N0M0 | I~II | - | ++~+++, 50% | +~++, 50% | +- | ++ |
| Invasive ductal carcinoma | T2N0M0 | III | +- | +, 5% | - | + | +++ |
| Invasive ductal carcinoma | T3N1M0 | II | - | +, 10% | +~++, 10% | - | ++ |
| Invasive ductal carcinoma | T3N0M0 | II | - | - | - | ++~+++ | +++ |
| Invasive ductal carcinoma | T2N0M0 | III | ++~+++, 60% | ++~+++, 30% | +~++, 10% | +++ | ++ |
| Invasive ductal carcinoma | T2N0M0 | II~III | ++~+++, 10% | +~++, 10% | - | +++ | + |
| Invasive ductal carcinoma | T4N3M1 | II~III | - | +, 5% | +, 5% | + | ++ |
| Invasive ductal carcinoma | T2N0M0 | II~III | +, 5% | ++~+++, 30% | ++~+++, 50% | +- | - |
| Invasive ductal carcinoma | T3N1M0 | II | +- | ++, 50% | +~++, 15% | - | ++ |
| Invasive ductal carcinoma | T4N2MX | II~III | - | - | - | +++ | ++ |
| Invasive ductal carcinoma | T3N1M0 | II~III | +- | - | - | +~++ | ++ |
| Invasive ductal carcinoma | T3N0M0 | III | - | - | - | +++ | +++ |
| Invasive ductal carcinoma | T3N1M0 | III | - | - | - | ++~+++ | +++(+) |
| Invasive ductal carcinoma | T3N1M0 | II~III | +~++, 5% | ++~+++, 20% | ++~+++, 60% | + | +++ |
| Invasive mucinous adenocarcinoma | T3N2M0 |  | - | - | - | +~++ | + |
| Invasive lobular carcinoma | T2N0M0 | III | +- | ++~+++, 50% | +~++, 15% | - | ++ |
| Invasive ductal carcinoma | T2N0M0 | III | ++~+++, 60% | ++~+++, 30% | +~++, 10% | +++ | + |
| Invasive ductal carcinoma | T2N0M0 | II~III | ++~+++, 10% | +~++, 10% | - | +++ | + |
| Invasive ductal carcinoma | T4N3M1 | II~III | - | +, 5% | +, 5% | + | ++~+++ |
| Invasive ductal carcinoma | T2N0M0 | II~III | +, 5% | ++~+++, 30% | ++~+++, 50% | +- | - |
| Invasive ductal carcinoma | T3N1M0 | II | +- | ++, 50% | +~++, 15% | - | +++(+) |
| Invasive ductal carcinoma | T4N2MX | II~III | - | - | - | +++ | +++ |
| Invasive ductal carcinoma | T3N1M0 | II~III | +- | - | - | +~++ | +++ |
| Invasive ductal carcinoma | T3N0M0 | III | - | - | - | +++ | +++ |
| Invasive ductal carcinoma | T3N1M0 | III | - | - | - | ++~+++ | +++(+) |
| Invasive ductal carcinoma | T3N1M0 | II~III | +~++, 5% | ++~+++, 20% | ++~+++, 60% | + | +++ |
| Invasive mucinous adenocarcinoma | T3N2M0 |  | - | - | - | +~++ | + |
| Invasive lobular carcinoma | T2N0M0 | III | +- | ++~+++, 50% | +~++, 15% | - | ++ |
| Unknown tissue marker | |  |  |  |  |  | - |
| Unknown tissue marker | |  |  |  |  |  | - |

Supplementary table 7c: CTGF expression in breast carcinoma and matched lymph node metastases. CTGF expression was assessed in 104 patient samples (female patients aged 19 to 87 years) from biomax tissue array (BR20837). Following information are given: Age, organ/anatomic site, pathology diagnosis, TNM (Tumor, Node, and Metastasis), Tumor Grading, Stage, AR/ER/PR/Her-2 IHC results and corresponding detected CTGF expression.

| **Organ/Anatomic Site** | **Pathology diagnosis** | **TNM** | **Grade** | **Stage** | **ER** | **PR** | **HER2** | **CTGF** |
| --- | --- | --- | --- | --- | --- | --- | --- | --- |
| Breast | Invasive ductal carcinoma | T1N1M0 | 1 | IIA | - | - | 0 | + |
| Lymph node | Metastatic carcinoma | - | - | - | - | - | 0 | + |
| Breast | Invasive ductal carcinoma | T2N2M0 | 1 | IIIA | + | - | 0 | ++ |
| Lymph node | Metastatic carcinoma | - | - | - | - | + | 0 | +++ |
| Breast | Invasive ductal carcinoma | T2N1M0 | 1 | IIB | - | - | 0 | ++ |
| Lymph node | Metastatic carcinoma (lymph node tissue) | - | - | - | - | - | 0 | +++ |
| Breast | Invasive ductal carcinoma | T2N1M0 | 1 | IIB | - | - | 1+ | - |
| Lymph node | Metastatic carcinoma | - | - | - | - | - | 0 | - |
| Breast | Invasive ductal carcinoma | T3N1M0 | 1 | IIIA | +++ | - | 0 | ++ |
| Lymph node | Metastatic carcinoma | - | - | - | +++ | - | 0 | +++ |
| Breast | Invasive ductal carcinoma | T4N1M0 | 1 | IIIB | + | - | 0 | +++ |
| Lymph node | Metastatic carcinoma | - | - | - | +++ | + | 0 | +++ |
| Breast | Invasive ductal carcinoma | T2N1M0 | 1 | IIB | + | ++ | 3+ | ++ |
| Lymph node | Metastatic carcinoma | - | - | - | - | - | 3+ | ++ |
| Breast | Invasive ductal carcinoma | T2N1M0 | 1 | IIB | - | - | 3+ | + |
| Lymph node | Metastatic carcinoma from No.15 | - | - | - | - | - | 3+ | ++ |
| Breast | Invasive ductal carcinoma | T2N1M0 | 2 | IIB | - | - | 0 | ++ |
| Lymph node | Metastatic carcinoma | - | - | - | - | - | 2+ | +++ |
| Breast | Invasive ductal carcinoma | T2N1M0 | 2 | IIB | - | - | 3+ | ++ |
| Lymph node | Metastatic carcinoma | - | - | - | - | - | 3+ | +++ |
| Breast | Invasive ductal carcinoma | T2N2M0 | 2 | IIIA | - | - | 3+ | +++ |
| Lymph node | Metastatic carcinoma | - | - | - | - | - | 3+ | ++ |
| Breast | Invasive ductal carcinoma (fibrofatty tissue and blood vessel) | T2N1M0 | - | IIB | - | - | * | ++ |
| Lymph node | Metastatic carcinoma | - | - | - | - | - | 3+ | +++ |
| Breast | Invasive ductal carcinoma | T2N2M0 | 2 | IIIA | ++ | - | 1+ | +++(+) |
| Lymph node | Metastatic carcinoma | - | - | - | ++ | - | 1+ | +++(+) |
| Breast | Invasive ductal carcinoma | T1N1M0 | 2 | IIA | ++ | - | 0 | + |
| Lymph node | Metastatic carcinoma | - | - | - | - | - | 0 | ++ |
| Breast | Invasive ductal carcinoma | T2N1M0 | 2 | IIB | - | - | 3+ | ++ |
| Lymph node | Metastatic carcinoma | - | - | - | - | - | 3+ | + |
| Breast | Invasive ductal carcinoma | T2N2M0 | 2 | IIIA | - | - | 3+ | +++ |
| Lymph node | Metastatic carcinoma | - | - | - | - | ++ | 0 | ++ |
| Breast | Invasive ductal carcinoma | T3N2M0 | 2 | IIIA | - | + | 0 | ++ |
| Lymph node | Metastatic carcinoma | - | - | - | + | ++ | 0 | +++ |
| Breast | Invasive ductal carcinoma | T2N2M0 | 2 | IIIA | + | - | 0 | ++ |
| Lymph node | Metastatic carcinoma | - | - | - | - | - | 0 | ++~+++ |
| Breast | Invasive ductal carcinoma (tumor necrosis) | T3N1M0 | - | IIB | - | - | * | +++ |
| Lymph node | Metastatic carcinoma | - | - | - | - | - | 0 | +++ |
| Breast | Invasive ductal carcinoma | T2N1M0 | 2 | IIB | + | - | 3+ | +++ |
| Lymph node | Metastatic carcinoma | - | - | - | - | - | 3+ | +++ |
| Breast | Invasive ductal carcinoma | T2N1M0 | 2 | IIB | - | - | 1+ | +++ |
| Lymph node | Metastatic carcinoma | - | - | - | - | - | 0 | ++ |
| Breast | Invasive ductal carcinoma | T4N2M0 | 2 | IIIB | - | - | 0 | ++ |
| Lymph node | Metastatic carcinoma | - | - | - | - | - | 0 | ++ |
| Breast | Invasive ductal carcinoma | T2N1M0 | 2 | IIB | - | - | 0 | +~++ |
| Lymph node | Metastatic carcinoma | - | - | - | - | - | 0 | ++~+++ |
| Breast | Invasive ductal carcinoma | T2N2M0 | 2 | IIIA | + | ++ | 0 | + |
| Lymph node | Metastatic carcinoma | - | - | - | ++ | ++ | 0 | +++ |
| Breast | Invasive ductal carcinoma | T4N1M0 | 2 | IIIB | +++ | - | 2+ | ++ |
| Lymph node | Metastatic carcinoma | - | - | - | +++ | - | 2+ | ++ |
| Breast | Invasive ductal carcinoma | T2N2M0 | 2 | IIIA | - | - | 3+ | ++ |
| Lymph node | Metastatic carcinoma | - | - | - | - | - | 3+ | +~++ |
| Breast | Invasive ductal carcinoma | T2N2M0 | 2 | IIIA | - | - | 0 | +~++ |
| Lymph node | Metastatic carcinoma (sparse carcinoma tissue) | - | - | - | - | - | 0 | +~++ |
| Breast | Invasive ductal carcinoma | T2N2M0 | 2 | IIIA | + | + | 2+ | ++ |
| Lymph node | Metastatic carcinoma | - | - | - | - | - | 0 | ++ |
| Breast | Invasive ductal carcinoma | T1N1M0 | 2 | IIA | ++ | + | 1+ | ++ |
| Lymph node | Metastatic carcinoma | - | - | - | ++ | ++ | 1+ | +++ |
| Breast | Invasive ductal carcinoma | T2N1M0 | 2 | IIB | - | - | 0 | + |
| Lymph node | Metastatic carcinoma | - | - | - | - | - | 0 | +++ |
| Breast | Invasive ductal carcinoma | T2N2M0 | 2 | IIIA | - | - | 3+ | ++~+++ |
| Lymph node | Metastatic carcinoma | - | - | - | - | - | 0 | +++ |
| Breast | Invasive ductal carcinoma | T2N1M0 | 2 | IIB | * | - | 3+ | ++~+++ |
| Lymph node | Metastatic carcinoma | - | - | - | * | - | 3+ | +++ |
| Breast | Invasive ductal carcinoma | T2N1M0 | 3 | IIB | +++ | + | 1+ | ++ |
| Lymph node | Metastatic carcinoma | - | - | - | +++ | + | 1+ | +++ |
| Breast | Invasive ductal carcinoma | T2N1M0 | 3 | IIB | - | - | 3+ | + |
| Lymph node | Metastatic carcinoma | - | - | - | - | - | 3+ | ++ |
| Breast | Invasive ductal carcinoma | T1N1M0 | 3 | IIA | + | - | 3+ | ++ |
| Lymph node | Metastatic carcinoma | - | - | - | ++ | - | 3+ | +++ |
| Breast | Invasive ductal carcinoma | T2N0M0 | 3 | IIA | + | + | 1+ | ++~+++ |
| Lymph node | Metastatic carcinoma | - | - | - | + | + | 1+ | +++ |
| Breast | Invasive ductal carcinoma | T4N1M0 | 3 | IIIB | - | * | * | +~++ |
| Lymph node | Metastatic carcinoma | - | - | - | - | - | 1+ | ++~+++ |
| Breast | Invasive ductal carcinoma | T2N1M0 | 3 | IIB | + | +++ | 2+ | ++ |
| Lymph node | Metastatic carcinoma | - | - | - | + | +++ | 2+ | +++(+) |
| Breast | Invasive ductal carcinoma | T2N1M0 | 3 | IIB | + | + | 0 | +++ |
| Lymph node | Metastatic carcinoma | - | - | - | + | + | 0 | +++ |
| Breast | Invasive ductal carcinoma | T2N2M0 | 3 | IIIA | + | ++ | 0 | - |
| Lymph node | Metastatic carcinoma | - | - | - | + | + | 0 | - |
| Breast | Invasive ductal carcinoma | T2N1M0 | 3 | IIB | +++ | - | 1+ | ++ |
| Lymph node | Metastatic carcinoma | - | - | - | +++ | - | 1+ | ++ |
| Breast | Invasive ductal carcinoma | T2N1M0 | 3 | IIB | +++ | - | 0 | + |
| Lymph node | Metastatic carcinoma | - | - | - | +++ | - | 0 | ++ |
| Breast | Invasive ductal carcinoma | T2N1M0 | 3 | IIB | - | - | 0 | ++ |
| Lymph node | Metastatic carcinoma | - | - | - | - | - | 0 | +++ |
| Breast | Invasive ductal carcinoma | T2N1M0 | 3 | IIB | - | - | 0 | ++~+++ |
| Lymph node | Metastatic carcinoma | - | - | - | - | - | 1+ | +++ |
| Breast | Invasive ductal carcinoma (degeneration tissue) | T2N1M0 | - | IIB | - | - | 0 | + |
| Lymph node | Metastatic carcinoma (degeneration tissue) | - | - | - | - | - | 0 | +~++ |
| Breast | Invasive ductal carcinoma | T2N1M0 | 3 | IIB | - | - | 3+ | ++ |
| Lymph node | Metastatic carcinoma | - | - | - | - | - | 3+ | +++ |
| Breast | Invasive ductal carcinoma | T2N1M0 | 3 | IIB | - | - | 2+ | ++ |
| Lymph node | Metastatic carcinoma | - | - | - | - | - | 2+ | ++ |
| Breast | Invasive ductal carcinoma | T2N1M0 | 3 | IIB | - | - | 0 | - |
| Lymph node | Metastatic carcinoma | - | - | - | - | - | 0 | - |
| Breast | Invasive ductal carcinoma | T3N1M0 | 3 | IIIA | - | - | 3+ | ++ |
| Lymph node | Metastatic carcinoma | - | - | - | - | - | 3+ | ++ |
| Breast | Invasive ductal carcinoma | T2N1M0 | 3 | IIB | - | - | 3+ | ++ |
| Lymph node | Metastatic carcinoma | - | - | - | - | - | 3+ | + |
| Breast | Invasive ductal carcinoma | T3N3M0 | 3 | IIIC | - | - | 1+ | ++ |
| Lymph node | Metastatic carcinoma | - | - | - | + | + | 1+ | + |
| Breast | Invasive ductal carcinoma | T2N1M0 | 3 | IIB | - | + | 2+ | ++ |
| Lymph node | Metastatic carcinoma | - | - | - | ++ | +++ | 2+ | +++ |
| Breast | Invasive ductal carcinoma | T2N1M0 | 3 | IIB | - | - | 0 | +~++ |
| Lymph node | Metastatic carcinoma | - | - | - | - | - | 0 | ++ |
| Breast | Invasive ductal carcinoma | T2N1M0 | 3 | IIB | - | - | 0 | ++ |
| Lymph node | Metastatic carcinoma | - | - | - | - | - | 0 | +++(+) |
| Breast | Invasive ductal carcinoma | T2N1M0 | 3 | IIB | - | - | 3+ | +~++ |
| Lymph node | Metastatic carcinoma | - | - | - | - | - | 3+ | +++ |
| Breast | Invasive ductal carcinoma | T2N2M0 | 3 | IIIA | - | - | 3+ | ++ |
| Lymph node | Metastatic carcinoma | - | - | - | - | - | 3+ | +++ |
| Breast | Invasive ductal carcinoma | T3N1M0 | 3 | IIIA | ++ | ++ | 0 | +++ |
| Lymph node | Metastatic carcinoma (chronic inflammation with fibrous tissue and blood vessel) | - | - | - | - | - | 0 | +++ |
| Breast | Invasive ductal carcinoma | T2N1M0 | 3 | IIB | +++ | ++ | 2+ | - |
| Lymph node | Metastatic carcinoma | - | - | - | - | - | 0 | - |
| Breast | Invasive ductal carcinoma | T2N1M0 | 3 | IIB | ++ | - | 0 | ++ |
| Lymph node | Metastatic carcinoma | - | - | - | ++ | - | 0 | ++ |
| Breast | Invasive ductal carcinoma | T2N1M0 | 3 | IIB | - | - | 1+ | - |
| Lymph node | Metastatic carcinoma | - | - | - | - | - | 1+ | - |
| Breast | Invasive ductal carcinoma | T2N1M0 | 3 | IIB | + | ++ | 0 | +~++ |
| Lymph node | Metastatic carcinoma | - | - | - | - | ++ | 0 | ++ |
| Breast | Invasive ductal carcinoma | T2N1M0 | 3 | IIB | - | - | 3+ | ++ |
| Lymph node | Metastatic carcinoma | - | - | - | + | - | 3+ | +~++ |
| Breast | Invasive ductal carcinoma | T1N1M0 | 3 | IIA | +++ | - | 0 | ++ |
| Lymph node | Metastatic carcinoma (lymph node tissue) | - | - | - | - | - | 0 | ++ |
| Breast | Invasive ductal carcinoma | T2N1M0 | 3 | IIB | - | - | 1+ | +++ |
| Lymph node | Metastatic carcinoma | - | - | - | - | - | 1+ | +++ |
| Breast | Invasive ductal carcinoma | T2N2M0 | 3 | IIIA | - | - | 0 | +++ |
| Lymph node | Metastatic carcinoma | - | - | - | - | - | 0 | ++ |
| Breast | Invasive ductal carcinoma | T2N2M0 | 3 | IIIA | - | - | 1+ | +++ |
| Lymph node | Metastatic carcinoma (lymph node tissue) | - | - | - | + | - | 1+ | ++ |
| Breast | Invasive ductal carcinoma (breast tissue) | T2N1M0 | - | IIB | - | - | * | + |
| Lymph node | Metastatic carcinoma | - | - | - | - | - | 3+ | +++ |
| Breast | Invasive ductal carcinoma | T2N2M0 | 3 | IIIA | - | - | 3+ | +++ |
| Lymph node | Metastatic carcinoma | - | - | - | +++ | ++ | 2+ | ++ |
| Breast | Invasive ductal carcinoma | T2N1M0 | 1 | IIB | - | - | 3+ | + |
| Lymph node | Metastatic carcinoma | - | - | - | - | - | 3+ | ++ |
| Breast | Invasive ductal carcinoma | T2N2M0 | 3 | IIIA | + | - | 0 | ++ |
| Lymph node | Metastatic carcinoma | - | - | - | ++ | - | 0 | ++ |
| Breast | Invasive ductal carcinoma | T2N1M0 | 3 | IIB | - | - | 0 | +++ |
| Lymph node | Metastatic carcinoma | - | - | - | - | - | 0 | +++ |
| Breast | Invasive ductal carcinoma | T2N1M0 | 3 | IIB | - | - | 3+ | ++ |
| Lymph node | Metastatic carcinoma (necrosis tissue) | - | - | - | * | - | * | ++ |
| Breast | Invasive ductal carcinoma | T2N2M0 | 3 | IIIA | - | - | 0 | +++ |
| Lymph node | Metastatic carcinoma | - | - | - | - | - | 1+ | +++(+) |
| Breast | Invasive ductal carcinoma | T2N1M0 | 3 | IIB | - | - | 1+ | +++ |
| Lymph node | Metastatic carcinoma | - | - | - | - | - | 2+ | ++ |
| Breast | Invasive ductal carcinoma | T3N2M0 | 3 | IIIA | - | * | * | + |
| Lymph node | Metastatic carcinoma | - | - | - | - | * | * | + |
| Breast | Invasive ductal carcinoma | T3N1M0 | 3 | IIIA | ++ | + | 1+ | ++ |
| Lymph node | Metastatic carcinoma | - | - | - | + | - | 1+ | +++ |
| Breast | Invasive ductal carcinoma | T2N1M0 | 3 | IIB | - | - | 2+ | +++ |
| Lymph node | Metastatic carcinoma | - | - | - | - | ++ | 0 | +++ |
| Breast | Invasive ductal carcinoma (sparse) | T2N1M0 | 3 | IIB | - | - | 0 | +++ |
| Lymph node | Metastatic carcinoma | - | - | - | - | - | 3+ | ++ |
| Breast | Invasive ductal carcinoma | T2N1M0 | 3 | IIB | - | - | 0 | ++ |
| Lymph node | Metastatic carcinoma | - | - | - | - | - | 0 | ++~+++ |
| Breast | Invasive ductal carcinoma | T2N1M0 | 3 | IIA | - | - | 0 | ++~+++ |
| Lymph node | Metastatic carcinoma | - | - | - | - | - | 0 | ++~+++ |
| Breast | Invasive ductal carcinoma | T2N1M0 | 3 | IIB | +++ | - | 0 | +++ |
| Lymph node | Metastatic carcinoma (metastatic carcinoma of fibrofatty tissue) | - | - | - | +++ | - | 0 | +~++ |
| Breast | Invasive ductal carcinoma | T4N1M0 | 3 | IIIB | ++ | + | 0 | ++ |
| Lymph node | Metastatic carcinoma | - | - | - | +++ | +++ | 0 | +++(+) |
| Breast | Invasive ductal carcinoma | T2N1M0 | 3 | IIB | - | - | 2+ | +++ |
| Lymph node | Metastatic carcinoma | - | - | - | - | - | 0 | +++ |
| Breast | Invasive ductal carcinoma | T2N2M0 | 3 | IIIA | +++ | + | 1+ | ++ |
| Lymph node | Metastatic carcinoma | - | - | - | +++ | + | 1+ | ++~+++ |
| Breast | Invasive ductal carcinoma | T1N1M0 | 3 | IIA | +++ | - | 0 | ++~+++ |
| Lymph node | Metastatic carcinoma | - | - | - | +++ | - | 0 | +++ |
| Breast | Invasive ductal carcinoma | T2N1M0 | 3 | IIB | - | - | 0 | +++ |
| Lymph node | Metastatic carcinoma | - | - | - | - | - | 0 | +++ |
| Breast | Invasive lobular carcinoma | T2N1M0 | - | IIB | - | - | 0 | ++ |
| Lymph node | Metastatic carcinoma | - | - | - | - | - | 0 | +++ |
| Breast | Invasive lobular carcinoma | T2N1M0 | - | IIB | + | - | 2+ | ++ |
| Lymph node | Metastatic carcinoma | - | - | - | + | - | 2+ | ++~+++ |
| Breast | Invasive lobular carcinoma | T2N2M0 | - | IIIA | - | - | 3+ | +++ |
| Lymph node | Metastatic carcinoma | - | - | - | - | - | 3+ | +++(+) |
| Breast | Invasive lobular carcinoma | T2N1M0 | - | IIB | - | - | 0 | ++~+++ |
| Lymph node | Metastatic carcinoma | - | - | - | - | - | 0 | +++ |
| Breast | Invasive lobular carcinoma | T2N1M0 | - | IIB | + | - | 0 | ++ |
| Lymph node | Metastatic carcinoma | - | - | - | ++ | - | 0 | +~++ |
| Breast | Invasive lobular carcinoma | T2N1M0 | - | IIB | - | - | 0 | ++ |
| Lymph node | Metastatic carcinoma | - | - | - | - | - | 0 | ++~+++ |
| Breast | Invasive lobular carcinoma | T2N1M0 | - | IIB | - | - | 0 | ++~+++ |
| Lymph node | Metastatic carcinoma | - | - | - | - | - | 0 | +++ |
| Breast | Invasive lobular carcinoma (fibrofatty tissue and blood vessel) | T2N1M0 | - | IIB | - | - | * | + |
| Lymph node | Metastatic carcinoma | - | - | - | - | - | 0 | +++ |
| Breast | Medullary carcinoma | T3N1M0 | - | IIIA | - | - | 0 | +++ |
| Lymph node | Metastatic carcinoma | - | - | - | - | - | 0 | +++ |
| Breast | Invasive micropapillary carcinoma | T2N1M0 | - | IIB | ++ | + | 2+ | ++ |
| Lymph node | Metastatic carcinoma | - | - | - | ++ | + | 2+ | ++~+++ |
| Breast | Mixed carcinoma (invasive ductal carcinoma and invasive lobular carcinoma ) | T3N2M0 | - | IIIA | ++ | - | 2+ | +~++ |
| Lymph node | Metastatic carcinoma | - | - | - | ++ | - | 2+ | +++ |
| Breast | Mixed carcinoma (invasive ductal carcinoma and invasive lobular carcinoma ) | T1N1M0 | - | IIA | ++ | +++ | 2+ | ++ |
| Lymph node | Metastatic carcinoma | - | - | - | ++ | +++ | 2+ | +++(+) |
| Breast | Mixed carcinoma (invasive ductal carcinoma and invasive lobular carcinoma ) | T2N1M0 | - | IIB | + | - | 3+ | + |
| Lymph node | Metastatic carcinoma | - | - | - | - | - | 3+ | +++ |
| Breast | Mixed carcinoma (invasive ductal carcinoma and invasive lobular carcinoma ) | T2N1M0 | - | IIB | ++ | - | 2+ | +++ |
| Lymph node | Metastatic carcinoma | - | - | - | ++ | - | 2+ | +++ |
| Breast | Mixed carcinoma (invasive ductal carcinoma and invasive lobular carcinoma ) | T2N2M0 | - | IIIA | - | - | 3+ | ++ |
| Lymph node | Metastatic carcinoma | - | - | - | - | - | 3+ | ++ |
| Breast | Mixed carcinoma (invasive ductal carcinoma and invasive lobular carcinoma ) | T2N1M0 | - | IIB | - | - | 0 | + |
| Lymph node | Metastatic carcinoma | - | - | - | - | - | 0 | ++~+++ |
| Breast | Mixed carcinoma (invasive ductal carcinoma and invasive lobular carcinoma ) | T2N2M0 | - | IIIA | ++ | ++ | 0 | ++~+++ |
| Lymph node | Metastatic carcinoma | - | - | - | ++ | ++ | 0 | ++ |
| Breast | Mixed carcinoma (sparse invasive ductal carcinoma and invasive lobular carcinoma ) | T2N2M0 | - | IIIA | - | - | * | ++~+++ |
| Lymph node | Metastatic carcinoma | - | - | - | - | - | 3+ | ++~+++ |
|  |  |  |  |  |  |  | Control | - |


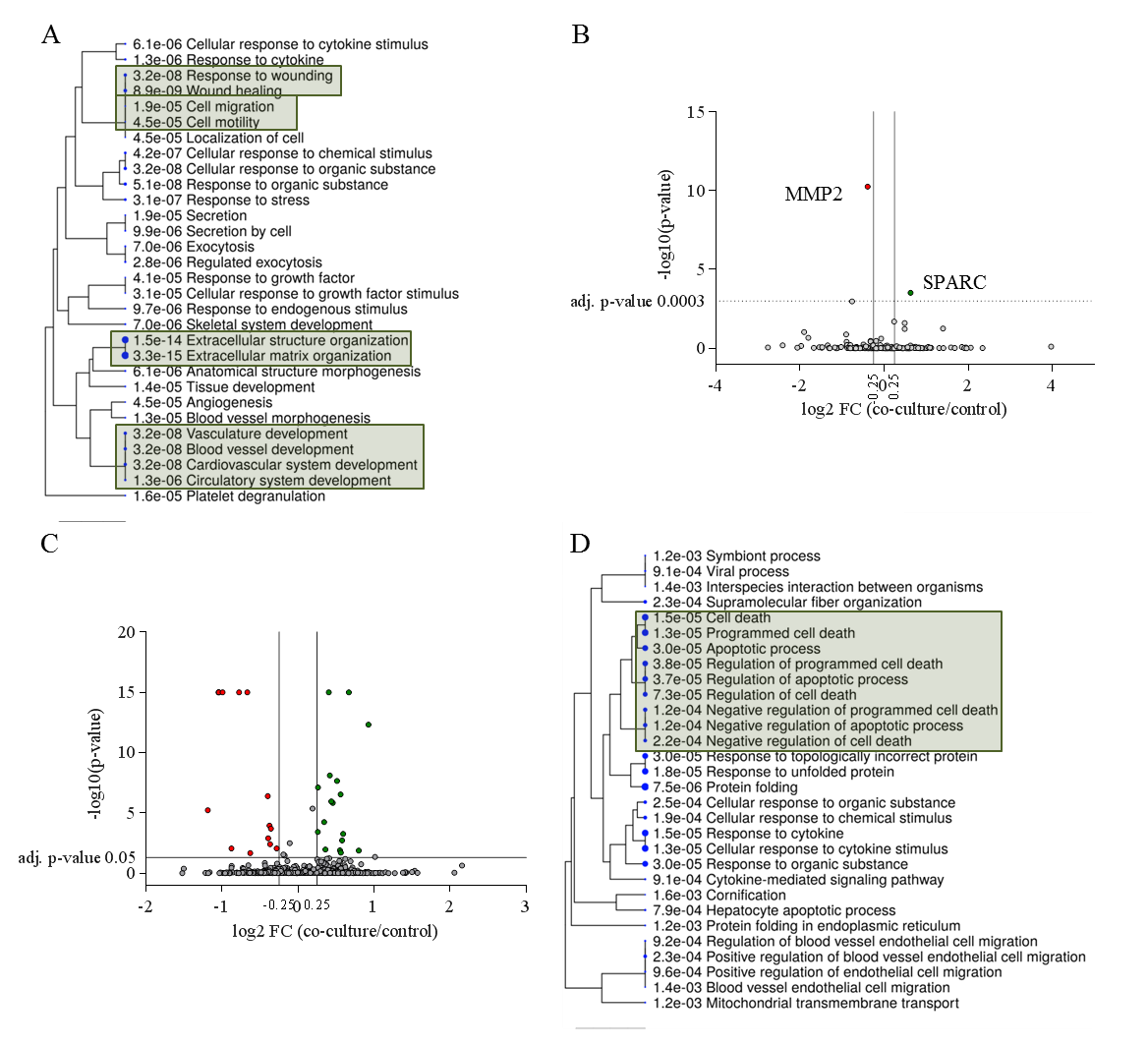


Supplementary figure 1 Functional analysis of detected discoveries from secretome and proteome analysis of co-cultured breast cancer cells. A Hierarchical clustering tree using shiny GO v06.0 Gene ontology enrichment of discoveries from co-culture media with corresponding p-values. B Volcano plot demonstrating potential bone-directed breast cancer invasiveness related targets using proteome analysis of media from co-cultures MCF-7 cells vs MG-63 control. Detected target proteins were stated as discovery when adjusted p-value was below 0.03 (dotted line) with a false-discovery rate (FDR) of 5% and a log 2 fold change (FC) higher 0.25 or lower -0.25. Every dot indicates one target, green dots indicate upregulated discoveries and red dot indicates downregulated discoveries. n=6, discovery determined using two-stage linear step-up procedure of Benjamini, Krieger and Yekutieli, with Q = 5%. Each row was analyzed individually, without assuming a consistent SD. C Volcano plot demonstrating potential bone-directed breast cancer invasiveness related targets using proteome analysis of lysates from co-cultures MCF-7 cells vs MCF-7 control. Detected target proteins were stated as discovery when adjusted p-value was below 0.05 (dotted line) with a false-discovery rate (FDR) of 1% and a log 2 fold change (FC) higher 0.25 or lower -0.25. Every dot indicates one target, green dots indicate upregulated discoveries and red dot indicates downregulated discoveries. n=6, discovery determined using two-stage linear step-up procedure of Benjamini, Krieger and Yekutieli, with Q = 1%. Each row was analyzed individually, without assuming a consistent SD. D Hierarchical clustering tree using shiny GO v06.0 Gene ontology enrichment of discoveries from co-culture lysates with corresponding p-values.


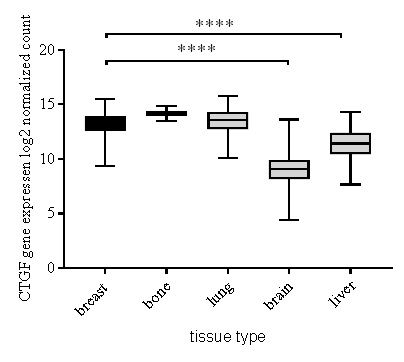


Supplementary figure 2: Tissue expression analysis of CTGF. CTGF expression in human bone (n=2), breast (n=179), lung (n=287) and brain (n=1136) tissue was assessed using xenabrowse with datasets from GTEX, TARGET, and TCGA. One-way ANOVA and a Dunnett‘s multiple comparison test with no matching or pairing between groups was calculated to assess significant differences compared to the untreated control. ***** P <0.0001*

**
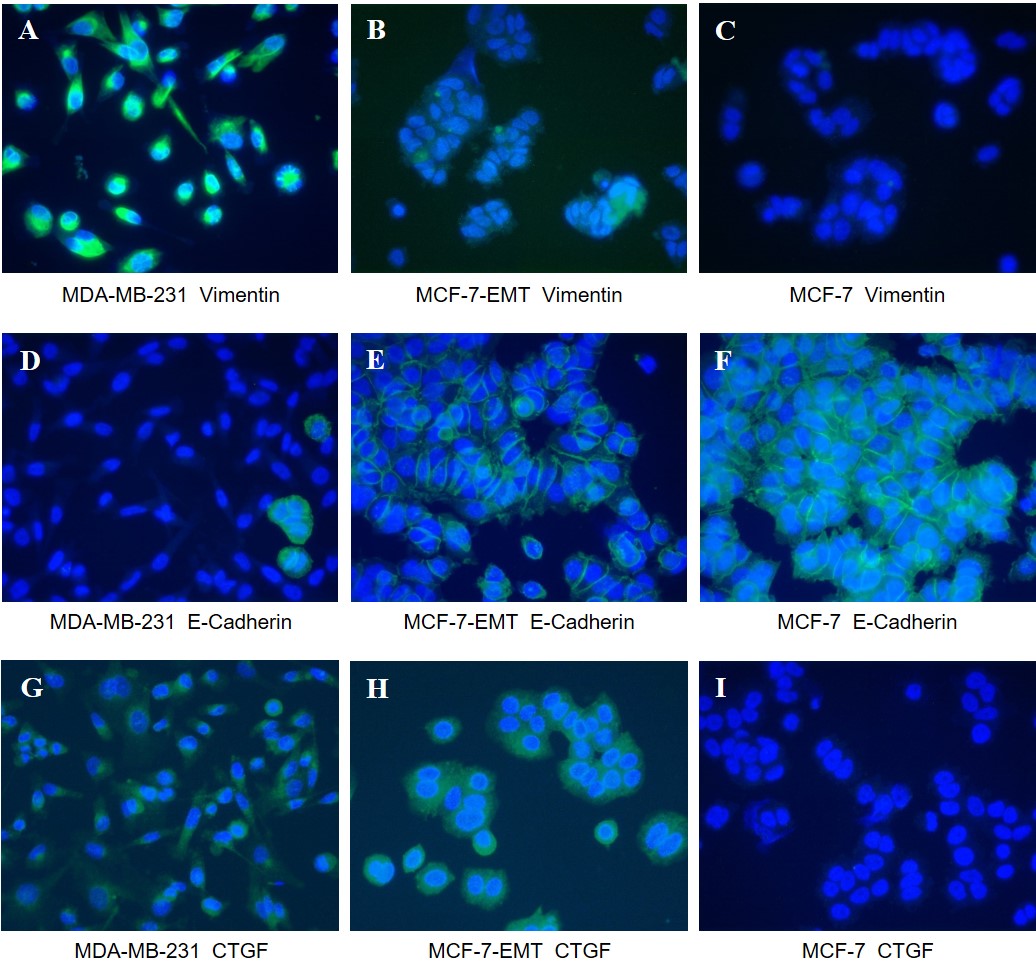
**

**Supplementary figure 3:** Expression of VIM (a-c), CDH1 (d-f), and CTGF (g-i) in MDA-MB-231 (a, d, g), mesenchymal transformed MCF-7-EMT (b, e, h), and in MCF-7 (c, f, i) breast cancer cells.


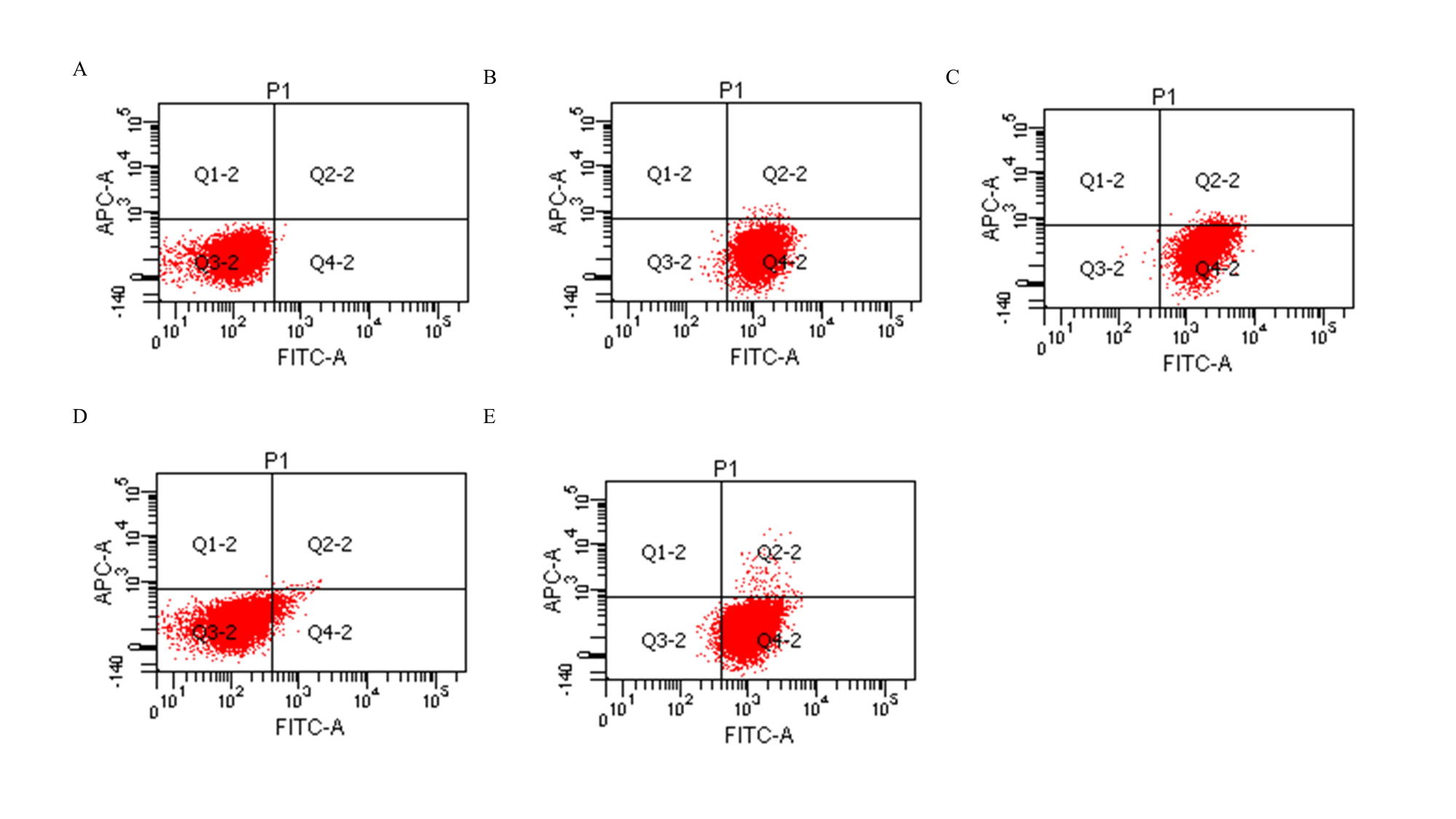


Supplementary figure 4: Histograms flow cytometry. A MCF-7 unstained control. B MCF-7. C MCF-7-EMT. D MDA-MB-231 unstained control. E MDA-MB-231. APC staining for VCAM-1, FITC staining for CD51/E-cadherin


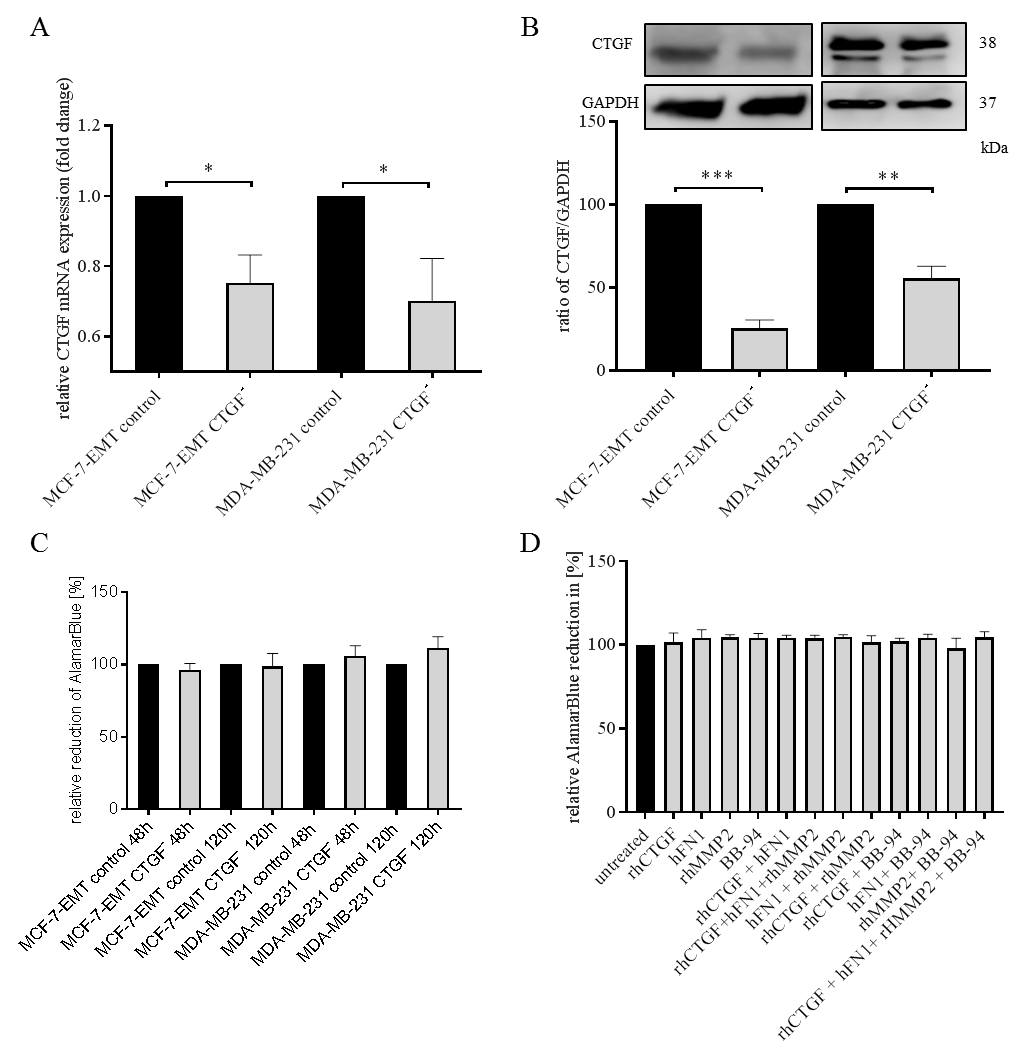


Supplementary figure 5: CTGF expression does not alter proliferation *in vitro*. A CTGF mRNA expression in different breast cancer cell lines 120 hours after siRNA transfection was detected by real-time quantitative PCR. Data represent the mean ± SEM. MCF-7-EMT n=4, MDA-MB-231 n=5 using unpaired, two-tailed t-test analysis compared to respective control. ** P<0.05* B CTGF protein expression of different breast cancer cells 48 hours after CTGF siRNA transfection was detected by western blotting. The CTGF band intensity was quantified by densitometry and normalized to GAPDH. Data represent the mean ± SEM. n=3 using unpaired, two-tailed t-test analysis compared to respective control. *** P<0.01; *** P<0.005* C Relative AlamarBlue reduction in different breast cancer cell lines 48 and 120 hours after CTGF siRNA transfection at 4 hours AlamarBlue incubation. Data represent the mean ± SEM. MCF-7-EMT t48h n=3, MCF-7-EMT t120h n=4 and MDA-MB-231 n=3 using unpaired, two-tailed t-test analysis compared to respective control. D 3D spheroid invasion assay with different compounds supplemented. Spheroids were embedded in Matrigel, after 48 hours AlamarBlue was added and absorption was measured after 4hours incubation. n = 3


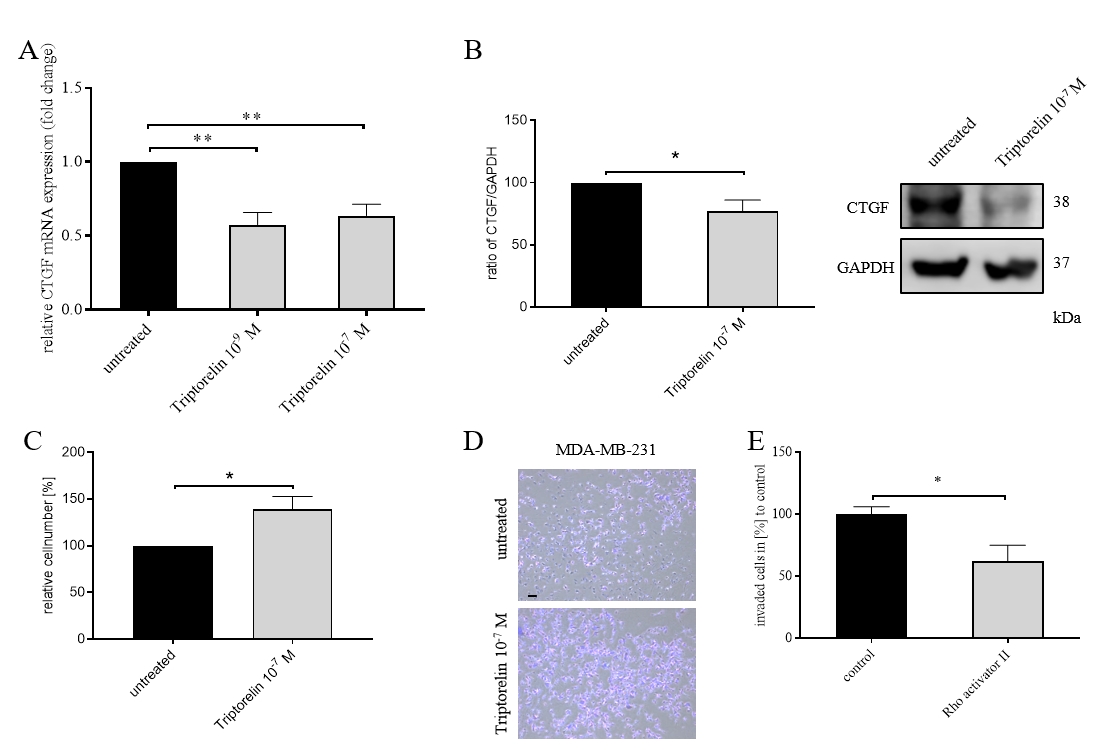


Supplementary figure 6: GnRH agonist regulates CTGF expression in TNBC cells. A Relative quantification of CTGF mRNA expression in TNBC cells (MDA-MB-231) treated for 48 hours with 10 ^-9^ M or 10 ^-7^ M Triptorelin. Data represent the mean ± SEM. MDA-MB-231 n=4 using one-way ANOVA with F= 12.29 and a Dunnett‘s multiple comparison test with no matching or pairing between groups. *** P <0.01* B Quantification and representative experiment of CTGF protein expression after Triptorelin treatment for 48 hours (10 ^-7^ M). The CTGF band intensity was quantified by densitometry and normalized to GAPDH. Data represent the mean ± SEM. MDA-MB-231 n=7 using unpaired, two-tailed t-test analysis to respective control (untreated). ** P<0.05* C Cell-ECM adhesion analysis of TNBC cells treated with 10 ^-7^ M Triptorelin. Adhesive cells where counter-stained with crystal violet and absorption was measured at 570nm. Data represent the mean ± SEM. MDA-MB-231 n=3 using unpaired, two-tailed t-test analysis to respective control (untreated). **P<0.05* D Representative *images corresponding to C.* E Following RhoA activator II treatment (1µg/ml) invaded MDA-MB-231 cells under the filter were counted in four randomly selected regions, using a co-culture Matrigel invasion assay for 48 hours. Data represent the mean ± SEM. n=9 Using unpaired, two-tailed t-test analysis to respective control. ** P< 0.05*


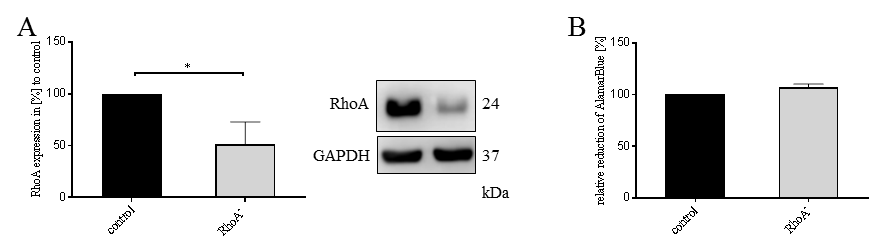


Supplementary figure 7: Reducing RhoA expression does not alter proliferation *in vitro*. (A) RhoA protein expression in MCF-7 breast cancer cells 48 hours after RhoA siRNA transfection was detected by western blotting. The RhoA band intensity was quantified by densitometry and normalized to GAPDH. Data represent the mean ± SEM. n=3 using unpaired, two-tailed t-test analysis compared to respective control. ** P<0.05* (B) Relative AlamarBlue reduction in MCF-7 breast cancer cells 48 hours after RhoA siRNA transfection at 4 hours AlamarBlue incubation. Data represent the mean ± SEM. n=3 using unpaired, two-tailed t-test analysis compared to respective control.

**Full-length gels / blots: figures 3b, 8b, 8f and supplemental figures 12b, 13b, 14a**


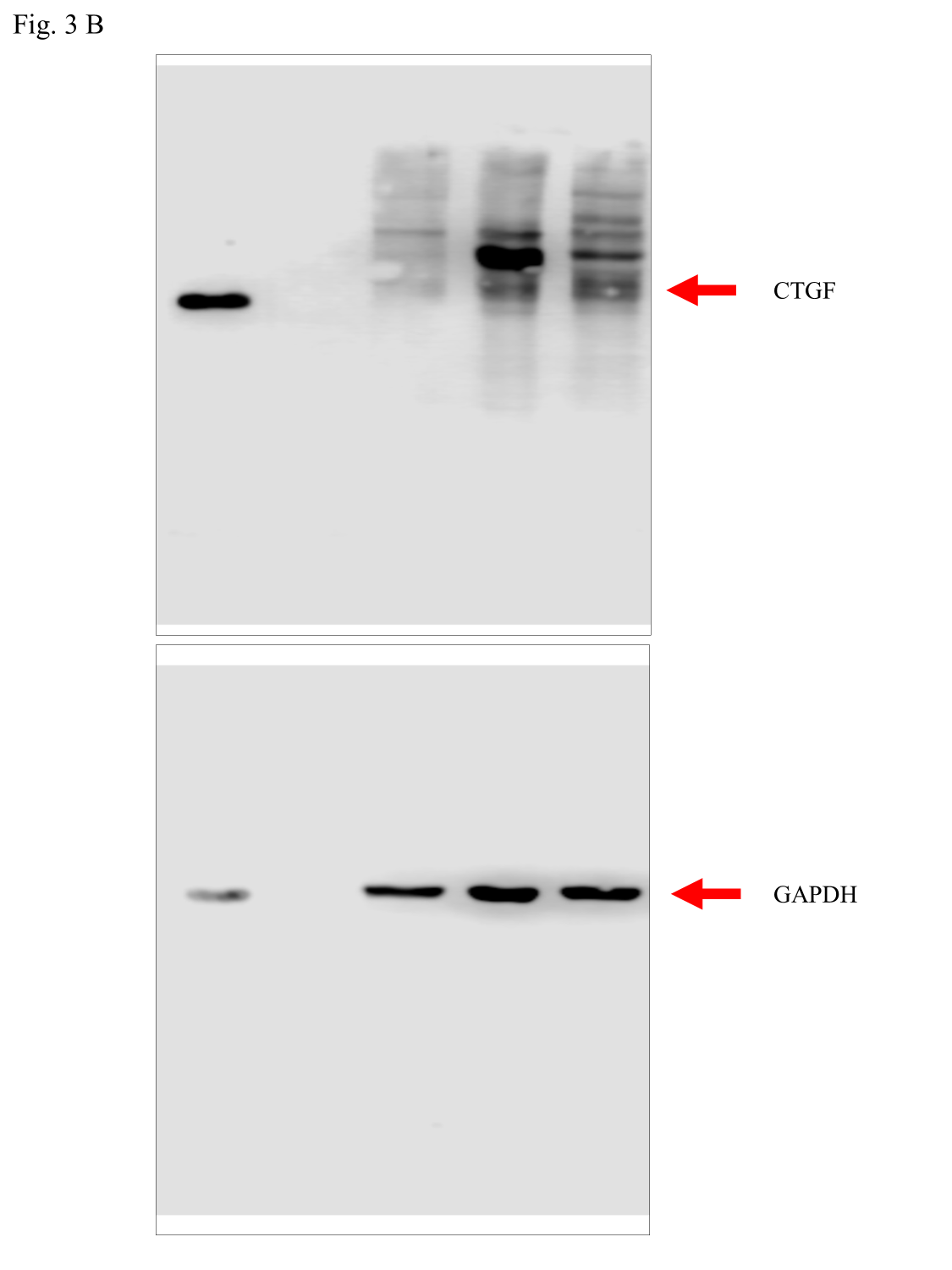


**
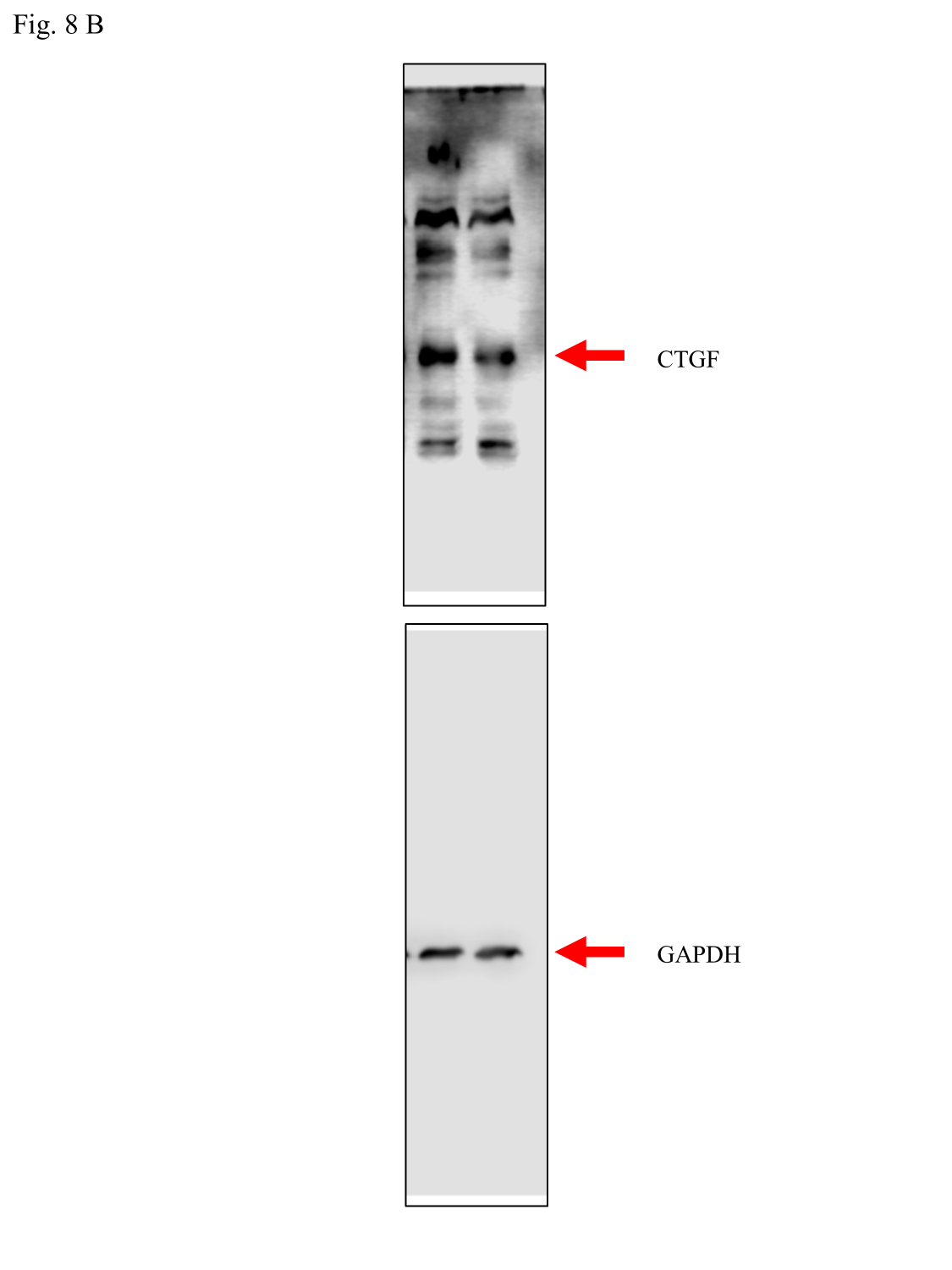
**

**
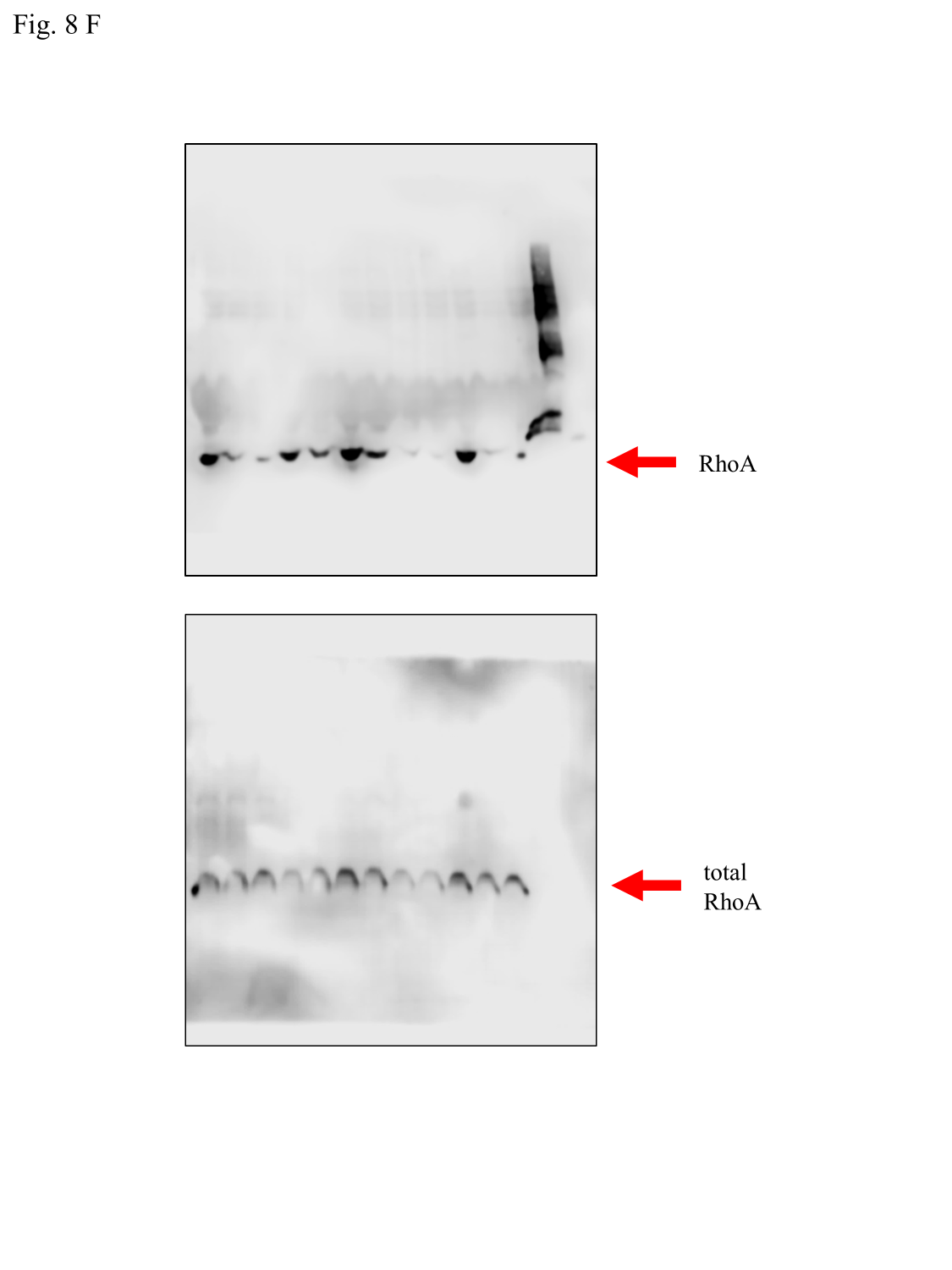
**

**
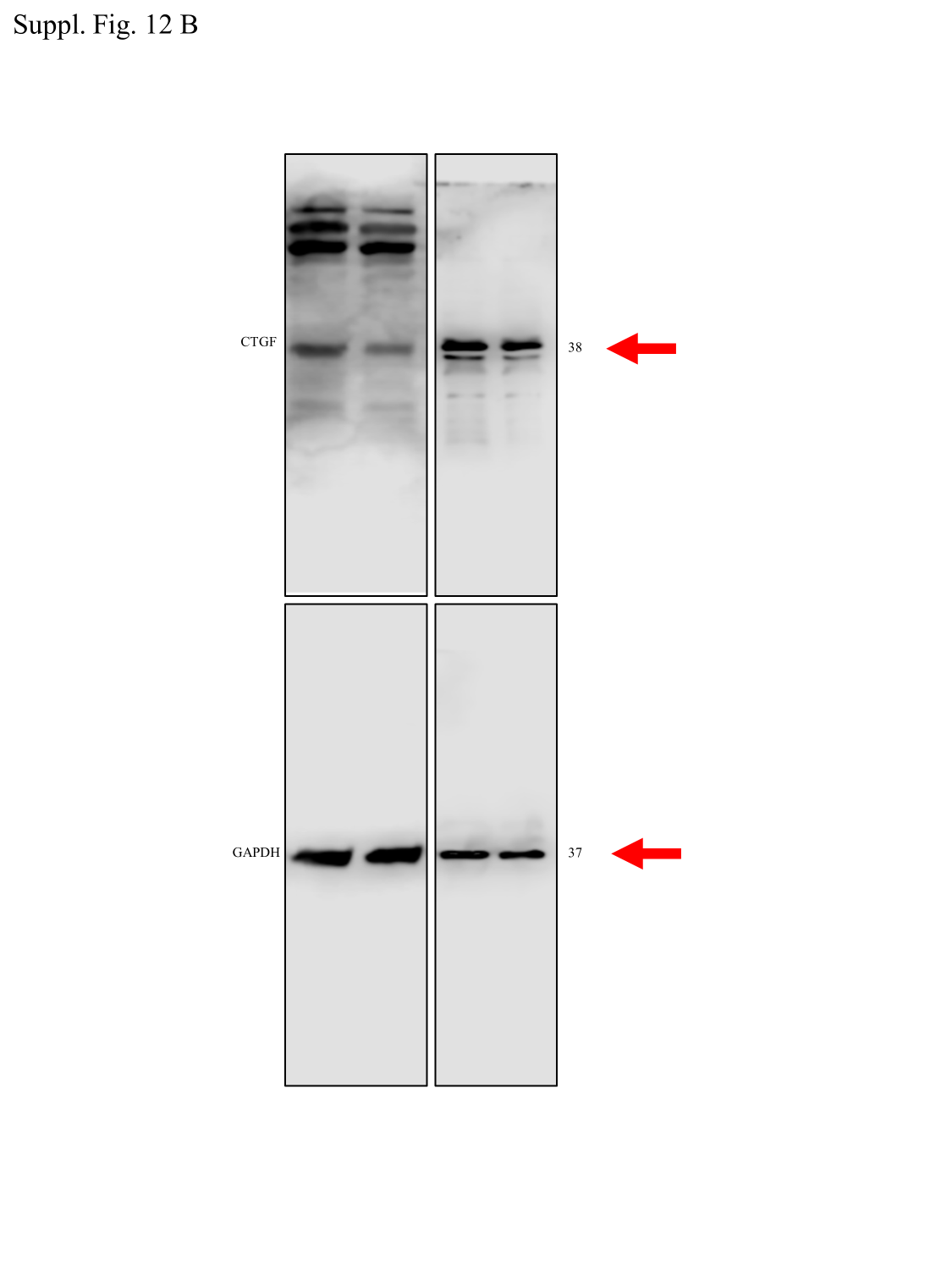
**

**
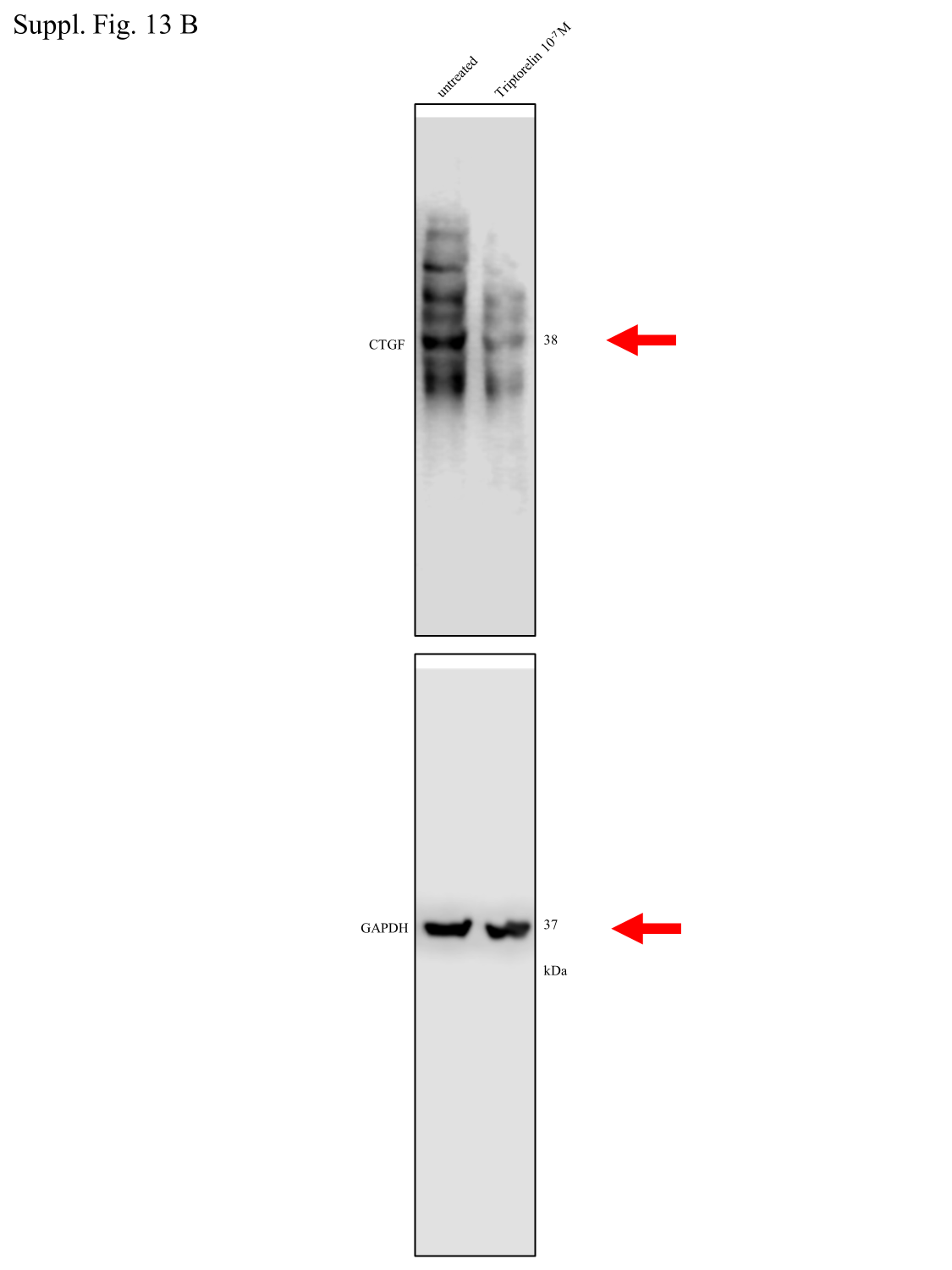
**

**
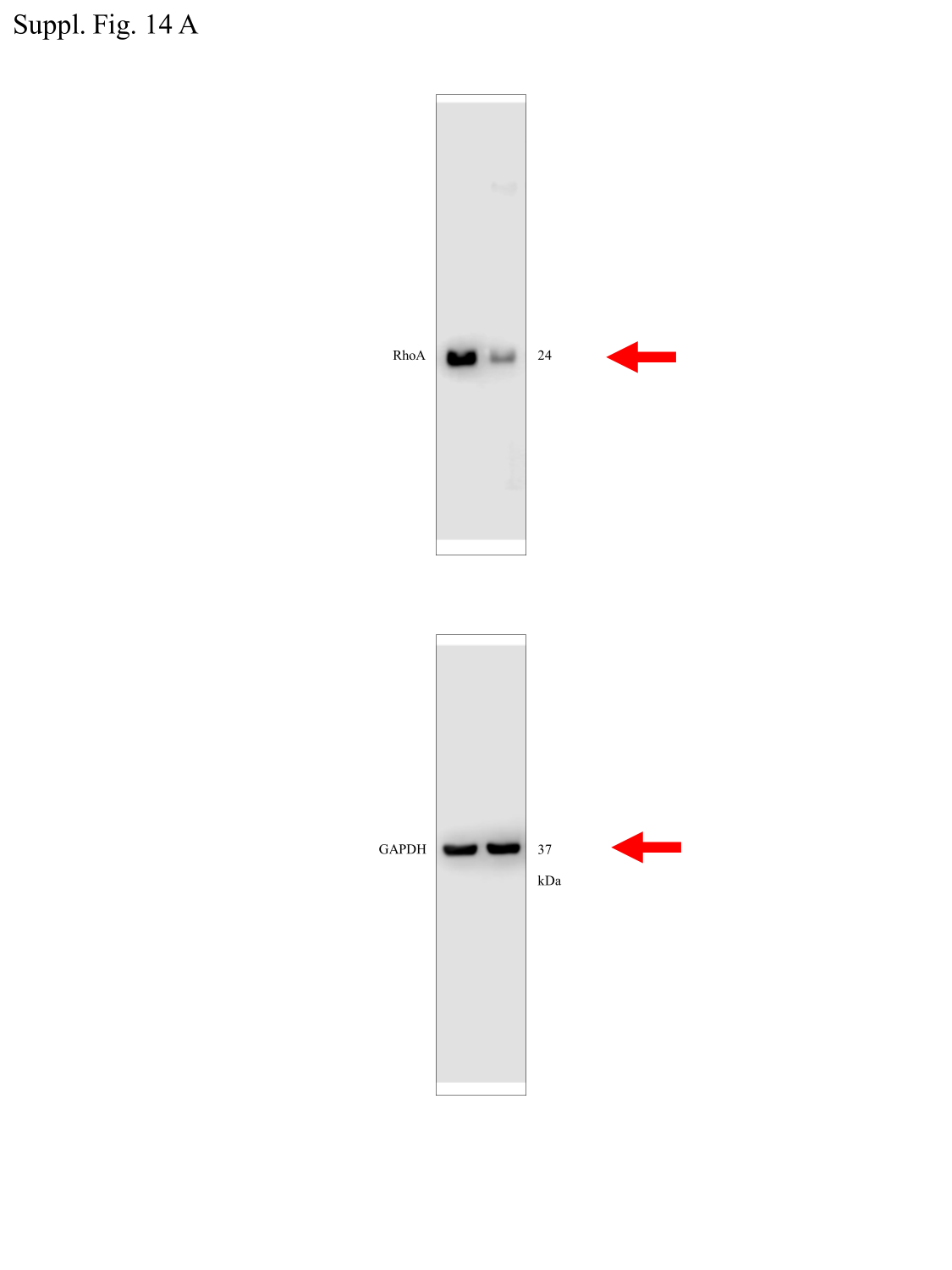
**
